# The burden of mental disorders across the states of India: the Global Burden of Disease Study 1990–2017

**DOI:** 10.1016/S2215-0366(19)30475-4

**Published:** 2020-02

**Authors:** India State-Level Disease Burden Initiative Mental Disorders Collaborators, Rajesh Sagar, Rakhi Dandona, Gopalkrishna Gururaj, R S Dhaliwal, Aditya Singh, Alize Ferrari, Tarun Dua, Atreyi Ganguli, Mathew Varghese, Joy K Chakma, G Anil Kumar, K S Shaji, Atul Ambekar, Thara Rangaswamy, Lakshmi Vijayakumar, Vivek Agarwal, Rinu P Krishnankutty, Rohit Bhatia, Fiona Charlson, Neerja Chowdhary, Holly E Erskine, Scott D Glenn, Varsha Krish, Ana M Mantilla Herrera, Parul Mutreja, Christopher M Odell, Pramod K Pal, Sanjay Prakash, Damian Santomauro, D K Shukla, Ravinder Singh, R K Lenin Singh, J S Thakur, Akhil S ThekkePurakkal, Chris M Varghese, K Srinath Reddy, Soumya Swaminathan, Harvey Whiteford, Hendrik J Bekedam, Christopher J L Murray, Theo Vos, Lalit Dandona

## Abstract

**Background:**

Mental disorders are among the leading causes of non-fatal disease burden in India, but a systematic understanding of their prevalence, disease burden, and risk factors is not readily available for each state of India. In this report, we describe the prevalence and disease burden of each mental disorder for the states of India, from 1990 to 2017.

**Methods:**

We used all accessible data from multiple sources to estimate the prevalence of mental disorders, years lived with disability (YLDs), and disability-adjusted life-years (DALYs) caused by these disorders for all the states of India from 1990 to 2017, as part of the Global Burden of Diseases, Injuries, and Risk Factors Study. We assessed the heterogeneity and time trends of mental disorders across the states of India. We grouped states on the basis of their Socio-demographic Index (SDI), which is a composite measure of per-capita income, mean education, and fertility rate in women younger than 25 years. We also assessed the association of major mental disorders with suicide deaths. We calculated 95% uncertainty intervals (UIs) for the point estimates.

**Findings:**

In 2017, 197·3 million (95% UI 178·4–216·4) people had mental disorders in India, including 45·7 million (42·4–49·8) with depressive disorders and 44·9 million (41·2–48·9) with anxiety disorders. We found a significant, but modest, correlation between the prevalence of depressive disorders and suicide death rate at the state level for females (r^2^=0·33, p=0·0009) and males (r^2^=0·19, p=0·015). The contribution of mental disorders to the total DALYs in India increased from 2·5% (2·0–3·1) in 1990 to 4·7% (3·7–5·6) in 2017. In 2017, depressive disorders contributed the most to the total mental disorders DALYs (33·8%, 29·5–38·5), followed by anxiety disorders (19·0%, 15·9–22·4), idiopathic developmental intellectual disability (IDID; 10·8%, 6·3–15·9), schizophrenia (9·8%, 7·7–12·4), bipolar disorder (6·9%, 4·9–9·6), conduct disorder (5·9%, 4·0–8·1), autism spectrum disorders (3·2%, 2·7–3·8), eating disorders (2·2%, 1·7–2·8), and attention-deficit hyperactivity disorder (ADHD; 0·3%, 0·2–0·5); other mental disorders comprised 8·0% (6·1–10·1) of DALYs. Almost all (>99·9%) of these DALYs were made up of YLDs. The DALY rate point estimates of mental disorders with onset predominantly in childhood and adolescence (IDID, conduct disorder, autism spectrum disorders, and ADHD) were higher in low SDI states than in middle SDI and high SDI states in 2017, whereas the trend was reversed for mental disorders that manifest predominantly during adulthood. Although the prevalence of mental disorders with onset in childhood and adolescence decreased in India from 1990 to 2017, with a stronger decrease in high SDI and middle SDI states than in low SDI states, the prevalence of mental disorders that manifest predominantly during adulthood increased during this period.

**Interpretation:**

One in seven Indians were affected by mental disorders of varying severity in 2017. The proportional contribution of mental disorders to the total disease burden in India has almost doubled since 1990. Substantial variations exist between states in the burden from different mental disorders and in their trends over time. These state-specific trends of each mental disorder reported here could guide appropriate policies and health system response to more effectively address the burden of mental disorders in India.

**Funding:**

Bill & Melinda Gates Foundation; and Indian Council of Medical Research, Department of Health Research, Ministry of Health and Family Welfare, Government of India.

## Introduction

Mental disorders were the second leading cause of disease burden in terms of years lived with disability (YLDs) and the sixth leading cause of disability-adjusted life-years (DALYs) in the world in 2017, posing a serious challenge to health systems, particularly in low-income and middle-income countries.^1^ Mental health is being recognised as one of the priority areas in health policies around the world and has also been included in the Sustainable Development Goals.^2^, ^3^, ^4^

**Research in context**

**Evidence before this study**

We searched PubMed for published papers on mental disorders in India and Google for reports in the public domain, as well as references in these papers and reports, on April 22, 2019, using the search terms “anorexia nervosa”, “anxiety disorders”, “attention-deficit disorder with hyperactivity”, “autism spectrum disorders”, “bipolar disorder”, “bulimia nervosa”, “burden”, “conduct disorder”, “DALYs”, “depressive disorders”, “major depressive disorders”, “dysthymic disorder”, “eating disorders”, “epidemiology”, “India”, “intellectual disability”, “mental disorders”, “mental health”, “morbidity”, “prevalence”, “schizophrenia”, and “trends”, without language or publication date restrictions. We found some studies on the prevalence of mental disorders done in different parts of India, including the National Mental Health Survey, but we found no systematic compilation of the state-level variations of prevalence and disability-adjusted life-years (DALYs) and their time trends, which is needed to inform the mental health policy and programmes across the country.

**Added value of this study**

To our knowledge, this study is the first to provide comprehensive estimates of the prevalence and disease burden due to all mental disorders for every state of India from 1990 to 2017, on the basis of all accessible data sources and with use of the standardised Global Burden of Diseases, Injuries, and Risk Factors Study framework. The findings highlight that mental disorders affect one in seven people in India, and their contribution to the total disease burden has almost doubled between 1990 and 2017. This Article reports variations in the prevalence of mental disorders between the states of India, with the prevalence of mental disorders of predominantly childhood and adolescent onset higher in the less developed northern states than in the more developed southern states, and the prevalence of mental disorders manifesting predominantly during adulthood higher in the more developed southern states than in the less developed northern states. This report assessed the association of major mental disorders with the suicide death rate at the state level, finding a significant, but modest, correlation with depressive disorders. The findings include a description of how the burden of individual mental disorders has changed from 1990 to 2017 in the states of India at different levels of development. Finally, this report assessed the burden of mental disorders that can be attributed to risk factors.

**Implications of all the available evidence**

This comprehensive assessment of mental disorders in every state of India from 1990 to 2017 highlights that a large proportion of India's population is affected by these disorders, emphasising an urgent need to put in place systems that can facilitate better diagnosis and management of mental disorders across the country. The state-specific findings of the burden and time trends of individual mental disorders in this report could serve as a reference for policy makers to plan approaches for curbing the growing burden of mental disorders in a systematic way in India.

Recognising the importance of mental disorders in reducing the total disease burden, India launched its first National Mental Health Policy in 2014 and a revised Mental Healthcare Act in 2017, with the objectives of providing equitable, affordable, and universal access to mental health care.^5^, ^6^ India has a federal set-up in which health is primarily a responsibility of the states.^2^ The socio-cultural and demographic diversity across the states of India requires that the policies and interventions to contain the burden of mental disorders be well suited to local contexts. Therefore, a better understanding of the distribution and trends of mental disorders for each state of India is crucial. Previous studies exist that have described the disease burden of mental disorders in India,^7^, ^8^, ^9^, ^10^, ^11^, ^12^, ^13^, ^14^, ^15^, ^16^ but a systematic understanding of the magnitude of this burden and the trends for all the states of India is not readily available. In this report, we present a detailed account of the prevalence and disease burden of each mental disorder and their associated risk factors for the states of India, from 1990 to 2017, on the basis of modelling using all accessible data sources. Our use of the word burden within this study is in line with the technical language of the Global Burden of Diseases, Injuries, and Risk Factors Study (GBD), and is not intended to imply negative judgement of individuals who experience mental health problems.

## Methods

### Overview

The analysis and findings of mental disorders presented in this Article were produced by the India State-Level Disease Burden Initiative as part of GBD 2017. The work of this Initiative has been approved by the Health Ministry Screening Committee at the Indian Council of Medical Research and the ethics committee of the Public Health Foundation of India. A comprehensive description of the metrics, data sources, and statistical modelling for mental disorders in GBD 2017 has been reported elsewhere.^3^, ^17^, ^18^, ^19^, ^20^ In GBD 2017, mental disorders included depressive disorders, anxiety disorders, schizophrenia, bipolar disorder, idiopathic developmental intellectual disability (IDID), conduct disorder, autism spectrum disorders, eating disorders, attention-deficit hyperactivity disorder (ADHD), and other mental disorders. Suicide is classified under injuries in GBD. The India State-Level Disease Burden Initiative has previously reported detailed trends of suicide deaths for all states of India.^21^ The GBD 2017 methods relevant for this Article are summarised here and described in detail in the appendix (pp 3–39).

### Estimation of prevalence, YLDs, and DALYs

Mental disorders and their types were defined on the basis of the clinical diagnostic criteria from the Diagnostic and Statistical Manual of Mental Disorders (fourth edition, text revision) or the International Classification of Diseases (tenth edition).^19^ The prevalence of mental disorders was estimated with use of DisMod-MR (version 2.1), a meta-regression computational tool for Bayesian disease modelling that is the standard GBD modelling approach for describing non-fatal health outcomes by location, age, sex, and year. This approach involved identification of all available data sources that could be accessed, estimation of cause-specific prevalence, estimation of severity distribution for sequelae, quantification of the magnitude of health loss by use of disability weights, adjustment for comorbidity, and computation of YLDs for each location, age, sex, and year.^19^ YLDs were estimated as the product of prevalence estimate and disability weights for health states of each mutually exclusive sequela, with adjustment for comorbidities.

Eating disorders were the only mental disorders in GBD 2017 to which deaths could be attributed directly.^17^, ^18^ All accessible data, including those for covariates, were used to develop a set of plausible models and eventually, the best ensemble predictive model to produce estimates of deaths and years of life lost (YLLs) due to premature mortality by location, age, sex, and year.^17^, ^18^ YLLs were computed from observed deaths and reference standard life expectancy at the age of death, which was obtained from the GBD standard life table.^17^ DALYs, a summary measure of total health loss, were computed by adding YLLs and YLDs for each cause under mental disorders.^3^ The major data inputs for estimating the prevalence of mental disorders in India were population-based surveys, including the World Health Survey 2003 for India, the National Mental Health Survey 2015–16, and other published studies (appendix pp 40–46).

### Estimation of risk factor exposure and attributable disease burden

The GBD comparative risk assessment framework was used to estimate exposure of risk factors related to mental disorders and their attributable disease burden.^20^ Exposure data for risk factors with a categorical or continuous distribution were collated from all available data sources that could be accessed, including survey and other data, adjusted by use of age-sex splitting and strengthened with the incorporation of covariates for modelling. For each risk factor, the theoretical minimum risk exposure level was established as the lowest level of risk exposure below which its relationship with a disease outcome was not supported by the available evidence. The modelling approach integrated multiple data inputs and borrowed information across age, time, and location to produce the best possible estimates of risk exposure by location, age, sex, and year.^20^ The estimation of attributable disease burden to risk factors included ascertainment of the relative risk of disease outcomes for risk exposure–disease outcome pairs with sufficient evidence of a causal relation in global literature (ie, randomised controlled trials, prospective cohorts, or case-control studies). Estimates of mean risk factor exposure were used to calculate summary exposure values for each risk, a metric ranging from 0% to 100% to describe the risk-weighted exposure for a population or risk-weighted prevalence of exposure.^20^ The estimates for summary exposure values were then combined with relative risk estimates for mental disorders with sufficient evidence of a causal relationship to calculate deaths, YLLs, YLDs, and DALYs attributable to each risk factor. A detailed description of exposure and attributable disease burden estimation for the major risk factors associated with mental disorders, including GBD exposure definitions and statistical modelling, is provided in the appendix (pp 31–39) and has been previously published.^20^ The risk factors to which the burden of mental disorders could be attributed in GBD 2017 included lead exposure, intimate partner violence, childhood sexual abuse, and bullying victimisation.^20^

GBD uses covariates that have a known association with the outcome of interest to arrive at the best possible estimate of the outcome of interest when data for the outcome are scarce but data for covariates are available. This approach was part of the estimation process for the findings presented in this Article.^3^, ^17^, ^18^, ^19^, ^20^

### Analysis presented in this Article

We report findings for 31 geographical units in India, comprising 29 states, the Union Territory of Delhi, and the union territories other than Delhi (combining the six smaller union territories of Andaman and Nicobar Islands, Chandigarh, Dadra and Nagar Haveli, Daman and Diu, Lakshadweep, and Puducherry). The states of Chhattisgarh, Uttarakhand, and Jharkhand were created from existing larger states in 2000, and the state of Telangana was created in 2014. For trends from 1990 onward, we disaggregated data for these four new states from their parent states on the basis of data from the districts that now constitute these states. The state of Jammu and Kashmir was divided into two union territories in August, 2019. Because we are reporting findings up to 2017, we report findings for the state of Jammu and Kashmir. We also present here findings for three groups of states based on the Socio-demographic Index (SDI) computed by GBD. SDI is a composite indicator of development status, ranging from 0 to 1, and is a geometric mean of the values of the indices of lag-distributed per-capita income, mean education in people aged 15 years or older, and total fertility rate in people younger than 25 years.^22^ The states were categorised into three state groups on the basis of their SDI in 2017: low SDI (≤0·53), middle SDI (0·54–0·60), and high SDI (>0·60; appendix p 47).^23^

We report the overall and age-specific and sex-specific prevalence and DALY rates in 2017 for each mental disorder for all states of India. We also report the comparison of the percentage change in prevalence of mental disorders from 1990 to 2017, with the percentage change in the DALY rates reported for India and the SDI state groups. We assessed the relationship between the prevalence of depressive disorders, anxiety disorders, schizophrenia, and bipolar disorder with the suicide death rate at the state level using correlation analysis. We present the DALYs for specific mental disorders that were attributable to risk factors in 2017.

We present both crude and age-standardised estimates as relevant. Crude estimates reflect the actual situation of each state and thus are useful for policy makers. By contrast, age-standardised estimates allow comparisons over time and across states after adjusting for the age structure of the population. The age-standardised rates were based on the GBD global reference population.^18^ Estimates are reported with 95% uncertainty intervals (UIs) wherever relevant. These intervals were based on 1000 runs of the models for each quantity of interest, with the mean considered as the point estimate and the 2·5th and 97·5th percentiles considered as the 95% UI (appendix p 39).^3^, ^18^, ^19^, ^20^

### Role of the funding source

Some of the contributors to this study work with the Indian Council of Medical Research. The other funder, the Bill & Melinda Gates Foundation, of the study had no role in the study design, data collection, data analysis, data interpretation, or writing of the report. The corresponding author had full access to all of the data in the study, and had final responsibility for the decision to submit for publication.

## Results

In 2017, there were 197·3 million (95% UI 178·5–216·4) people with mental disorders in India, comprising 14·3% of the total population of the country. Mental disorders contributed 4·7% (3·7–5·6) of the total DALYs in India in 2017, compared with 2·5% (2·0–3·1) in 1990.^24^ YLDs made up all the DALYs from mental disorders in 2017, except eating disorders, for which YLDs made up 99·8% of the DALYs. Mental disorders were the leading cause of YLDs in India, contributing 14·5% of the total YLDs in 2017.^24^ The highest contribution to DALYs due to mental disorders in India in 2017 was from depressive disorders (33·8%, 29·5–38·5) and anxiety disorders (19·0%, 15·9–22·4), followed by IDID (10·8%, 6·3–15·9), schizophrenia (9·8%, 7·7–12·4), bipolar disorder (6·9%, 4·9–9·6), and conduct disorder (5·9%, 4·0–8·1; table 1). The contribution of depressive disorders and eating disorders to the total DALYs was substantially higher in females than in males, whereas the contribution of autism spectrum disorders and ADHD was significantly higher in males than in females.

**Table 1:** Percentage of total DALYs due to each cause under mental disorders in India, 2017

		**Both sexes**	**Males**	**Females**
Depressive disorders		33·8% (29·5–38·5)	28·9% (25·0–33·3)	38·6% (34·0–43·7)
	Major depressive disorder	26·7% (22·6–31·2)	22·7% (19·0–27·0)	30·6% (26·1–35·8)
	Dysthymia	7·1% (5·7–8·7)	6·2% (4·9–7·6)	8·0% (6·4–9·7)
Anxiety disorders		19·0% (15·9–22·4)	16·2% (13·5–19·2)	21·7% (18·1–25·5)
Idiopathic developmental intellectual disability		10·8% (6·3–15·9)	11·8% (7·0–17·4)	9·7% (5·6–14·6)
Schizophrenia		9·8% (7·7–12·4)	11·2% (8·8–14·0)	8·5% (6·7–10·8)
Bipolar disorder		6·9% (4·9–9·6)	7·2% (5·1–10·0)	6·6% (4·7–9·1)
Conduct disorder		5·9% (4·0–8·1)	7·9% (5·5–10·9)	3·9% (2·6–5·6)
Autism spectrum disorders		3·2% (2·7–3·8)	4·8% (4·0–5·7)	1·7% (1·4–2·0)
Eating disorders		2·2% (1·7–2·8)	1·5% (1·1–2·0)	2·8% (2·2–3·6)
	Anorexia nervosa	0·5% (0·3–0·6)	0·2% (0·1–0·3)	0·7% (0·5–0·9)
	Bulimia nervosa	1·8% (1·3–2·3)	1·3% (1·0–1·8)	2·2% (1·6–2·8)
Attention-deficit hyperactivity disorder		0·3% (0·2–0·5)	0·5% (0·3–0·7)	0·2% (0·1–0·3)
Other mental disorders		8·0% (6·1–10·1)	9·9% (7·5–12·4)	6·3% (4·7–7·9)

Data are percentage, with 95% uncertainty interval in parentheses. DALYs=disability-adjusted life-years.

### Prevalence of mental disorders

**Figure 1:**
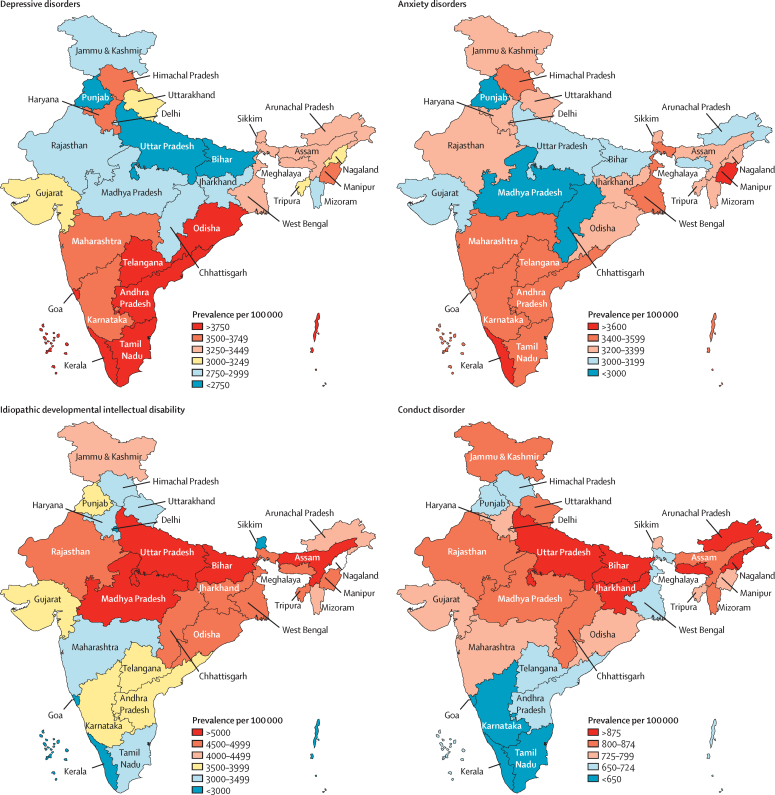
Crude prevalence of major mental disorders in the states of India, 2017 The state of Jammu and Kashmir was divided into two union territories in August 2019; as we are reporting findings up to 2017, we report findings for the state of Jammu and Kashmir.

Among the major mental disorders that manifest predominantly during adulthood, the crude prevalence for both depressive disorders and anxiety disorders was 3·3% (3·1–3·6 for depressive disorders and 3·0–3·5 for anxiety disorders), whereas bipolar disorders had prevalence of 0·6% (0·5–0·7) and schizophrenia 0·3% (0·2–0·3) (table 2). In 2017, 45·7 million (42·4–49·8) people had depressive disorders in India. Prevalence of depressive disorders varied 1·9 times among the states, with the highest prevalence observed in Tamil Nadu, Kerala, Goa, and Telangana in the high SDI state group; Andhra Pradesh in the middle SDI state group; and Odisha in the low SDI state group (figure 1, appendix p 48). The prevalence of depressive disorders was positively associated with the suicide death rate at the state level for both sexes, with a slightly higher correlation coefficient in females (r=0·57, r^2^=0·33; p=0·0009) than in males (r=0·44, r^2^=0·19; p=0·015; appendix p 49).

**Table 2:** Prevalence of mental disorders in India, 2017

	**Both sexes**	**Males**	**Females**
All mental disorders	14·3% (12·9–15·7)	14·2% (12·8–15·6)	14·4% (13·1–15·8)
Idiopathic developmental intellectual disability	4·5% (3·0–6·0)	4·7% (3·1–6·3)	4·3% (2·9–5·7)
Depressive disorders	3·3% (3·1–3·6)	2·7% (2·5–3·0)	3·9% (3·6–4·3)
Anxiety disorders	3·3% (3·0–3·5)	2·7% (2·4–2·9)	3·9% (3·6–4·3)
Conduct disorder	0·8% (0·6–1·0)	1·0% (0·8–1·3)	0·6% (0·4–0·7)
Bipolar disorder	0·6% (0·5–0·7)	0·6% (0·5–0·7)	0·6% (0·5–0·7)
Attention-deficit hyperactivity disorder	0·4% (0·3–0·5)	0·6% (0·5–0·7)	0·2% (0·2–0·3)
Autism spectrum disorders	0·4% (0·3–0·4)	0·5% (0·5–0·6)	0·2% (0·2–0·2)
Schizophrenia	0·3% (0·2–0·3)	0·3% (0·2–0·3)	0·2% (0·2–0·3)
Eating disorders	0·2% (0·1–0·2)	0·1% (0·9–1·4)	0·3% (0·2–0·3)
Other mental disorders	1·8% (1·5–2·0)	2·1% (1·8–2·4)	1·4% (1·2–1·7)

Data are percentage, with 95% uncertainty interval in parentheses.

In 2017, 44·9 million (41·2–48·9) people had anxiety disorders in India. The prevalence varied 1·4 times across the states, with the highest prevalence observed in the states of Kerala, Karnataka, Telangana, Tamil Nadu, Himachal Pradesh, and Maharasthra in the high SDI state group; and Andhra Pradesh, Manipur, and West Bengal in the middle SDI state group (figure 1, appendix p 48). We did not find a significant association of the prevalence of anxiety disorders with the suicide death rate at the state level (appendix p 49).

In 2017, 7·6 million (95% UI 6·6–9·0) people had bipolar disorder in India. The prevalence varied 1·3 times across the states, with Goa, Kerala, Sikkim, and Himachal Pradesh of the high SDI state group having the highest prevalence among all states. 3·5 million (95% UI 3·0–4·0) people had schizophrenia in India in 2017. Prevalences varied 1·6 times among states, with the highest prevalence observed in the states of Goa, Kerala, Tamil Nadu, and Delhi of the high SDI state group. We found a modest correlation between the prevalence of schizophrenia and suicide death rate in males (r=0·33, r^2^=0·11; p=0·066) and females (r=0·35, r^2^=0·12, p=0·052) which did not reach significance for both sexes (appendix p 49).

Among mental disorders that have the onset predominantly during childhood and adolescence, the crude prevalence for IDID was 4·5% (95% UI 3·0–6·0), whereas conduct disorder had prevalence of 0·8% (0·6–1·0), ADHD of 0·4% (0·3–0·5), and autism spectrum disorders of 0·4% (0·3–0·4; table 2). The prevalence of IDID varied 3·2 times across the states of India in 2017, with the highest prevalence observed in Bihar, Uttar Pradesh, Madhya Pradesh, and Assam of the low SDI state group (figure 1, appendix p 48). The prevalence of conduct disorder varied 1·7 times between the states, with the highest prevalence observed in Jharkhand, Bihar, and Uttar Pradesh of the low SDI state group; and in the north-eastern states of Meghalaya, Nagaland, and Arunchal Pradesh. The prevalence of autism spectrum disorders varied 1·4 times between the states, with the highest prevalence found in Jammu and Kashmir and Arunachal Pradesh of the middle SDI state group, and Bihar and Uttar Pradesh of the low SDI state group. The prevalence of ADHD varied 2·8 times between the states, with the highest prevalence found in Maharashtra of the high SDI state group, Meghalaya and Arunachal Pradesh of the middle SDI state group, and Bihar of the low SDI state group. The prevalence of eating disorders varied 2·1 times between the states, with the highest prevalence observed in Goa, Delhi, and Sikkim of the high SDI state group; and Haryana of the middle SDI state group.

**Figure 2:**
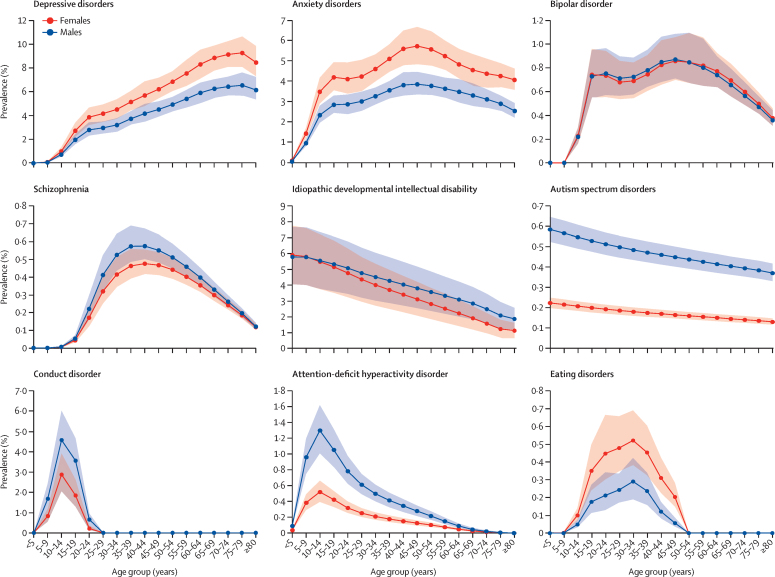
Age-specific and sex-specific prevalence of mental disorders in India, 2017 Shaded areas show 95% uncertainty intervals.

Although we found no difference in the overall prevalence of mental disorders between males and females, the prevalence of depressive disorders, anxiety disorders, and eating disorders was significantly higher in females than in males and the prevalence of conduct disorder, autism spectrum disorders, and ADHD was substantially higher in males than in females (table 2). The age-specific prevalence of depressive disorders increased with age in India in 2017, with the highest prevalence observed in older adults. This prevalence was significantly higher in females than in males, starting at 45 years (figure 2, appendix p 50). The prevalence of anxiety disorders in both sexes increased rapidly in adolescents and young adults and was higher in females than in males in most age groups. The prevalence of bipolar disorder increased during adolescence and plateaued during most of adulthood, with a slight decline in older age groups. The prevalence of schizophrenia increased swiftly in young age groups, peaked in the 35–44 years age group, and declined steadily in older age groups.

The prevalence of IDID was highest in the youngest age groups and decreased with increasing age in both sexes (figure 2, appendix p 50). The prevalence of autism spectrum disorders was highest in the youngest age groups, decreased with increasing age, and had a higher prevalence in males than in females across all ages. Conduct disorder was mainly prevalent between the ages of 5–20 years, with the peak occurring in the 10–14 years age group in both males and females; a small prevalence remained after age 18 years that included individuals who did not meet the criteria for classification as personality disorder. The prevalence of ADHD peaked in the 10–14 years age group and was significantly higher in males than in females for ages 5–64 years. The prevalence of eating disorders increased during adolescence and young adulthood, peaked at 30–34 years in both sexes, and tapered thereafter.

### DALY rate of mental disorders

**Figure 3:**
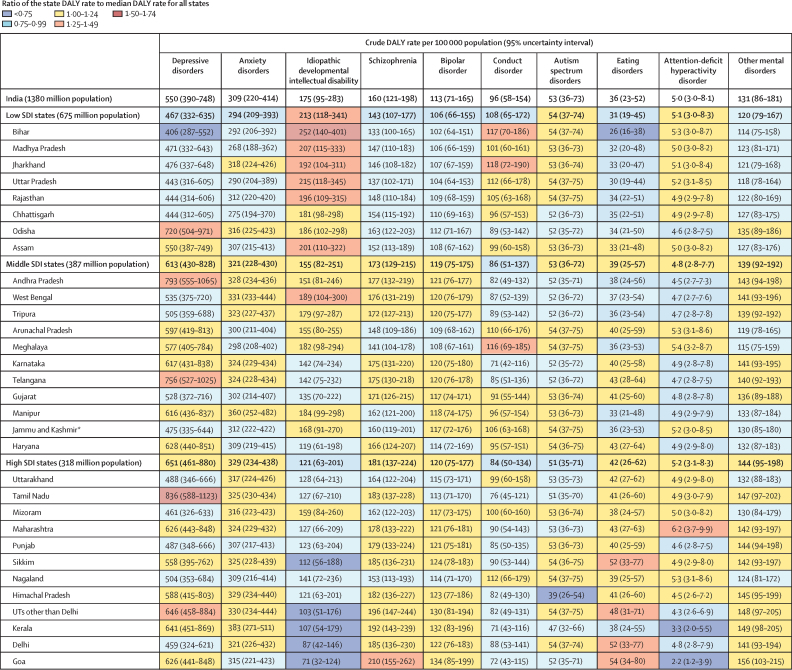
Crude DALY rates of mental disorders in the states of India grouped by SDI, 2017 DALY=disability-adjusted life-year. SDI=Socio-demographic Index. UTs=Union territories. *The state of Jammu and Kashmir was divided into two union territories in August 2019; because we are reporting findings up to 2017, we report findings for the state of Jammu and Kashmir.

There were variations between the states in the crude DALY rates of individual mental disorders, though the uncertainty intervals overlapped in many instances (figure 3). The crude DALY rate of depressive disorders varied 2·1 times between the states in 2017 and was highest in Tamil Nadu of the high SDI state group, followed by Telangana and Andhra Pradesh of the middle SDI state group, and Odisha of the low SDI state group. The crude DALY rate varied between the states 1·4 times for anxiety disorders, 1·6 times for schizophrenia, and 1·3 times for bipolar disorder. Crude DALY rates for IDID varied 3·5 times between the states, with the highest rates occurring in Bihar, Madhya Pradesh, Rajasthan, Jharkhand, Uttar Pradesh, and Assam in the low SDI state group; and in West Bengal in the middle SDI state group. Crude DALY rates varied between states 1·7 times for conduct disorder and 1·4 times for autism spectrum disorders. The DALY rates for ADHD were very low across states. Crude DALY rates for eating disorders varied 2·1 times among states in 2017, with the highest rates observed in Goa, Delhi, and Sikkim in the high SDI state group. Point estimates of DALY rates for mental disorders that have onset predominantly in childhood and adolescence were higher in the low SDI state group than in the middle and high SDI state groups, whereas point estimates of mental disorders that manifest predominantly during adulthood were lower in the low SDI state group than in the middle and high SDI state groups, though the 95% UIs overlapped (figure 3).

### Trends from 1990 to 2017

**Figure 4:**
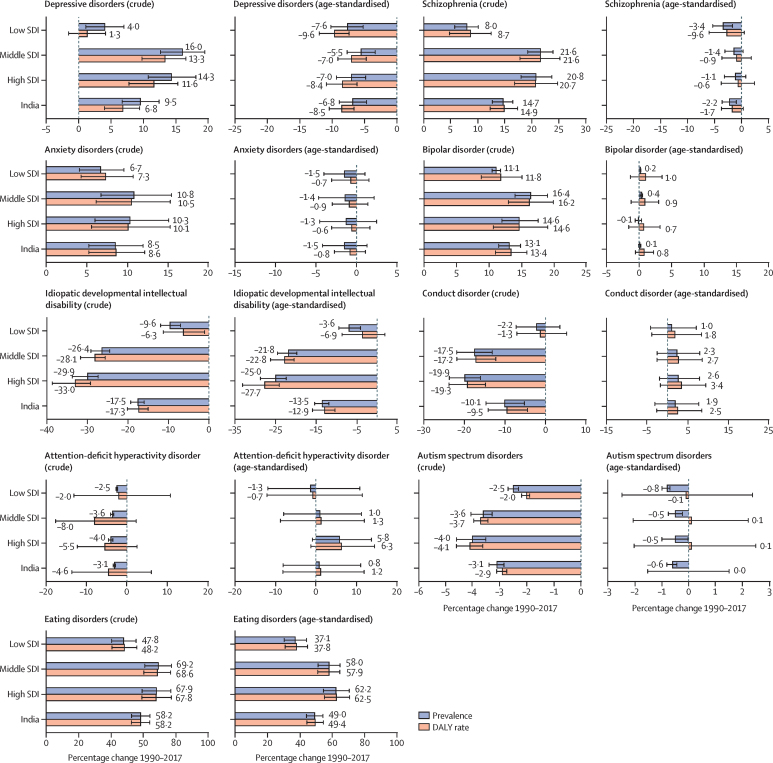
Percentage change in prevalence and DALY rate of mental disorders in the states of India grouped by SDI, 1990–2017 Error bars represent 95% uncertainty intervals. DALY=disability-adjusted life-year. SDI=Socio-demographic Index.

The crude prevalence and DALY rate of depressive disorders, anxiety disorders, bipolar disorder, and schizophrenia increased in India from 1990 to 2017 (figure 4). The increase in prevalence and DALY rate was higher in the high SDI and middle SDI state groups than in the low SDI state group for depressive disorders and schizophrenia, but no significant differences were observed between the SDI state groups for the increase in anxiety and bipolar disorders. By contrast, the age-standardised prevalence and DALY rate of depressive disorders decreased in India during this period, with similar changes in the SDI state groups, but with no significant changes in the age-standardised prevalence and DALY rates of anxiety disorders, bipolar disorder, and schizophrenia during this period.

The crude prevalence and DALY rate of IDID, conduct disorder, and autism spectrum disorders decreased in India from 1990 to 2017, with a stronger decrease in the high and middle SDI state groups than in the low SDI state group (figure 4). The crude prevalence of ADHD also decreased during this period, but the DALY rate did not change significantly. The age-standardised prevalence and DALY rate of IDID decreased significantly in India between 1990 and 2017, but no substantial change was observed for conduct disorder, autism spectrum disorders, and ADHD during this period. The crude prevalence and DALY rate of eating disorders increased in India from 1990 to 2017. This increase was higher in high and middle SDI state groups than in low SDI state group, with a similar trend for age-standardised prevalence and DALY rate.

### Risk factors

A small proportion of DALYs for depressive disorders and anxiety disorders could be attributed to known risk factors, with 6·9% of DALYs for depressive disorders due to childhood sexual abuse, 4·6% due to intimate partner violence, and 3·3% due to bullying victimisation (table 3). The proportion of DALYs for depressive disorder that could be attributed to childhood sexual abuse was significantly higher in females than in males. A large proportion of DALYs for IDID (62·8%) was attributable to lead exposure.

**Table 3:** Percentage contribution of major risk factors to mental disorders DALYs in India, 2017

	**Risk factor**	**Proportion of total DALYs attributable to each risk factor (95% uncertainty intervals)**		
		Both sexes	Males	Females
Depressive disorders	Bullying victimisation	3·3% (2·0–4·9)	3·8% (2·3–5·7)	3·0% (1·8–4·4)
Depressive disorders	Childhood sexual abuse	6·9% (5·7–8·2)	5·0% (4·1–6·2)	8·3% (6·6–10·3)
Depressive disorders	Intimate partner violence*	4·6% (3·3–6·2)	NA	8·0% (5·8–10·7)
Anxiety disorders	Bullying victimisation	7·0% (4·5–9·7)	7·7% (5·0–10·7)	6·4% (4·1–9·1)
Idiopathic developmental intellectual disability	Lead exposure	62·8% (32·2–81·2)	63·8% (32·9–82·1)	61·7% (31·7–80·4)

Data are percentage, with 95% uncertainty interval in parentheses. DALYs=disability-adjusted life-years. NA=not applicable.

* Intimate partner violence in GBD 2017 was modelled only for females.

## Discussion

This report reveals that in 2017, one among every seven people in India had a mental disorder, ranging from mild to severe. The proportional contribution of mental disorders to the total disease burden in India almost doubled from 1990 to 2017. Among the mental disorders that manifest predominantly during adulthood, the highest disease burden in India was caused by depressive and anxiety disorders, followed by schizophrenia and bipolar disorder. Among the mental disorders that have their onset predominantly during childhood and adolescence, the highest disease burden was caused by IDID, followed by conduct disorder and autism spectrum disorders.

The prevalence of mental disorders that manifest predominantly during adulthood was generally higher in the more developed southern states than in the less developed northern states, whereas the prevalence of mental disorders with onset predominantly in childhood and adolescence was generally higher in the less developed northern states than in the more developed southern states. The higher prevalence of depressive and anxiety disorders in southern states could be related to the higher levels of modernisation and urbanisation in these states and to many other factors that are not yet well understood.^25^, ^26^, ^27^, ^28^ We found a positive, but modest, relationship between depressive disorders and suicide death rates at the state level, with suicide death rates also being higher in the southern states than in the northern ones.^21^ This relationship has also been reported in previous studies.^29^, ^30^ It is also important to note that the high prevalence of depression among older adults has substantial implications because the population in India is ageing rapidly.

Sex differentials were observed in the distribution of mental disorders in India. The observed higher prevalence of depressive and anxiety disorders in females than in males has also been reported previously,^29^, ^31^ which could be related to gender discrimination, violence, sexual abuse, antenatal and postnatal stress, and adverse socio-cultural norms.^32^, ^33^, ^34^, ^35^ A significantly higher prevalence of eating disorders in females than in males has been reported elsewhere^36^ and, apart from genetic and biological factors, it is also probably linked with socio-cultural, media, and peer pressure to diet.^37^, ^38^ The higher prevalence of autism spectrum disorders and ADHD in males than in females has been reported previously.^39^ Past studies have suggested that genetic and hormonal factors could be behind the sex differentials in these disorders.^40^ The high prevalence of depressive disorders in older adults could be due to various factors, including chronic illness, social isolation and inadequate social support, and elder abuse.^41^, ^42^, ^43^, ^44^ This pattern of increasing prevalence with increasing age has not been reported in high-income countries, as also reported previously.^1^, ^45^

There was a varying degree of heterogeneity between the states of India in the DALY rates for individual mental disorders in 2017. On one hand, the population-level burden of depressive disorders, anxiety disorders, schizophrenia, bipolar disorder, and eating disorders increased in India between 1990 and 2017, with the increase being generally higher in the more developed states than in the less developed states. On the other hand, the population-level burden of mental disorders with onset in childhood and adolescence decreased by varying degrees for the individual disorders, and this decrease was generally smaller in the less developed states than in the more developed states. The increase in the burden of depressive disorders, anxiety disorders, schizophrenia, and bipolar disorder was driven by the ageing of the population because age-standardisation nullified this increase. The decrease in IDID could partly be attributed to the implementation of laws to reduce lead contamination in the country,^46^, ^47^ though this decrease was smaller in the less developed states than in the more developed ones. The increase in the burden of eating disorders could be related to several factors, including increasing exposure in India to global body-image trends.^37^, ^38^

India launched the National Mental Health Programme in 1982, which was relaunched in 1996 as the District Mental Health Programme.^48^ The National Mental Health Policy was introduced in 2014,^5^ and a rights-based Mental Healthcare Act in 2017, which replaced the Mental Healthcare Act of 1987.^49^ The child health programme under the National Health Mission and the National Adolescent Health Programme include components to address the mental health of children and adolescents.^50^, ^51^ The Ayushman Bharat (Healthy India) initiative launched in 2018 aims to provide comprehensive primary health care and health insurance coverage for non-communicable diseases including mental disorders, which could contribute to reducing the adverse effect of mental disorders at the population level.^52^, ^53^

Despite these efforts by the government, poor implementation of mental health services in India has been documented, with a high treatment gap for mental disorders, poor evidence-based treatment, and gender-differentials in treatment.^10^, ^12^, ^54^, ^55^, ^56^, ^57^, ^58^ A shortage of mental health personnel in India exists, with two mental health workers and 0·3 psychiatrists per 100 000 population, which is much lower than the global average.^59^ Additionally, the discriminatory attitude of health workers towards people with mental illness^60^, ^61^ and demand-side barriers such as low perceived need for care, paucity of knowledge of mental disorders, and stigma attached to mental disorders are challenges that need to be addressed.^61^, ^62^, ^63^, ^64^ An integrated approach to detect, treat, and manage patient needs related to mental and physical health is urgently needed in India because people with mental disorders die prematurely and have excess disability, though substantially more work is needed for it to be implemented on a large-scale.^61^, ^64^, ^65^, ^66^ Task-sharing with non-specialists and appropriate training of community health workers can improve mental health service provision.^67^, ^68^ Importantly, the positive association of depressive disorders and schizophrenia with suicide deaths, especially for females, needs urgent attention in primary care for suicide prevention, because Indian women have double the global suicide death rate.^21^ Furthermore, telemedicine to provide mental health services in remote and inaccessible areas, internet-based and telephone-based helplines, and mental health mobile apps can reduce the burden on existing mental health services.^69^, ^70^, ^71^

Communities and families have an important role in addressing mental health by reducing stigma and discrimination, raising awareness, and promoting inclusion.^72^, ^73^ Community-based programmes have the potential to reduce the treatment gap for mental disorders in India.^63^, ^74^, ^75^ School-based mental health programmes can help improve mental health in children.^76^ Yoga, a traditional Indian practice, is also suggested to be potentially beneficial for depressive disorders.^77^

The general limitations of the GBD methods, and those for estimation of mental disorders, have been discussed previously.^3^, ^17^, ^18^, ^19^, ^20^ A major limitation of this study is that the population-level data on the prevalence of many mental disorders are scant across the states of India, which might have introduced unknown biases in our estimates. Importantly, the GBD burden estimation for mental disorders relies on severity distributions primarily from high-income countries, which might not reflect the distribution in India. Because there is a paucity of research in India on risk factors for mental disorders, only well established risk factors from global data that could meet the strict risk-cause association criteria were included in the analysis. To address these limitations when data are scarce for a particular variable, GBD uses covariates and other techniques that borrow strength over space and time to arrive at the best possible estimates. We have presented the best possible estimates that could be modelled with the available data, which could be improved as more population-level data become available for mental disorders in India. It is also important to note that the total burden of mental disorders in this report is likely to be an underestimate, because the methods used do not fully include the contribution of mental disorders to mortality and morbidity from associated physical causes.^29^, ^61^, ^64^, ^78^ The strengths of this study include the use of data sources in India that could be accessed to estimate the trends and patterns of mental disorders in every state of India, the use of the standardised GBD methods for comparison across locations and years, and the inclusion of a comprehensive effort that benefitted from inputs from a network of leading experts in India.

In conclusion, mental disorders adversely affect a large proportion of Indians. Given the poor coverage of mental health services, the lack of awareness, and the stigma attached to mental disorders in the country, India needs to invest heavily in mental health services to facilitate prevention where possible and to provide affordable treatment, care, and rehabilitation, as well as to attempt integration of mental and physical health services. The state-specific data trends in this report can be useful for mental health policies and programme planning at the individual state level and for India as a whole.
