# Supplementary material for: The burden of mental disorders across the states of India: the Global Burden of Disease Study 1990–2017
Source: Lancet Psychiatry. 2020 Feb;7(2):148–61. doi: 10.1016/S2215-0366(19)30475-4 (PMC7029418; doi:10.1016/S2215-0366(19)30475-4)
Supplement: Supplementary appendix [file mmc1.pdf]

THE LANCET
Psychiatry

Supplementary appendix

This appendix formed part of the original submission and has been peer reviewed. We post it as supplied by the authors.

Supplement to: India State-Level Disease Burden Initiative Mental Disorders Collaborators. The burden of mental disorders across the states of India: the Global Burden of Disease Study 1990–2017. *Lancet Psychiatry* 2019; published online Dec 23. https://doi.org/10.1016/S2215-0366(19)30475-4.

The burden of mental disorders across the states of India:

the Global Burden of Disease Study 1990-2017

The India State-Level Disease Burden Initiative Mental Disorders Collaborators

Web Appendix

Correspondence to: Prof. Lalit Dandona, lalit.dandona@icmr.gov.in

Table of contents

1. GBD 2017 mental disorders burden estimation methods 3
2. GBD 2017 India data inputs for mental disorders burden, risk factors and covariates 40
3. Grouping of the states of India based on SDI, 2017 47
4. Prevalence of mental disorders in the states of India, 2017 48
5. Relationship of the prevalence of depressive disorders, anxiety disorders, schizophrenia, and bipolar disorder with suicide death rate in states of India, 2017 49
6. Age-specific prevalence of mental disorders in India, by sex, 2017 50

1. GBD 2017 mental disorders burden estimation methods

The material presented here is adapted from the following sources:

- GBD 2017 Disease and Injury Incidence and Prevalence Collaborators. Global, regional, and national incidence, prevalence, and years lived with disability for 354 diseases and injuries for 195 countries and territories, 1990–2017: a systematic analysis for the Global Burden of Disease Study 2017. *Lancet* 2018; 392: 1789–858.GBD 2017
- Causes of Death Collaborators. Global, regional, and national age-sex-specific mortality for 282 causes of death in 195 countries and territories, 1980–2017: a systematic analysis for the Global Burden of Disease Study 2017. *Lancet* 2018; 392: 1736–88.
- GBD 2017 Risk Factor Collaborators. Global, regional, and national comparative risk assessment of 84 behavioural, environmental and occupational, and metabolic risks or clusters of risks for 195 countries and territories, 1990–2017: a systematic analysis for the Global Burden of Disease Study 2017. *Lancet* 2018; 392: 1923–45.

The GBD cause list is organised hierarchically into four levels. At each level of the hierarchy, the set of causes is mutually exclusive and collectively exhaustive. Levels 1 and 2 represent general groupings. The broad group “mental disorders’ is at level 2 under the level 1 group “non-communicable diseases”. Level 3 includes seven diseases which are: schizophrenia, depressive disorders, bipolar disorders, anxiety disorders, eating disorders, autism spectrum disorders, attention-deficit/hyperactivity disorder, conduct disorder, idiopathic developmental intellectual disability, and other mental disorders. Level 4 includes four groups, under the parent level 3 “depressive disorders” group: major depressive disorder and dysthymia and level 3 “eating disorders” group: anorexia nervosa, and bulimia nervosa.

A. GBD case definitions of mental disorders

The GBD case definitions and diagnostic criteria for the mental disorders are presented below:

Major depressive disorder

Major depressive disorder (MDD) is an episodic mood disorder involving the experience of one or more major depressive episode(s). Included in GBD disease modelling were cases meeting diagnostic criteria for MDD according to the Diagnostic and Statistical Manual of Mental Disorders (DSM) or the equivalent diagnosis of recurrent depression in the International Classification of Diseases (ICD).

According to the Diagnostic and Statistical Manual of Mental Disorders fourth edition, text revision (DSM-IV-TR) criteria, MDD involves the presence of at least one major depressive episode, which is the experience of depressed mood almost all day, every day, for at least two weeks. Mood must represent a change from the baseline and impaired functioning must be observed across social, occupational, and educational domains. Additionally, a total of five out of nine criteria must be met to make a diagnosis and at least one of the five criteria should either be: depressed mood for most of every day; or loss of interest in nearly all activities for most of every day.

The other seven criteria are:

- Change in eating, appetite, or weight
- Excessive sleeping or insomnia
- Agitated or slow motor activity
- Fatigue
- Feeling worthless or inappropriately guilty
- Trouble concentrating
- Repeated thoughts about death

Dysthymia

Dysthymia is a mood disorder consisting of chronic depression, demonstrating less severe but longer lasting symptoms than major depressive disorder. Included in GBD disease modelling were cases meeting diagnostic criteria for dysthymia according to the Diagnostic and Statistical Manual of Mental Disorders (DSM, or the equivalent diagnosis in the International Classification of Diseases (ICD).

According to DSM-IV TR criteria, dysthymia involves the experience of chronically depressed mood for most of the day, most days that not, for at least two years (or at least one year in children and adolescents). During this period, at least two of the following symptoms must also be experienced:

- Poor appetite or overeating
- Insomnia or hypersomnia
- Low energy or fatigue
- Low self-esteem
- Poor concentration or indecisiveness
- Feelings of hopelessness

Anxiety disorders

Anxiety disorders are characterised by experiences of intense of fear and distress, typically in combination with other physiological symptoms. GBD aimed to capture all cases of anxiety disorders reaching diagnostic threshold defined by the Diagnostic and Statistical Manual of Mental Disorders (DSM) or the WHO International Classification of Diseases (ICD-10. Included disorders are listed below:

- Panic disorder
- Agoraphobia
- Specific phobia
- Social phobia
- Obsessive-compulsive disorder
- Post-traumatic stress disorder
- Acute stress disorder
- Generalised anxiety disorder
- Separation anxiety disorder
- Anxiety disorder not otherwise specified

As specific anxiety disorders frequently co-occur, anxiety disorders were modelled as a single cause for “any” anxiety disorder in GBD 2017 to avoid the double-counting of individuals meeting criteria for more than one anxiety disorder. Epidemiological estimates reporting an outcome for “any” or “total” anxiety disorders were included in analyses.

Idiopathic developmental intellectual disability

Developmental intellectual disability is a condition of below-average intelligence or mental ability. Consistent with the American Association on Intellectual and Developmental Disabilities, GBD define developmental intellectual disability as a condition originating before age 18. GBD modelled the severities shown in the below table, as measured by score on intelligence quotient (IQ) tests, which are standardised to have a mean of 100.

Developmental intellectual disability severity definitions

Severity of intellectual disability	IQ score
Profound	0-19
Severe	20-34
Moderate	35-49
Mild	50-69
Borderline	70-85

Idiopathic intellectual developmental disability is defined by exclusion. The sum of intellectual disability as a sequela from underlying causes estimated in GBD was subtracted from the overall ‘envelope’ of all intellectual disability based on IQ testing data to arrive at the remnant estimate of idiopathic developmental intellectual disability.

Schizophrenia

Schizophrenia is a chronic psychotic disorder which involves the experience of positive symptoms (e.g. delusions, hallucinations, thought disorder) and negative symptoms (e.g. flat affect, loss of interest, and emotional withdrawal). Diagnostic criteria are:

Two (or more) of the following, each present for a significant portion of time during a one-month period (or less if successfully treated):

- Delusions
- Hallucinations
- Disorganised speech
- Grossly disorganised or catatonic behaviour
- Negative symptoms
- Social/occupational dysfunction
- Continuous signs of the disturbance persist for at least 6 months
- Exclusions must be met for schizoaffective and mood disorders, substance and general medical conditions, and a relationship to a pervasive development disorder

Bipolar disorder

Bipolar disorder is a chronic mood disorder with little or no complete remission. Included in GBD disease modelling were cases meeting diagnostic criteria for bipolar disorder according to the Diagnostic and Statistical Manual of Mental Disorders (DSM), or the equivalent diagnosis in the International Classification of Diseases (ICD). A diagnosis of bipolar disorder involves the experience of one or more manic or hypomanic episode(s), which can be accompanied by a major depressive episode.

According to DSM-IV-TR, a manic episode involves the experience of elevated, expansive, or irritable mood lasting for at least one week. During this period, at least three (or four if mood is only irritable) of the following symptoms must also be experienced:

- Inflated self-esteem or grandiosity
- Decreased need for sleep
- More talkative
- Flight of ideas or experience that thoughts are racing
- Distractibility
- Increase in goal-directed activity
- Excessive involvement in pleasurable activities with high potential for painful consequences

A hypomanic episode involves the experience of elevated, expansive, or irritable mood lasting for at least four days. During this period, at least three (or four if mood is only irritable) of the symptoms previously listed for a manic episode must also be experienced.

A major depressive episode involves the experience of depressed mood almost all day, every day, for at least two weeks. A total of five of nine criteria must be met to make a diagnosis and at least one of the five criteria should either be: depressed mood for most of every day; or loss of interest in nearly all activities for most of every day.

The other seven criteria are:

- Change in eating, appetite, or weight
- Excessive sleeping or insomnia
- Agitated or slow motor activity
- Fatigue
- Feeling worthless or inappropriately guilty
- Trouble concentrating
- Repeated thoughts about death

Different subtypes of bipolar disorder can be diagnosed depending on the combination of symptoms experienced. Bipolar I is characterised by at least one manic episode, which can also alternate with a major depressive episode. Bipolar II is characterised by hypomanic episodes alternating with major depressive episodes. Cyclothymia is characterised by subsyndromal hypomanic and major depressive episodes. Bipolar disorder not otherwise specified is characterised by clinically significant symptoms of bipolar disorder which do not meet criteria for the other diagnoses. In GBD 2017 burden for the entire spectrum of bipolar disorder

estimated simultaneously, rather than individually for each subtype of the disorder. At a minimum, epidemiological studies needed to report on bipolar I and bipolar II combined to be included in analyses.

Conduct disorder

Conduct disorder (CD) is an externalising behaviour disorder characterised by a pattern of antisocial behaviour that violates the basic rights of others or major age-appropriate societal norms. As per criteria set by DSM-IV-TR, diagnosis requires three or more of the following symptoms to be present in the past 12 months (with at least one present in the last six months) and cause significant impairment in functioning. Symptoms include:

- Aggression to people and animals
- Often bullies, threatens, or intimidates others
- Often initiates physical fights
- Has used a weapon that can cause serious physical harm to others (e.g. a bat, brick, broken bottle, knife, gun)
- Has been physically cruel to people
- Has been physically cruel to animals
- Has stolen while confronting a victim (e.g. mugging, purse snatching, extortion, armed robbery)
- Has forced someone into sexual activity
- Destruction of property
- Has deliberately engaged in fire setting with the intention of causing serious damage
- Has deliberately destroyed other's property (other than by fire setting)
- Deceitfulness or theft
- Has broken into someone else's house, building, or car
- Often lies to obtain goods or favours or to avoid obligations (i.e., cons others)
- Has stolen items of nontrivial value without confronting a victim (e.g. shoplifting, but without breaking and entering; forgery)
- Serious violations of rules
- Often stays out at night despite parental prohibitions, beginning before age 13 years
- Has run away from home overnight at least twice while living in parental or parental surrogate home (or once without returning for a lengthy period)
- Is often truant from school, beginning before age 13 years

CD is considered a disorder of childhood but can be diagnosed in adults who display such behaviours yet do not meet the criteria for antisocial personality disorder. However, there are almost no studies measuring adult CD as existing studies in this area tend to measure adult antisocial behaviour rather than adult CD. As such, only childhood CD (i.e., cases prior to 18 years of age) was modelled in GBD. Included in GBD were cases meeting diagnostic criteria according to DSM1 or the International Classification of Diseases (ICD).

Anorexia nervosa

According to the DSM IV- TR, anorexia nervosa (AN) is an eating disorder characterised by:

- Refusal to maintain body weight at or above a minimally normal weight for age and height (e.g. weight loss leading to maintenance of body weight less than 85% of that expected; or failure to make expected weight gain during period of growth, leading to body weight less than 85% of that expected).
- Intense fear of gaining weight or becoming fat, even though underweight (expanded to include any behaviour that interferes with weight gain in DSM-52).
- Disturbance in the way in which one's body weight or shape is experienced, undue influence of body weight or shape on self-evaluation, or denial of the seriousness of the current low body weight.
- In post-menarcheal females, amenorrhoea, i.e., the absence of at least three consecutive menstrual cycles (this criterion was removed in DSM-52).

Included in GBD were cases meeting diagnostic criteria according to DSM or the International Classification of Diseases (ICD.

Bulimia nervosa

According to the DSM- IV-TR, bulimia nervosa (BN) is an eating disorder characterised by:

- Recurrent episodes of binge eating. An episode of binge eating is characterised by both of the following:
    1. eating, in a discrete period of time (e.g. within any two-hour period), an amount of food that is definitely larger than most people would eat during a similar period of time and under similar circumstances
    2. sense of lack of control over eating during the episode (e.g. a feeling that one cannot stop eating or control what or how much one is eating).
- Recurrent inappropriate compensatory behaviour in order to prevent weight gain, such as self-induced vomiting; misuse of laxatives, diuretics, enemas, or other medications; fasting; or excessive exercise.
- The binge eating and inappropriate compensatory behaviour both occur, on average, at least twice a week for three months (changed to once a week for three months in DSM-5).
- Self-evaluation is unduly influenced by body shape and weight.
- The disturbance does not occur exclusively during episodes of anorexia nervosa.

Included in GBD were cases meeting diagnostic criteria according to DSM or the International Classification of Diseases (ICD.

Autism spectrum disorders

Autism spectrum disorders (ASD; also known as pervasive developmental disorder) is a neurodevelopmental disorder with onset occurring in early childhood. It is characterised by pervasive impairment in several areas of development, including social interaction and communication skills, along with restricted and repetitive patterns of behaviours and/or interests.

ASD was an umbrella for five sub-disorders according to the Diagnostic and Statistical Manual of Mental Disorders fourth edition, text revision (DSM-IV-TR: Autistic disorder (299.00), Pervasive Developmental disorder, not otherwise specified (299.8), Rett's disorder (299.80), Childhood Disintegrative Disorder (299.10). ASD is still an umbrella for eight sub-disorders according to the International Statistical Classification of Diseases and Related Health Problems 10th Revision (ICD10). A diagnosis of ASD according to the DSM-5 requires the following criteria to be met:

Persistent deficits in social communication and social interaction across multiple contexts, as manifested by all of the following, currently or by history:

- Deficits in social-emotional reciprocity, ranging, for example, from abnormal social approach and failure of normal back-and-forth conversation to reduced sharing of interests, emotions, or affect to failure to initiate or respond to social interactions.
- Deficits in nonverbal communicative behaviours used for social interaction, ranging, for example, from poorly integrated verbal and nonverbal communication to abnormalities in eye contact and body language or deficits in understanding and use of gestures to a total lack of facial expressions and nonverbal communication.
- Deficits in developing, maintaining, and understanding relationships, ranging, for example, from difficulties adjusting behaviour to suit various social contexts to difficulties in sharing imaginative play or in making friends to absence of interest in peers.

Restricted, repetitive patterns of behaviour, interests, or activities, as manifested by at least two of the following, currently or by history:

- Stereotyped or repetitive motor movements, use of objects, or speech (e.g. simple motor stereotypies, lining up toys or flipping objects, echolalia, idiosyncratic phrases).
- Insistence on sameness, inflexible adherence to routines, or ritualised patterns of verbal or nonverbal behaviour (e.g. extreme distress at small changes, difficulties with transitions, rigid thinking patterns, greeting rituals, need to take same route or eat same food every day).
- Highly restricted, fixated interests that are abnormal in intensity or focus (e.g. strong attachment to or preoccupation with unusual objects, excessively circumscribed or perseverative interests).
- Hyper- or hypo reactivity to sensory input or unusual interest in sensory aspects of the environment (e.g. apparent indifference to pain/temperature, adverse response to specific sounds or textures, excessive smelling or touching of objects, visual fascination with lights or movement).

The symptoms must be present in the early developmental period, cause clinically significant impairment, and not be better explained by intellectual impairment or global developmental delay.

Attention-deficit/hyperactivity disorder

Attention-deficit/hyperactivity disorder (ADHD) is an externalising behaviour disorder characterised by persistent inattention and/or hyperactivity-impulsivity. As per criteria set by the DSM-IV-TR, diagnosis requires six or more symptoms of inattention or hyperactivity-impulsivity to have persisted for at least six months in two or more settings causing significant impairment to functioning, with at least some impairing symptoms being present prior to 7 years of age (12 years of age in DSM-5). Recognised symptoms include:

Inattention

- Often fails to give close attention to details or makes careless mistakes in schoolwork, work, or other activities
- Often has difficulty sustaining attention in tasks or play activities
- Often does not seem to listen when spoken to directly
- Often does not follow through on instructions and fails to finish schoolwork, chores, or duties in the workplace (not due to oppositional behaviour or failure to understand instructions)
- Often has difficulty organising tasks and activities
- Often avoids, dislikes, or is reluctant to engage in tasks that require sustained mental effort (such as schoolwork or homework)
- Often loses things necessary for tasks or activities (e.g. toys, school assignments, pencils, books, or tools)
- Is often easily distracted by extraneous stimuli
- Is often forgetful in daily activities

Hyperactivity

- Often fidgets with hands or feet or squirms in seat
- Often leaves seat in classroom or in other situations in which remaining seated is expected
- Often runs about or climbs excessively in situations in which it is inappropriate (in adolescents or adults, may be limited to subjective feelings of restlessness)
- Often has difficulty playing or engaging in leisure activities quietly
- Is often on the go or often acts as if driven by a motor
- Often talks excessively

Impulsivity

- Often blurts out answers before questions have been completed
- Often has difficulty awaiting turn
- Often interrupts or intrudes on others (e.g. butts into conversations or games)

Included in GBD were cases meeting diagnostic criteria according to DSM or the International Classification of Diseases (ICD) (called hyperkinetic disorder in ICD).

Other mental disorders

In addition to the individual mental disorders for which GBD estimated burden, the non-fatal burden attributable to a residual cause of other mental disorders were also estimated. This is made up of an aggregate group of personality disorders. Personality disorders are characterised by pervasive, inflexible and maladaptive patterns of behaviour and inner experience which are markedly different from what is considered to be acceptable in the individual’s culture. These disorders tend to be chronic and are associated with significant distress or disability. Included in GBD 2017 were cases meeting diagnostic criteria for personality disorders according to the Diagnostic and Statistical Manual of Mental Disorders (DSM, or the equivalent diagnosis in the International Classification of Diseases (ICD). The aggregated group of personality disorders used in GBD 2017 captured any of the following:

- Paranoid personality disorder
- Schizoid personality disorder
- Schizotypal personality disorder
- Antisocial personality disorder
- Borderline personality disorder

- Histrionic personality disorder
- Narcissistic personality disorder
- Avoidant personality disorder
- Dependent personality disorder
- Obsessive-compulsive personality disorder
- Personality disorder not otherwise specified

B. List of ICD codes mapped to the GBD mental disorders list

The codes used by GBD 2017 from the 9th and 10th revisions of the International Statistical Classification of Diseases and related health problems (ICD) for mental disorders are listed below:

Cause	ICD 10	ICD 9
Depressive disorder	F32-F33.9, F34.1	296.2-296.36, 300.4, 311-311.9
Major depressive disorder	F32-F33.9	296.2-296.36, 311-311.9
Dysthymia	F34.1	300.4
Anxiety disorders	F40-F44.9, F93-F93.2	300-300.3, 308-309.9
Idiopathic developmental intellectual disability	F70-F79.9, Z81.0	317-319.9, V18.4
Schizophrenia	F20-F20.9, F25-F25.9	295-295.35, 295.5-295.8
Bipolar disorder	F30-F31.9, F34.0	296-296.16, 296.4-296.81
Conduct disorder	F91-F92.9	312-312.9
Eating disorders	F50-F50.9	307.1, 307.5-307.59
Anorexia nervosa	F50.0-F50.1	307.1
Bulimia nervosa	F50.2-F50.5	307.51, 307.54
Autism spectrum disorders	F84-F84.9	299-299.91
Attention-deficit/hyperactivity disorder	F90-F90.9	314-314.9
Other mental disorders	F04-F06.1, F06.3-F07.0, F08-F09.9, F21-F24, F26-F29.9, F34, F34.8, F34.9, F38-F39, F45-F49, F51-F52.9, F55, F55.8, F56-F69.0, F80-F83, F85-F89.0, F93.3-F99.0, G47-G47.29, G47.4-G47.9, R40-R40.4, R45-R55.0, Z03.2, Z04.6-Z04.72, Z13.4, Z64, Z81, Z81.8, Z86.5 Z86.59	293-294, 295.4-295.45, 295.80-295.95, 296.82-298.9, 300.5-302.9, 306-307.0, 307.2-307.49, 307.6-307.7, 307.9, 310-310.1, 313-313.9, 316 316.9, 327-327.19, 327.3-327.8, 347-347.9, 780-780.2, 780.93, 780.97, 797-797.9, 799.2-799.29, V11.0-V11.2, V11.4-V12.0, V17-V17.0.

C. GBD data and analysis framework

The overview of data inputs and analysis framework for GBD is shown in the following flowchart:

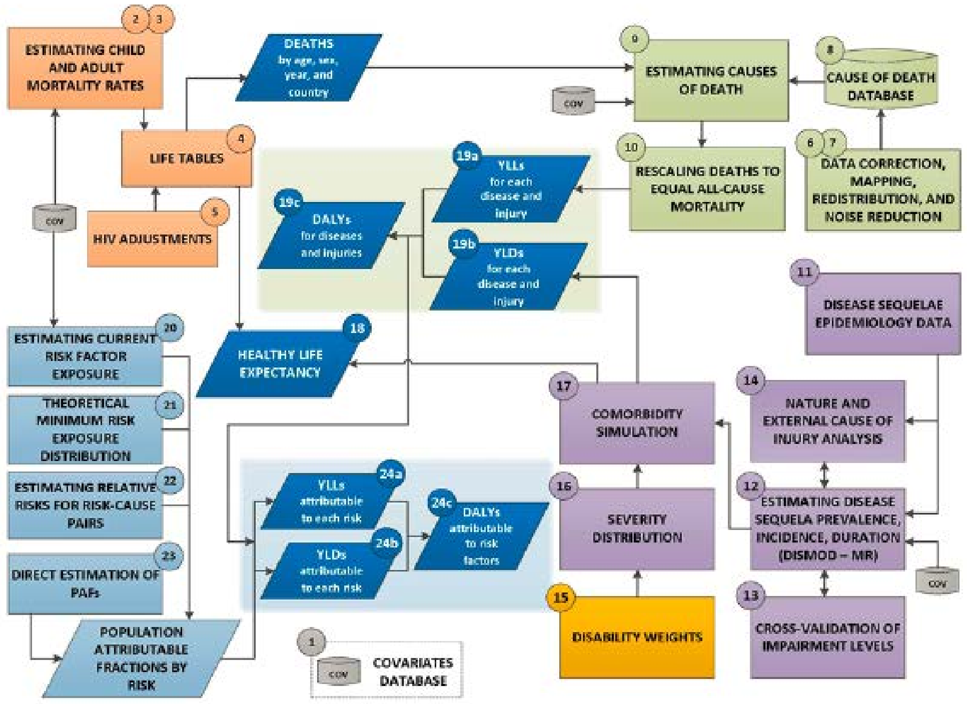

YLLs is years of life lost. YLDs is years lived with disability. DALYs is disability-adjusted life-years. PAFs is population attributable fractions.
Rectangular boxes represent analytical steps, cylinders represent databases, and parallelograms represent intermediate and final results.

The flowchart above illustrates the flow of the key components of the GBD estimation process, including:

1. Incorporation of appropriate covariates (step 1)
2. All-cause mortality estimation (steps 2-5): the data come from sources such as censuses, surveys and vital registrations. The all-cause mortality estimation process (steps 2-4) can be divided into four distinct but interconnected areas: child mortality and adult mortality between ages 15 and 60, estimation of a complete set of age-specific death rates, estimation of HIV mortality and final estimates of age-specific mortality including HIV and fatal discontinuities (also known as mortality shocks) (step 5).
3. Cause of death estimation (steps 6-9): cause of death data are derived from vital registrations, verbal autopsy studies, mortality surveillance and, for selected causes, police records, crime reports and data collection systems for deaths due to conflict and natural disasters (step 7). Extensive data corrections and redistributions of ill-defined causes are made to correct for measurement bias between data sources. Cause of death ensemble modelling (CODEm), an ensemble model, is a systematized approach to analysing cause of death data for all but a few causes (step 9). CODEm explores a wide range of modelling approaches and varying predictive covariates to find an ensemble of best-performing models based on statistical tests. To do so, 30% of the data are withheld from each model and the model fit is evaluated by how well it covers the data that were left out. By repeating this process many times over the best performing models are selected. As all results in GBD are estimated 1,000 times over to propagate all sources of uncertainty, among the 1,000 runs we end up with an ensemble of up to 100 or more different types of models and covariates that are selected.
4. Rescaling deaths to equal all-cause mortality (step 10): as all these estimates are made separately for each disease and injury, the sum of these could exceed or fall below the all-cause mortality estimated from the demographic analyses of steps 2 to 5. Therefore, all deaths by age, sex, geography, year and cause to match the all-cause death estimates (this process is called CoDcorrect).
5. Estimation of disease sequelae prevalence, incidence, and duration (steps 11-12): population surveys, cohort studies, administrative records of hospitalisations and other health service encounters, disease registries, notifications, surveillance systems are the main data sources for non-fatal estimation (step 11). Extensive corrections of data to deal with measurement bias arising from study design or case definitions are applied. DisMod-MR 2.1 is the main analytical tool for non-fatal estimation (step 12). It is a Bayesian meta-regression software program that uses a lognormal model. The meta-regression component allows corrections for known sources of measurement error. Its core function is to make estimates of prevalence and incidence of disease that are consistent with data on mortality risk and remission (defined in GBD as the 'cure rate'). For a select number of causes that do not fit well in the three state model (alive without disease, prevalent case of disease and death) of DisMod-MR 2.1, was used as alternative modelling strategies.
6. Cross-validation of impairment levels (step 13): for a number of impairments in GBD terminology, such as anaemia, heart failure, hearing and vision loss, we first estimate the total levels of prevalence and incidence and then ensured that all sequelae of diseases that lead to this impairment add up to the total.
7. Analysis of the nature and external cause of injury is done separately (step 14). Assignment of severity distributions for the main disabling conditions (step 15): in GBD terminology sequelae are the disabling consequences for which we make estimates. All sequelae are defined to be mutually exclusive and collectively exhaustive. Many diseases have sequelae with a gradation by severity such as mild, moderate and severe dementia. Often the epidemiological data on severity distribution is sparse. Therefore, at first model the epidemiology of all cases of disease and then apply a severity distribution from the sparser data.
8. Assignment of disability weights for health states (step 16): each sequela is matched with a health state or combination of health states for which we have a disability quantifies the relative severity.
9. Disability weights were derived from population and internet surveys of over 60,000 respondents answering pair-wise comparison question of random combinations of health states. Each pair of health states was described with brief lay descriptions highlighting the main symptoms and impairments. Respondents were asked to nominate the 'healthier' of each presented pair. Analytical methods exist to formalise the intuition that if the majority of respondents nominate one health state in a pair as the healthier these lie farther apart on a severity scale than pairs assigned similar proportions as the healthier. In order to anchor estimates on a 0-1 scale of severity, a subset of respondents was asked additional population health equivalence questions on a selection of health states. These questions ask for a choice of the greater amount of health produce by two health programs; one that prevented sudden death in 1,000 persons and another that prevented the onset of a GBD health state for the rest of 2,000, 5,000 or 10,000 persons' lives.

10. Simulation of comorbidity (step 17): the last step of non-fatal estimation is a microsimulation ('COMO') to deal with comorbidity. For every age, sex, geography and year, 40,000 hypothetical persons are generated who have none, one or more of the GBD sequelae. In those with multiple sequelae their combined level of disability is estimated multiplicatively. That means we assume the disability from having two health states is less than the sum of the corresponding disability weights. This avoids assigning disability greater than one to any individual which would indicate that person is worse off than being dead.
11. Estimation of healthy life expectancy (step 18): health life expectancy is estimated from the life tables generated in step 4 and the all-cause YLD rates from step 19b.
12. Computation of YLLs, YLDs, and DALYs from diseases and injuries with uncertainty (steps 19a-19c): YLLs (step 19a) are estimated as the product of counts of death by ages, sex, geography, year and cause and a normative life expectancy at the age of the death. The GBD standard life expectancy used as this norm is a compilation of the lowest observed mortality rates by age in all mortality data collections of populations greater than 5 million. The standard life table reflects a life expectancy at birth of 86.59 years. YLDs are the output from COMO (step 19b). DALYs are the simple addition of YLLs and YLDs (step 19c).
13. Risk factor estimation (steps 20-24): GBD 2017 also makes estimates for individual and combined risk factors. This involves estimation of risk factor exposure (step 20); the formulation of a minimum level of exposure to each risk that is associated with the least amount of health loss (step 21); derivation of relative risks of disease outcomes for each pair of a risk factor and a disease or injury for which there is judged to be sufficient evidence of a causal relationship (step 22); and the estimation of population attributable fractions of disease caused by each risk factor. For a few risk-outcome pairs it is hard to define exposure and a corresponding risk while directly observed proportions of disease are available, such as for the proportion of HIV/AIDS due to unsafe sex or injecting drug use (step 23). For combinations of risks how much of the risk is mediated through other risks (step 24) was assessed. For instance, all of the effect of high salt intake is mediated through elevated blood pressure and part of the risk of increased body mass index is through elevated blood pressure, cholesterol or fasting plasma glucose.
14. Computation of YLLs, YLDs, and DALYs attributable to risk factors (steps 25a-c): YLLs, YLDs and DALYs attributable to each risk factor are generated by multiplying population attributable fractions with disease estimates (steps 25a-c).

D. Mental disorders morbidity estimation

The major data inputs used for estimating prevalence of mental disorders in India are population-based surveys, including World Mental Health Survey 2003 that included India and National Mental Health Survey 2015-16, and other published studies. Mental disorders morbidity was modelled using the DisMod-MR 2.1 platform. Morbidity estimation and modelling methods for the mental disorders are presented in this paper are described in detail below.

D.1. Major depressive disorder

The steps in the estimation of non-fatal major depressive disorder (MDD) burden or morbidity are shown in the following flowchart:

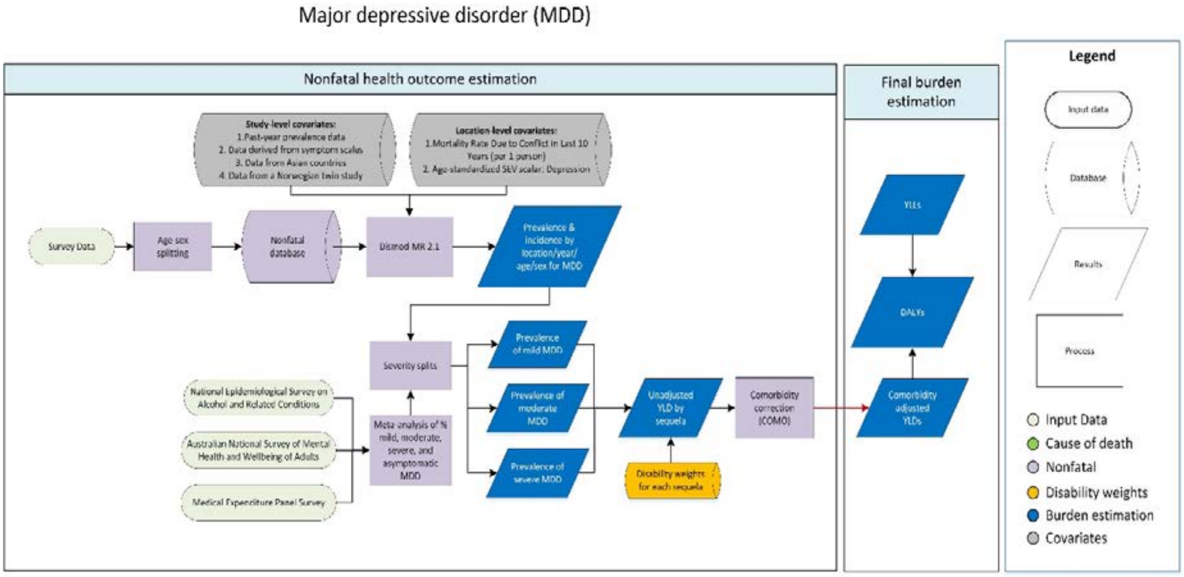

Data

Prevalence estimates of MDD were split by age and sex where possible. If studies reported prevalence for broad age groups by sex (e.g. prevalence in 15 to 65 year old males and females separately), and also by specific age groups but for both sexes combined (e.g. prevalence in 15 to 30 year old, then in 31 to 65 year old, for males and females combined); then age-specific estimates were split by sex using the reported sex ratio and bounds of uncertainty. If studies reported estimates across age groups spanning 20 years or more then the data were split into five-year age groups using the prevalence age pattern estimated by DisMod-MR 2.1.

Attributable suicide estimates

As MDD is an established risk factor for suicide, data on excess mortality with estimated suicide rates (by age, sex, year, and location) attributable to MDD were also added in the morbidity estimation process. The excess mortality data were estimated using GBD's comparative risk assessment methodology, where the current health status was compared with a theoretical-minimum-risk exposure defined as the counterfactual status of the absence of MDD in the population. Population attributable fractions (PAFs) were estimated using this established formula:

$$PAF = \frac{p\ (RR-1)}{p\ (RR-1)+1}$$

P referred to the exposure distribution, which in this case was the DisMod-MR 2.1 prevalence rates of MDD by age, sex, location and year. RR referred to the pooled relative-risk of suicide due to MDD obtained from an existing systematic review and meta-analysis. Age, sex, year, and location-specific PAFs were multiplied by their corresponding GBD suicide rate to estimate the proportion of suicide cases attributable to MDD. These were entered as cause-specific mortality rates in the epidemiological model for MDD.

Severity splits

The basis of the GBD disability weight survey assessments are lay descriptions of sequelae highlighting major functional consequences and symptoms. The lay descriptions and disability weights for MDD severity levels are shown below:

Severity level	Lay description	Disability weight (95% CI)
Mild	Feels persistent sadness and has lost interest in usual activities. The person sometimes sleeps badly, feels tired, or has trouble concentrating but still manages to function in daily life with extra effort.	0.145 (0.099 0.209)
Moderate	Has constant sadness and has lost interest in usual activities. The person has some difficulty in daily life, sleeps badly, has trouble concentrating, and sometimes thinks about harming himself (or herself).	0.396 (0.267 0.531)
Severe	Has overwhelming, constant sadness and cannot function in daily life. The person sometimes loses touch with reality and wants to harm or kill himself (or herself).	0.658 (0.477 0.807)

Modelling strategy

Morbidity from MDD was modelled using the DisMod-MR 2.1 platform. Data across all epidemiological parameters were initially included in the modelling process. However, few incidence data points available typically excluded cases of MDD at baseline, new major depressive episodes in people with previous episodes were not counted and incidence was underestimated. For this reason, all raw incidence data were excluded in the final model and instead allowed DisMod-MR 2.1 to calculate incidence based on data from other parameters.

Minimum age of onset for MDD was set after 3 years of age and validated the same with expert feedback and existing literature. An average remission rate for a major depressive episode of 1.4 (1.3-1.6) was used. This was derived from the four longitudinal studies fitting a lognormal curve with least squared differences to data on the proportion of incident cases still fulfilling the case definition for major depression at intervals over a one-year period. As data were only available for a follow-up of one year, a decision had to be made about the maximum allowable duration of an episode. Setting this at 40 years, the average duration implied by the lognormal fit was 0.65 (0.59-0.70) of a year. Study-level covariates were used to accommodate between-study variability in the raw prevalence data. A past year recall covariate adjusted all data points derived from past year prevalence toward the level they would have been if the study had captured point/past-month prevalence. The latter prevalence period is less affected by recall bias. A symptom scale covariate adjusted all data points derived using a symptom scale toward the level they would have been if the scale had strictly adhered to DSM or ICD thresholds for MDD.

Location-level covariates were also included in the MDD model. For each GBD location, a covariate identifying the mean mortality rate in the previous ten years due to war and terrorism informed the estimation of prevalence given existing evidence to show a positive association between conflict status and the prevalence of MDD. An age-standardised SEV scalar was also included. This made use of the fraction of MDD burden caused by its relevant risk factors combined to inform the estimation of prevalence. Intimate partner violence and childhood sexual violence are the two established risk factors of MDD for which attributable burden is estimated in GBD studies. Betas and exponentiated values (which can be interpreted as an odds ratio) for each study and country-level covariate are shown in the table below:

Study/country covariate	Parameter	Beta	Exponentiated beta
Asian data points	Prevalence	-0.42 (-0.48 to -0.37)	0.66 (0.62 0.69)
Past year recall	Prevalence	0.67 (0.63 0.72)	1.96 (1.88 2.05)
Symptom scale	Prevalence	1.09 (1.04 1.15)	2.98 (2.82 3.15)
School survey	Prevalence	0.27 (0.17 0.38)	1.32 (1.18 1.46)
World health survey	Prevalence	0.84 (0.77 0.92)	2.31 (2.15 2.51)
Mean war mortality rate in the previous ten years	Prevalence	0.49 (0.022 0.97)	1.63 (1.02 2.65)
Age-standardised SEV scalar: Depression	Prevalence	1.15 (0.93 1.25)	3.16 (2.53 3.48)

D.2. Dysthymia

The steps in the estimation of non-fatal dysthymia burden or morbidity are shown in the following flowchart:

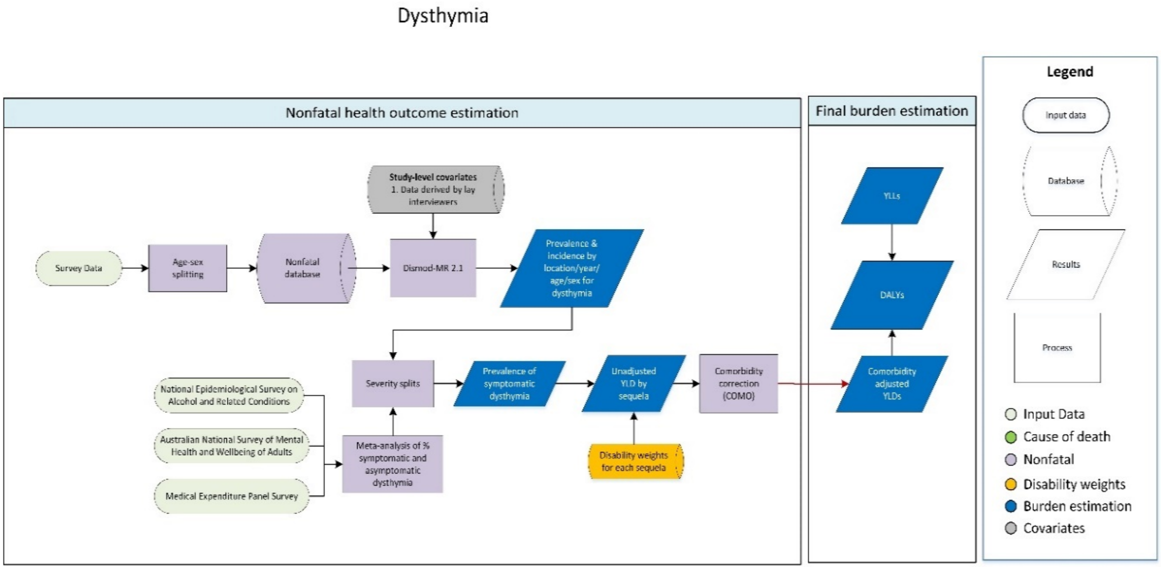

Data

The estimated prevalence of dysthymia was split by age and sex where possible. First, if studies reported prevalence for broad age groups by sex (e.g. prevalence in 15 to 65 year old males and females separately), and also by specific age groups but for both sexes combined (e.g. prevalence in 15 to 30 year old, then in 31 to 65 year old, for males and females combined), age specific estimates were split by sex using the reported sex ratio and bounds of uncertainty. Second, where studies reported estimates across age groups spanning 20 years or more, these were split into five year age groups using the prevalence age pattern estimated by DisMod-MR 2.1.

Severity splits

The basis of the GBD disability weight survey assessments are lay descriptions of sequelae highlighting major functional consequences and symptoms. The lay description and disability weight for a symptomatic state of dysthymia are shown below. Given the milder and more stable presentation of dysthymia, it was assigned the same disability weight as that for mild major depressive disorder.

**Severity level**	**Lay description**	**Disability weight (95% CI)**
Symptomatic dysthymia	Feels persistent sadness and has lost interest in usual activities. The person sometimes sleeps badly, feels tired, or has trouble concentrating but still manages to function in daily life with extra effort.	0.145 (0.099 0.209)

Modelling strategy

The DisMod-MR 2.1 model was used for the estimation of non-fatal estimation of dysthymia. Data across all epidemiological parameters were initially included in the modelling process. The studies with incidence data have reported very low estimates of dysthymia, whereas prevalence data was relatively high. As prevalence studies contributed much greater world coverage than incidence studies, the incidence data were excluded from the modelling. The minimum age of onset of dysthymia was set as three years of age after consulting the experts. Also, it was consistent with the available data. Excess-mortality was set to 0 as there is no epidemiological evidence to suggest that dysthymia is associated with a statistically significant risk of mortality.

Study-level covariates were used to accommodate between study variability in the raw prevalence data. A lay interviewer covariate created a crosswalk between prevalence derived from clinically trained interviewers (desirable) and prevalence derived from lay-interviewers. As the effect of this covariate was not statistically

significant, it was excluded from the final model. Given that dysthymia is being modelled as a chronic disorder with a long duration of between six and 10 years, significant variation between point and past year prevalence was not detected.

Betas and exponentiated values (which can be interpreted as an odds ratio) for each study level covariate are shown in the table below:

Study covariate	Parameter	Beta	Exponentiated beta
Lay interviewer	Prevalence	-0.37 (-0.51 to -0.24)	0.69 (0.60 0.78)

As there is lack of data available for dysthymia, and the available data were very heterogeneous given differences in the data collection methodology used between studies, a restriction on location was applied with random-effects of -0.3 to 0.3 to further guide the estimation of prevalence.

D.3. Anxiety Disorders

The steps in the estimation of non-fatal anxiety disorders burden or morbidity are shown in the following flowchart:

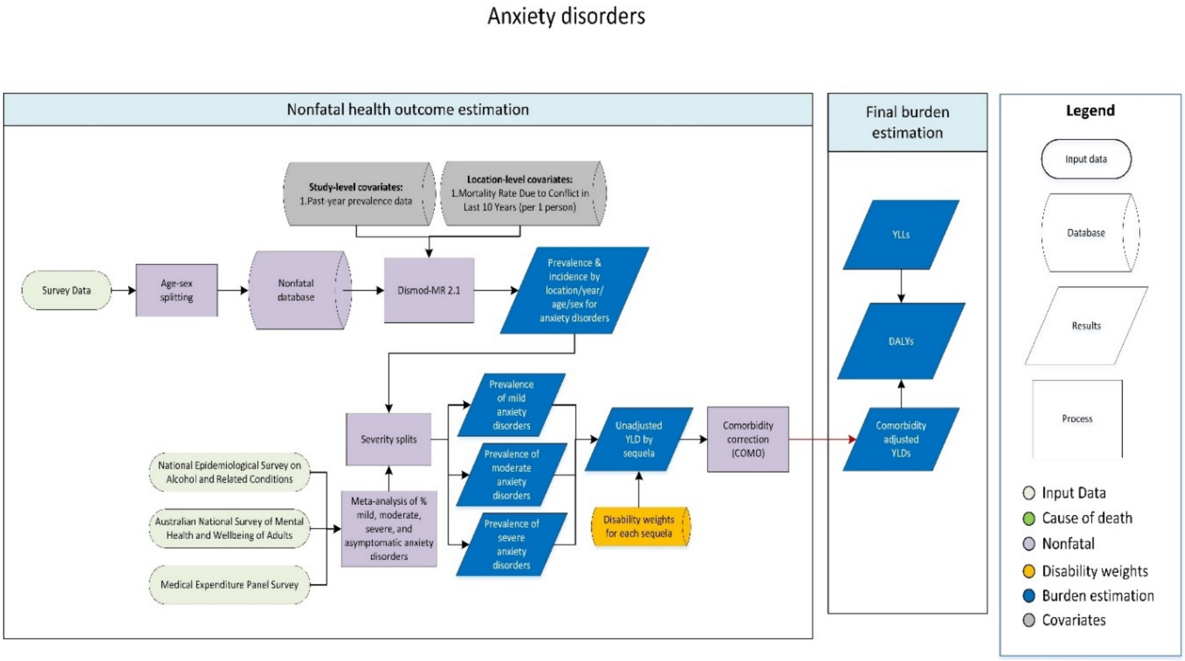

Data

Reported estimates of prevalence of anxiety disorder was split by age and sex where possible. First, if studies reported prevalence for broad age groups by sex (e.g. prevalence in 15 to 65 year old males and females separately), and also by specific age groups but for both sexes combined (e.g. prevalence in 15 to 30 year old, then in 31 to 65 year old, for males and females combined), age-specific estimates were split by sex using the reported sex ratio and bounds of uncertainty. Second, where studies reported estimates across age groups spanning 20 years or more, these were split into five year age groups using the prevalence age pattern estimated by DisMod-MR 2.1.

Severity splits

The basis of the GBD disability weight survey assessments are lay descriptions of sequelae highlighting major functional consequences and symptoms. The lay descriptions and disability weights for anxiety disorder severity levels are shown below.

Severity level	Lay description	Disability weight (95% CI)
Mild	Feels mildly anxious and worried, which makes it slightly difficult to concentrate, remember things, and sleep. The person tires easily but is able to perform daily activities.	0.03 (0.018-0.046)
Moderate	Feels anxious and worried, which makes it difficult to concentrate, remember things, and sleep. The person tires easily and finds it difficult to perform daily activities.	0.133 (0.091-0.186)
Severe	Constantly feels very anxious and worried, which makes it difficult to concentrate, remember things, and sleep. The person has lost pleasure in life and thinks about suicide.	0.523 (0.362-0.677)

Modelling strategy

For anxiety disorders DisMod-MR 2.1 was used for the morbidity estimation. Data across all epidemiological parameters were initially included in the modelling process. The incidence studies reported estimates which were very low relative to the prevalence data. As prevalence studies contributed much greater data coverage than incidence studies, the incidence data were excluded. No incidence and prevalence was assumed to occur before age 2 and after age 95. This minimum age of onset was corroborated with expert feedback and existing literature on anxiety disorders. Remission was set to a maximum of 0.2, consistent with the data points available. Study level covariates were used to accommodate for between study variability in the raw prevalence data. A past year recall covariate adjusted all data points derived from past year prevalence toward the level they would have been if the study had captured point/past-month prevalence. The latter prevalence period is less affected by recall bias. A school survey covariate adjusted estimates derived from school surveys downward to the level they would have been had the study conducted a fully representative population survey. A country level covariate identifying for each GBD location the mean mortality rate in the previous ten years due to war and terrorism was also included in the anxiety disorders model. This informed the estimation of prevalence given existing evidence to show a positive association between conflict status and the prevalence for anxiety disorders. Betas and exponentiated values (which can be interpreted as an odds ratio) for each study-level covariate are shown in the table below:

Study covariate	Parameter	Beta	Exponentiated beta
Past year recall	Prevalence	0.39 (0.34 0.45)	1.48 (1.41 1.56)
School survey	Prevalence	0.43 (0.31 0.56)	1.54 (1.36 1.75)
Mean war mortality rate in the previous 10 years	Prevalence	0.50 (0.027 0.97)	1.65 (1.03 2.65)

D.4. Idiopathic developmental intellectual disability

The steps in the estimation of non-fatal idiopathic developmental intellectual disability burden or morbidity are shown in the following flowchart:

Intellectual Disability

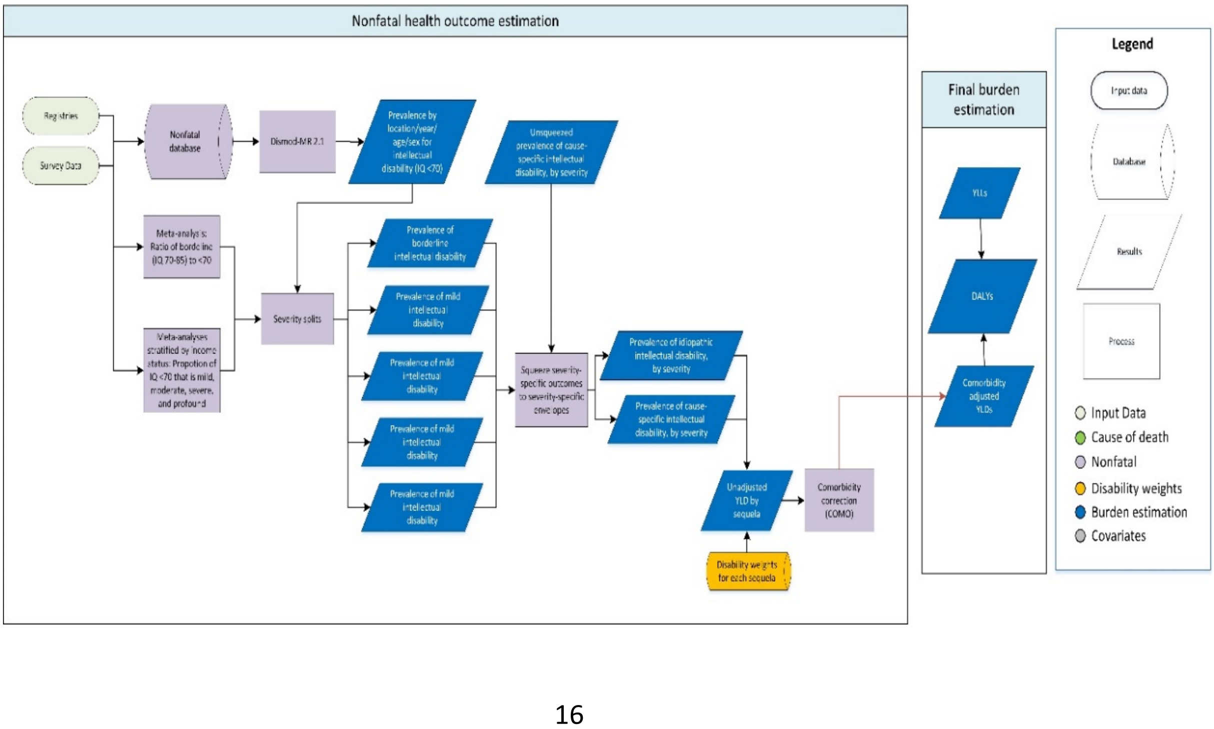

Data

The prevalence of idiopathic developmental intellectual disability was calculated by subtracting all severity and aetiology-specific intellectual disability from the severity-specific envelope assuming the residuals to represent idiopathic disability. If the residual was less than 5% of the severity-specific envelope, the prevalence of all aetiology-specific intellectual disability was proportionally squeezed to fit within 95% of the envelope, leaving 5% for idiopathic intellectual disability.

Severity splits-disability weights

Intellectual disability severity disability weights

Health state	Description	Disability weight (95% CI)
Borderline intellectual functioning	This person is slow in learning at school. As an adult, the person has some difficulty doing complex or unfamiliar tasks but otherwise functions independently.	0.011 (0.005 0.02)
Intellectual disability/mental retardation, mild	This person has low intelligence and is slow in learning at school. As an adult, the person can live independently, but often needs help to raise children and can only work at simple supervised jobs.	0.043 (0.026 0.064)
Intellectual disability/mental retardation, moderate	This person has low intelligence, and is slow in learning to speak and to do even simple tasks. As an adult, the person requires a lot of support to live independently and raise children. The person can only work at the simplest supervised jobs.	0.1 (0.066 0.142)
Intellectual disability/mental retardation, severe	This person has very low intelligence and cannot speak more than a few words, needs constant supervision and help with most daily activities, and can do only the simplest tasks.	0.16 (0.107 0.226)
Intellectual disability/mental retardation, profound	This person has very low intelligence, has almost no language, and does not understand even the most basic requests or instructions. The person requires constant supervision and help for all activities.	0.2 (0.133 0.283)

Modelling strategy

GBD modelled the prevalence of intellectual disability, both aetiology-specific intellectual disabilities and idiopathic intellectual disability over multiple steps.

First, a DisMod-MR 2.1 model was run to estimate the total prevalence of intellectual disability of level IQ <70. Lag-distributed income and education included in the model as predictive covariates. Table below shows raw and exponentiated model coefficients for the covariates used in the estimation process for the DisMod model. Exponentiated coefficients can be interpreted as odds ratios.

Covariate	Parameter	Coefficient (95% CI)	Exponentiated coefficient (95% CI)
Lagged distributed income (LDI) per capita	Prevalence	-0.25 (-0.44-0.068)	0.78 (0.65-0.93)
Underweight (proportion less than 2 SD below the mean weight for age in children under 5)	Prevalence	1.27 (0.09-2.85)	3.57 (1.09-17.34)
Sex	Prevalence	0.28 (0.12-0.44)	1.32 (1.13-1.55)

Second, the total prevalence of idiopathic intellectual disability split into four severity levels: mild (IQ 50-69), moderate (IQ 35-49), severe (IQ 20-34), and profound (IQ below 20). A subset of studies was pooled that distinguished intellectual disability by these severity levels. A cumulative severity levels was used via random effects meta-analyses stratified by two levels of income status (high-income versus low- and middle-income). These proportions were used to estimate discrete severities from the overall intellectual disability (IQ <70) prevalence. Borderline disability (IQ 70-84) was estimated via another random-effects meta-analysis of the ratio of IQ 70-84 to IQ <70. The uncertainty of the pooled fractions and ratios were propagated throughout our calculations using 1,000 draws from a normal distribution with mean and standard error estimated by the meta-analysis. The results of the meta analysis are shown in the table below.

Proportion of intellectual disability cases

Severity	Mean	Standard error
None	0.161	0.034
Borderline	0.161	0.034
Mild	0.375	0.037
Moderate	0.190	0.031

Third, prevalence of each aetiology specific intellectual disability was estimated using models of the following parent causes. Since GBD is modelling only developmental intellectual disability, causes such as stroke and Alzheimer's disease are not included in the causal attribution process.

Parent causes included in causal attribution are:

- Neonatal preterm birth complications (<28w, 28-32w, 32-36w)
- Neonatal encephalopathy due to birth asphyxia and trauma
- Congenital birth defects (diaphragmatic hernia, cardiovascular anomalies)
- Haemolytic disease and other neonatal jaundice
- Meningitis (pneumococcal, H influenzae type B, meningococcal, other bacterial)
- Encephalitis
- Malaria
- Neonatal tetanus
- Neonatal sepsis and other neonatal infections
- Iodine deficiency
- African trypanosomiasis
- Down syndrome
- Klinefelter syndrome
- Chromosomal abnormalities (unbalanced rearrangements, Down syndrome, Edwards syndrome, Patau syndrome, other chromosomal abnormalities)
- Neural tube defects (e.g. spina bifida, encephalocele)
- Hypertensive disorders of pregnancy (eclampsia, preeclampsia)
- Autism spectrum disorders
- Fetal alcohol syndrome

For ASD, six studies were identified reporting severity of ID. A meta-analysis was conducted to produce a severity distribution which then applied to the prevalence of autism to produce severity-specific intellectual disability due to autism.

The prevalence of individual aetiology-specific ID was estimated by models from the respective parent causes, the squeezing may result in a distorted balance of prevalence estimates within their parent causes. With the aim to maintain consistencies of prevalence within each of the parent causes, the difference between the original and the squeezed prevalence estimates were added to the motor impairment sequela if the squeezed sequela represented motor and cognitive impairment. For autism, the fraction of cases was obtained which resulted in ID from literature (0.29; 95% CI 0.27-0.30) and applied this fraction to the subtraction and squeezing processes. All ID cases due to iodine deficiency (cretinism) were assumed to result in either severe or profound disability, and Klinefelter syndrome cases that result in ID will have either borderline or mild severity.

D.5. Schizophrenia

The steps in the estimation of non-fatal schizophrenia burden or morbidity are shown in the following flowchart:

Schizophrenia

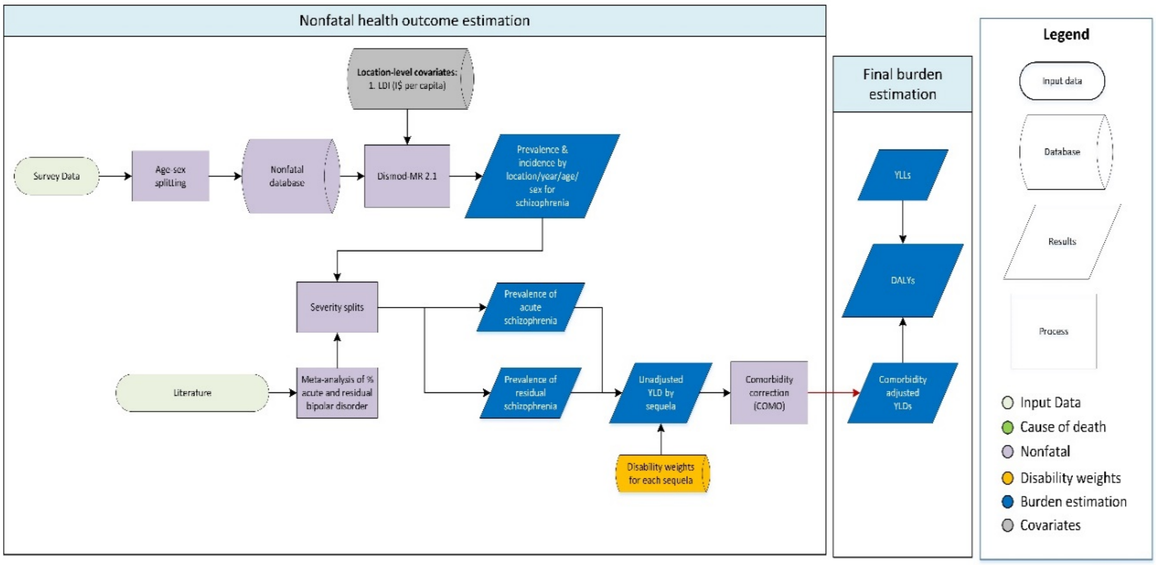

Data

The estimates of prevalence of schizophrenia was split by age and sex where possible. If studies reported prevalence for broad age groups by sex (e.g. prevalence in 15 to 65 year old males and females separately), and also by specific age groups but for both sexes combined (e.g. prevalence in 15 to 30 year old, then in 31 to 65 year old, for males and females combined); age specific estimates were split by sex using the reported sex ratio and bounds of uncertainty. Studies which reported estimates across age groups spanning 20 years or more were split into five year age groups using the prevalence age pattern estimated by DisMod-MR.

Modelling strategy

DisMod-MR 2.1 model was used to estimate prevalence of schizophrenia by age, sex, year, state, and country. Data across all epidemiological parameters were included in the modelling process. It was assumed that no incidence of schizophrenia before the age of 10 and after 80 years of age. This minimum age of onset was verified with expert feedback and existing literature on schizophrenia. Remission was also restricted to a maximum of 0.04 as guided by data available in the dataset.

Study-level covariates were not included for the morbidity estimation of schizophrenia as the tested covariates failed to demonstrate any significance. But a location-level covariate, lagged distributed income (LDI), was included. LDI represents a moving average of gross domestic product (GDP) over time. It was also applied to excess mortality data with a negative relationship assumed. The table below illustrates the covariate, parameter, beta and exponentiated beta values for the model.

Location-level covariate	Parameter	Beta	Exponentiated beta
LDI	Excess mortality rate	-0.55 (-1 to -0.1)	0.58 (0.37-0.90)

D.6. Bipolar disorder

The steps in the estimation of non-fatal bipolar disorder burden or morbidity are shown in the following flowchart:

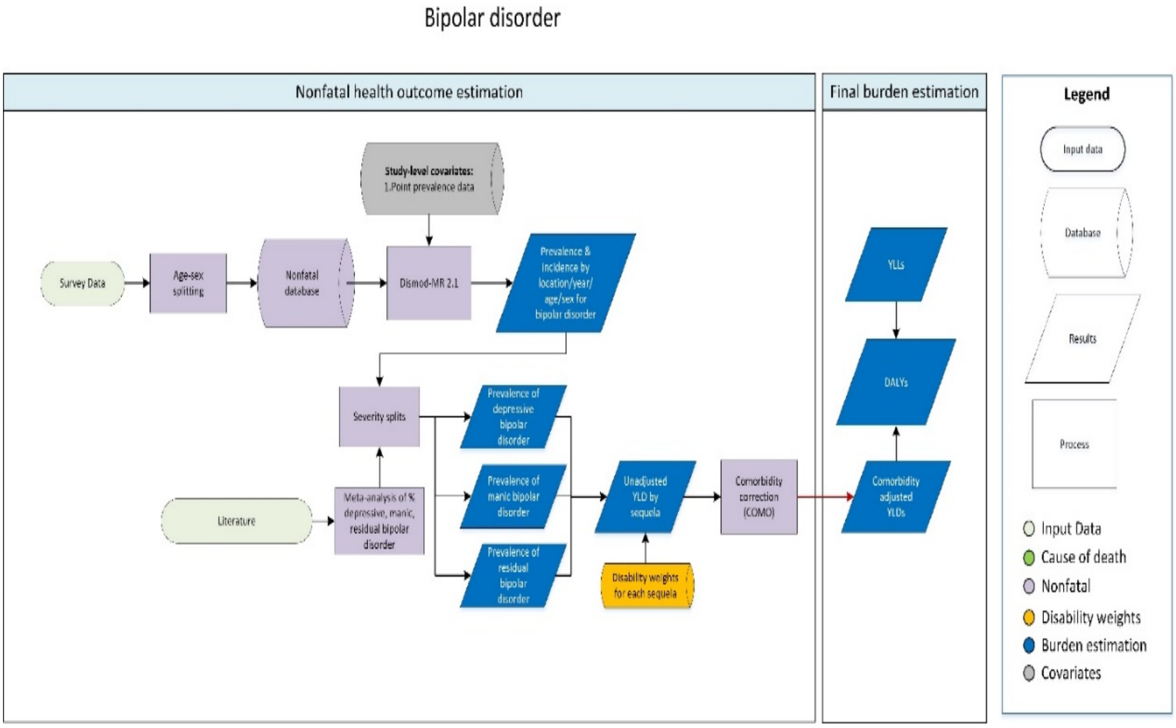

Data

Reported estimates of prevalence of bipolar disorders was split by age and sex where possible. First, if studies reported prevalence for broad age groups by sex (e.g. prevalence in 15 to 65 year old males and females separately) and by specific age groups but for both sexes combined (e.g. prevalence in 15 to 30 year old, then in 31 to 65 year old, for males and females combined), age-specific estimates were split by sex using the reported sex ratio and bounds of uncertainty. Second, where studies reported estimates across age groups spanning 20 years or more, these were split into five-year age groups using the prevalence age pattern estimated by DisMod-MR 2.1.

Severity splits inputs

The basis of the GBD disability weight survey assessments are lay descriptions of sequelae highlighting major functional consequences and symptoms. The lay descriptions and disability weights for bipolar disorder severity levels are shown below.

Severity level	Lay description	Disability weight (95% CI)
Manic	Is hyperactive, hears and believes things that are not real, and engages in impulsive and aggressive behaviour that endanger the person and others.	0.492 (0.341-0.646)
Depressive*	Has constant sadness and has lost interest in usual activities. The person has some difficulty in daily life, sleeps badly, has trouble concentrating, and sometimes thinks about harming himself (or herself).	0.396 (0.267-0.531)
Residual	Has mild mood swings, irritability, and some difficulty with daily activities.	0.032 (0.018-0.051)

Note: *Equivalent to the disability weight estimated for moderate major depressive disorder

Information on the distribution of manic, depressive, and residual states of bipolar disorder was obtained from a separate systematic review of the literature. Meta-XL (a Microsoft Excel add-in for meta-analysis) was used to pool estimates across all studies to calculate the overall proportion of bipolar cases in each health state. Six studies provided information on the proportion of bipolar disorder cases in a manic (21%, 12%-33%), depressive (23%, 10%-39%), or residual state (52%, 28%-77%).

Modelling strategy

The DisMod-MR 2.1 platform was used for the modelling. Data across all epidemiological parameters were initially included in the modelling process. The two studies on incidence reported 0% and 0.1% incidence of bipolar disorder and were low relative to the prevalence data. They were excluded from the final model where incidence was estimated using data from other parameters. No incidence and prevalence was assumed to occur before age 10. Remission was set to a maximum of 0.05 in agreement with literature and expert advice suggesting no or very little complete remission from bipolar disorder.

Study-level covariates were used to accommodate for between study variability in the raw prevalence data. A point recall covariate adjusted all data points derived from point/past-month prevalence toward the level they would have been if the study had captured 12-month prevalence. 12-month prevalence was set as the desirable level due to the episodic nature of bipolar disorder. Estimates of point prevalence surveying symptoms experienced in the past 30 days or less may fail to diagnose cases of bipolar disorder in a residual state, thereby underestimating prevalence.

The corresponding beta and exponentiated value (which can be interpreted as an odds ratio) is shown in the table below:

Study covariate	Parameter	Beta	Exponentiated beta
Point recall	Prevalence	-0.86 (-1.17 to -0.61)	0.42 (0.31-0.54)

Given that there was an overall paucity in epidemiological data available for bipolar disorder, and the data available were very heterogeneous given differences in the data-collection methodology used between studies, a restriction on location random-effects of -0.3 to 0.3 was applied to further guide the estimation of prevalence.

D.7. Conduct disorder

The steps in the estimation of non-fatal conduct disorder (CD) burden or morbidity are shown in the following flowchart:

Conduct disorder

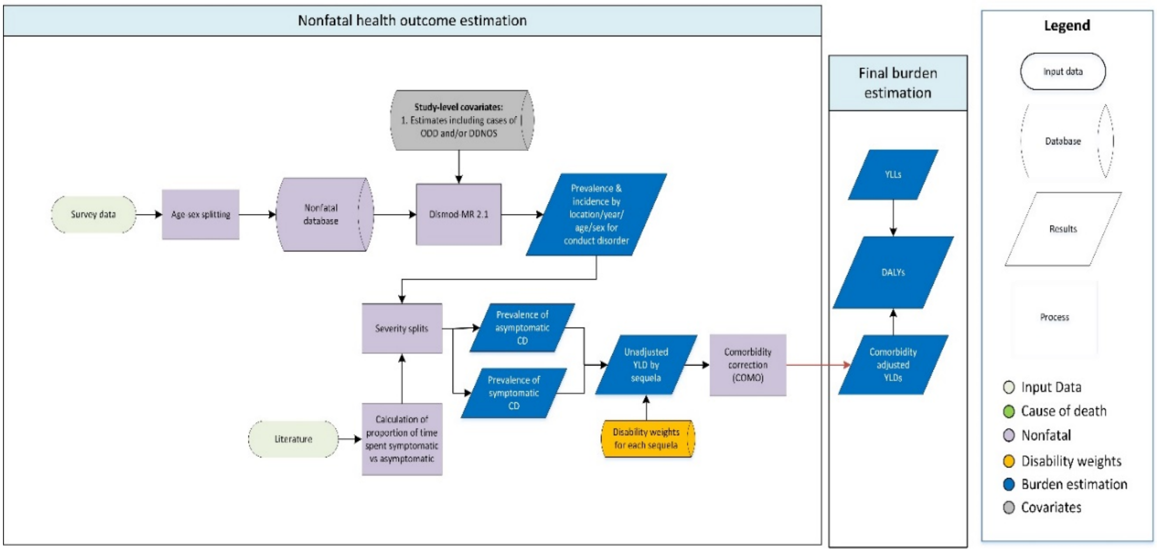

Data

The prevalence of conduct disorders was split by age and sex where possible. If studies reported prevalence for broad age groups by sex (e.g. prevalence in 5-18year old males and females separately) and by specific age groups but for both sexes combined (e.g. prevalence for 5-12 year old and 13-18 year old, for males and females combined), age-specific estimates were split by sex using the reported sex ratio and bounds of uncertainty.

Severity splits and disability weights

A severity split for the proportion of time spent symptomatic versus asymptomatic was based on data from the Great Smoky Mountains Study which assessed the levels of disability found in children and adolescents with mental disorders. Of those with CD, 72% reported disability while 20% of individuals with no diagnosis reported disability at the time of survey. Using these as estimates of the proportion of time with disability in the average case, the proportion of disability in children without a diagnosis was subtracted from the proportion with disability for CD, giving an adjusted proportion of 52%. Detailed descriptions of this methodology have been published elsewhere. The lay description and disability weight for CD is shown in the table below.

Lay description	Disability weight (95% CI)
Has frequent behaviour problems, which are sometimes violent. The person often has difficulty interacting with other people and feels irritable.	0.241 (0.159-0.341)

Modelling strategy

No incidence or prevalence was assumed to occur prior to 5 years of age or after 18 years of age. The minimum age of onset was set in consultation with experts while the upper age limit was set in line with DSM criteria. Excess mortality was set to zero given the absence of data demonstrating an association between CD and an increased risk of death. Remission and incidence were capped between ages 4 and 17 years in order to gain more plausible output. A covariate was used to adjust any prevalence estimates which also included cases of oppositional defiant disorder and/or disruptive behaviour disorder not otherwise specified toward those including CD only.

Covariate	Parameter	Beta	Exponentiated beta
Identifies estimates also containing ODD &/or DDNOS cases	Prevalence	0.63 (0.39 - 0.84)	1.88 (1.48-2.32)

D.8. Anorexia nervosa

The steps in the estimation of non-fatal anorexia nervosa burden or morbidity are shown in the following flowchart:

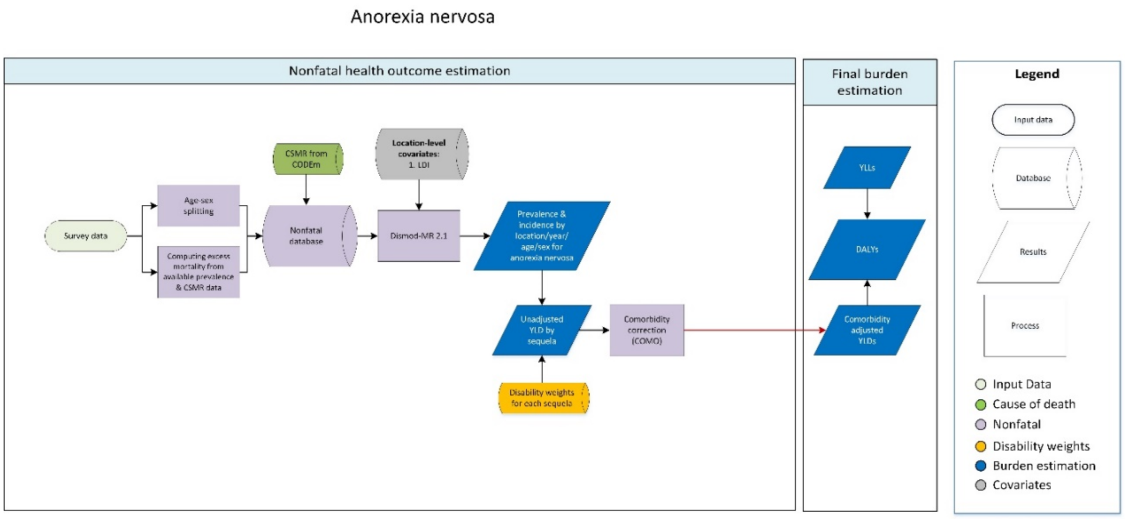

Data

No severity splits were applied to anorexia nervosa after the estimation of prevalence. The lay description and disability weight for anorexia nervosa are shown in the table below.

Lay description	Disability weight (95% CI)
Feels an overwhelming need to starve and exercises excessively to lose weight. The person is very thin, weak, and anxious.	0.224 (0.150-0.312)

Modelling strategy

No incidence was assumed to occur prior to age 5 or from 50 years onward. These settings are in line with those placed on the corresponding cause of death model for anorexia nervosa. A cap of 0.6 was placed on remission in order to obtain a more plausible fit of the model. The function in DisMod-MR 2.1 was used to pull in cause-specific mortality rate (CSMR) data from our CODEm and CoDcorrect analyses. As such, other mortality data (standardised mortality ratios and relative risks) were excluded. CSMR data also used to estimate priors on excess mortality rates (EMR) by matching them with prevalence data points for the same geography and study year and dividing CSMR by prevalence. A country-level covariate LDI, was included. The limits placed on this covariate meant that prevalence was assumed to increase with rising GDP. LDI was also applied to excess mortality data in order to better inform regional distribution. The table below illustrates the covariates, parameters, beta and exponentiated beta values for anorexia nervosa.

Covariate	Parameter	Beta	Exponentiated beta
LDI ($ per capita)	Prevalence	0.42 (0.23-0.50)	1.52 (1.26-1.64)
LDI ($ per capita)	Excess mortality	-0.26 (-0.48 -0.11)	0.77 (0.62-0.90)

D.9. Bulimia nervosa

The steps in the estimation of non-fatal bulimia nervosa disease burden or morbidity are shown in the following flowchart:

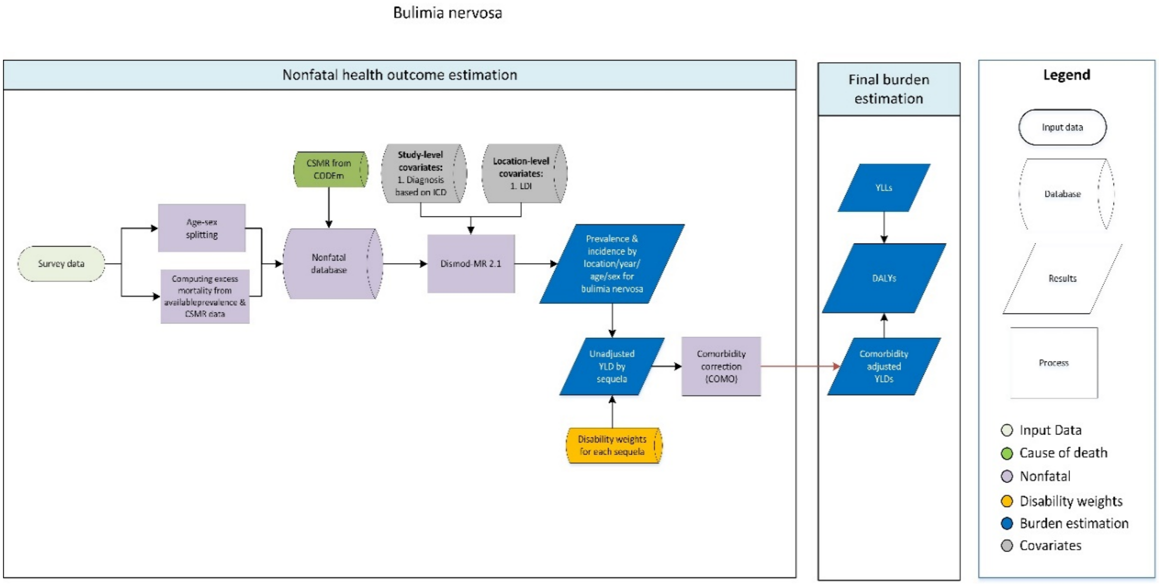

Data

No severity splits were applied to bulimia nervosa as well. The lay description and disability weight for bulimia nervosa is shown in the table below.

Lay description	Disability weight (95% CI)
Has uncontrolled overeating followed by guilt, starving, and vomiting to lose weight.	0.223 (0.149-0.311)

Modelling strategy

No incidence was assumed to occur prior to 10 years of age or onward from 40 years of age. The function in DisMod-MR 2.1 was used to pull in cause-specific mortality rate (CSMR) data from our CODEm and

COD correct analyses. As such, other mortality data (standardised mortality ratios and relative risks) were excluded. CSMR data was also used to estimate priors on excess mortality rates (EMR) by matching them with prevalence data points for the same geography and study year and dividing CSMR by prevalence. A study-level covariate was applied which adjusted estimates based on ICD criteria toward those based on DSM criteria. A country-level covariate, lagged distributed income (LDI), was also included. The limits placed on this covariate meant that prevalence was assumed to increase with rising GDP. LDI was also applied to excess mortality data in order to better inform regional distribution. The table below illustrates the covariates, parameters, beta and exponentiated beta values for BN.

Covariate	Parameter	Beta	Exponentiated beta
ICD classification	Prevalence	-0.17 (-0.67-0.39)	0.84 (0.51-1.48)
LDI	Prevalence	0.39 (0.15-0.50)	1.48 (1.16-1.64)
LDI	Excess mortality	-0.3 (-0.49-0.11)	0.74 (0.61-0.90)

D.10. Autism spectrum disorders

The steps in the estimation of non-fatal autism spectrum disorder burden or morbidity are shown in the following flowchart:

Autism

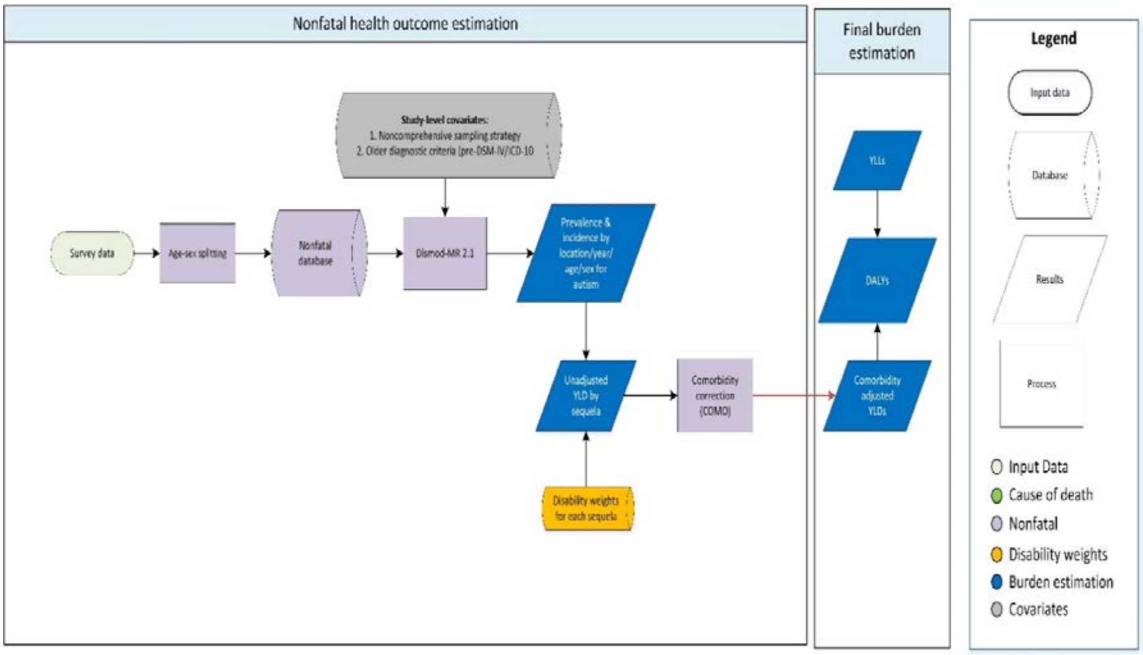

Data

Prevalence estimates of autism spectrum disorders (ASD) was split by age and sex where possible outside of DisMod-MR 2.1. First, if studies reported prevalence for broad age groups by sex (e.g. prevalence in 15 to 65 year-old males and females separately), and also by specific age groups but for both sexes combined (e.g. prevalence in 15 to 30 year-old, then in 31 to 65 year old, for males and females combined); age-specific estimates were split by sex using the reported sex ratio and bounds of uncertainty. Studies that only reported the prevalence of autism rather than ASD were included but adjusted up by a factor of 2.31 (se = 0.20) based on 18 studies that used gold-standard sampling methodology and reported prevalence for both ASD and autism.

Severity split inputs

ASD is one of the causes that contribute to the intellectual disability (ID) envelope. As such, a gradation of ASD by level of severity was needed.

Meta-analyses were conducted using data from 19 studies that used gold-standard sampling methodology and reported information on the IQ level in those with ASD in order to calculate the severity splits by six sequelae: ASD with no ID, borderline ID, mild ID, moderate ID, severe ID, and profound ID.

The disability weights for each sequela of ASD were calculated using the disability weights for the health states Autism, Asperger's syndrome and other autism spectrum disorders, borderline ID, mild ID, moderate ID, severe ID and profound ID. These disability weights and their lay descriptions are presented in the table below.

Health state	Lay description	Disability weight (95% CI)
Autism	Has severe problems interacting with others and difficulty understanding simple questions or directions. The person has great difficulty with basic daily activities and becomes distressed by any change in routine.	0.262 (0.176-0.365)
Asperger's syndrome and other autism spectrum disorders	Has difficulty interacting with other people and is slow to understand or respond to questions. The person is often preoccupied with one thing and has some difficulty with basic daily activities.	0.104 (0.071-0.147)
ID, borderline	Is slow in learning at school. As an adult, the person has some difficulty doing complex or unfamiliar tasks but otherwise functions independently.	0.011 (0.005-0.020)
ID, mild	Has low intelligence and is slow in learning at school. As an adult, the person can live independently, but often needs help to raise children and can only work at simple supervised jobs.	0.043 (0.026-0.064)
ID, moderate	Has low intelligence, and is slow in learning to speak and to do even simple tasks. As an adult, the person requires a lot of support to live independently and raise children. The person can only work at the simplest supervised jobs.	0.100 (0.066-0-142)
ID, severe	Has very low intelligence and cannot speak more than a few words, needs constant supervision and help with most daily activities, and can do only the simplest tasks.	0.160 (0.107-0.226)
ID, profound	Has very low intelligence, has almost no language, and does not understand even the most basic requests or instructions. The person requires constant supervision and help for all activities.	0.200 (0.133-0.283)

To estimate the disability weights for each sequela of ASD, the following steps were conducted, each step pulling 1,000 draws of each input:

1. A pooled disability weight for ASD was estimated:

$$DW_{ASD} = DW_{Autis} \times A + DW_{Asperger} \times (1 - A)$$

Where DW is disability weight and   is the inverse of the autism-to-ASD adjustment described earlier (= 0.43, se = 0.04).

2. The disability weight for ASD without ID was estimated:

$$DW_{ASD\ no\ ID} = \frac{DW_{ASD} - \sum_{k=Bord.ID}^{Prof.ID}(P_k \times DW_k)}{P_{ASD\ no\ ID} + \sum_{k=Bord.ID}^{Prof.ID}(P_k \times (1 - DW_k))}$$

Where DW is disability weight and P is the severity proportion estimated from the meta-analysis.

3. The disability weight for ASD and each remaining level of ID was estimated:

$$DW_{ASD+ID} = 1 - (1 - DW_{ASD\ no\ ID}) \times (1 - DW_{ID})$$

The severity proportions from the meta-analysis used in the above process and the resulting disability weights for each sequela are presented in the table below.

Sequela	Severity proportion (95% UI)	Disability weight (95% UI)
ASD without ID	0.428 (0.369-0.491)	0.143 (0.094-0.202)
ASD with borderline ID	0.187 (0.144-0.236)	0.152 (0.103-0.212)
ASD with mild ID	0.180 (0.134-0.231)	0.179 (0.125-0.245)
ASD with moderate ID	0.133 (0.094-0.177)	0.228 (0.160-0.310)
ASD with severe ID	0.057 (0.034-0.091)	0.279 (0.195-0.378)
ASD with profound ID	0.014 (0.006-0.025)	0.313 (0.215-0.422)

Modelling strategy

All incidence of ASD was assumed to occur at birth. A small setting was placed on excess mortality to help DisMod follow the mortality estimates. Remission was set to 0 after expert consultation which revealed that remission for ASD was not expected. Three study-level covariates were applied to adjust estimates with suboptimal sampling methodologies:

- Survey: Studies that conduct household or school surveys but do not conduct additional active case-finding (such as reviewing special education records) to find cases likely to be missed by survey methodology.
- Registry data: Studies where prevalence of ASD is estimated from diagnoses within a clinical or educational registry where no population screening procedure is in place.
- Surveillance/notification data: Studies where researchers review notes of high-risk populations from one or more data sources records (e.g. clinical/education records) and determine prevalence based on notes without confirming the diagnosis via clinical evaluation.

Systematic review revealed four studies that used gold-standard sampling methodology to estimate prevalence and also reported the proportion of their cases of ASD that were captured by registries. The pooled proportion was 0.71 (S. E=0.05), and this was set as the prior for the study-level covariate for registry data.

Study covariate	Parameter	Beta	Exponentiated beta
Survey	Prevalence	-0.14 (-0.36-0.11)	0.87 (0.70-1.11)
Registry data	Prevalence	-0.34 (-0.34-0.34)	0.71 (0.71-0.71)
Surveillance / notification data	Prevalence	0.39 (0.21-0.58)	1.48 (1.23-1.78)

D.11. Attention-deficit/hyperactivity disorder

The steps in the estimation of non-fatal attention-deficit/hyperactivity disorder (ADHD) burden or morbidity are shown in the following flowchart:

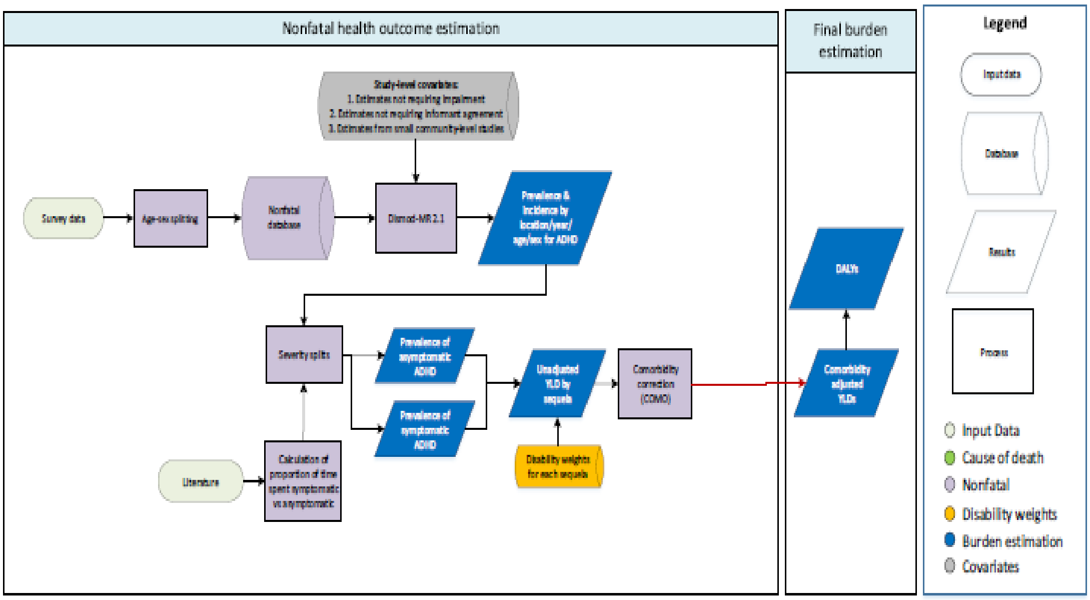

Data

The prevalence of ADHD was split by age and sex where possible. If studies reported prevalence for broad age groups by sex (e.g. prevalence in 5 to 18 year old males and females separately) and by specific age groups but for both sexes combined (e.g. prevalence for 5 to 12 year old and 13 to 18 year old, for males and females combined), age-specific estimates were split by sex using the reported sex ratio and bounds of

uncertainty. Also, where studies reported estimates across age groups spanning 20 years or more, these were split into five-year age groups using the prevalence age pattern estimated by DisMod-MR 2.1.

Severity splits and disability weight

A severity split for the proportion of time spent symptomatic versus asymptomatic was based on data from the Great Smoky Mountains Study which assessed the levels of disability found in children and adolescents with mental disorders. Of those with ADHD, 48% reported disability while 20% of individuals with no diagnosis reported disability at the time of survey. Using these as estimates of the proportion of time with disability in the average case, the proportion of disability in children without a diagnosis was subtracted from the proportion with disability for ADHD, giving an adjusted proportion of 28%. Detailed descriptions of this methodology have been published elsewhere. The lay description and disability weight for ADHD is shown in the table below:

Lay description	Disability weight (95% CI)
Is hyperactive and has difficulty concentrating, remembering things, and completing tasks	0.045 (0.028-0.066)

Modelling strategy

It was assumed that no incidence prior to 3 years of age or onward from 12 years of age occurred. The minimum age of onset was set in consultation with experts and based on current literatures, while the upper age limit on incidence was set in line with the latest DSM-5 criteria. Remission was set to zero prior to 12 years, in line with the restriction on incidence. Excess mortality was set to zero given only three estimates were found for this parameter. Three covariates were included in the model. The first covariate was an informant covariate which adjusted estimates not requiring agreement between informants (e.g. diagnosis made if either a teacher or parent indicates ADHD. The second covariate adjusted estimates not requiring impairment (or those not specifying whether impairment was required) for diagnosis toward those which required impairment. The third covariate adjusted studies using small, community samples toward studies representative of entire regions or countries. Bounds for these covariates were calculated from the epidemiological data and applied in DisMod-MR 2.1.

Study covariate	Parameter	Beta	Exponentiated beta
No informant agreement	Prevalence	0.49 (0.45-0.57)	1.63 (1.57-1.78)
No impairment	Prevalence	0.039 (0.0034-0.13)	1.04 (1.00-1.13)
Small, community-level studies	Prevalence	0.52 (0.33-0.74)	1.68 (1.38-2.09)

D.12. Other mental disorders

The steps in the estimation of non-fatal other mental disorder burden or morbidity are shown in the following flowchart:

Other mental disorders: Personality disorders

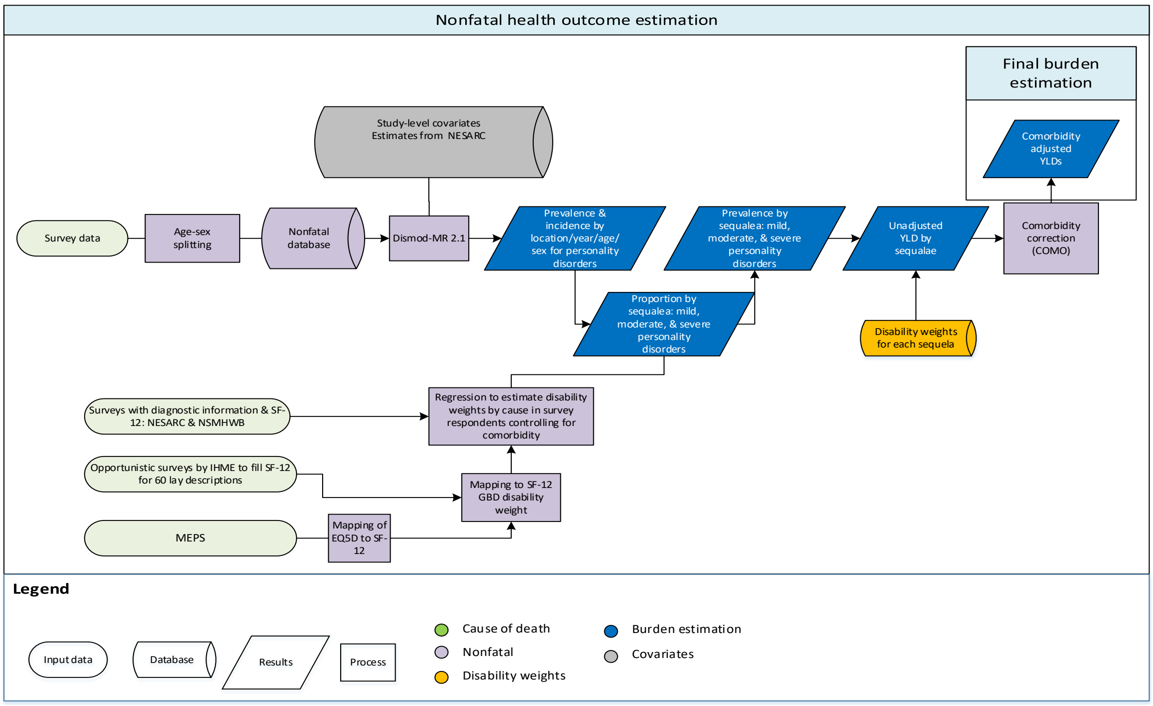

Data

Participants meeting criteria for any type of personality disorders from the main data sources were counted as a prevalent case only if they did not simultaneously meet criteria for another mental and substance use disorder featured in GBD 2017.

Severity splits

The basis of the GBD disability weight survey assessments are lay descriptions of sequelae highlighting major functional consequences and symptoms. The lay descriptions and disability weights applied to the personality disorders within this residual group are shown below and were those estimated for anxiety disorders.

Severity level	Lay description	Disability weight (95% CI)
Mild	Feels mildly anxious and worried, which makes it slightly difficult to concentrate, remember things, and sleep. The person tires easily but is able to perform daily activities.	0.03 (0.018-0.046)
Moderate	Feels anxious and worried, which makes it difficult to concentrate, remember things, and sleep. The person tires easily and finds it difficult to perform daily activities.	0.133 (0.091-0.186)
Severe	Constantly feels very anxious and worried, which makes it difficult to concentrate, remember things, and sleep. The persons has lost pleasure in life and think about suicide.	0.523 (0.362-0.677)

Modelling Strategy

The GBD 2017 epidemiological modelling strategy made use of DisMod-MR 2.1. As only prevalence data was available, a number of expert priors were used in order to run a full-parameter model. It was assumed there was no incidence and prevalence before age 14. This minimum age of onset was verified with expert feedback and DSM criteria highlighting the fact that personality disorders typically become recognisable during adolescence and early adulthood. Remission was set to a maximum of 0.01, given that these are

understood to be chronic disorders with little or no complete remission. Excess mortality was set to 0 in this model, in the absence of mortality data required for DisMod-MR 2.1 modelling purposes.

E. Mental disorders mortality estimation

Eating disorders were the only mental disorder in GBD 2017 to which deaths could be directly attributed. These were estimated from global data, as no direct data from India are available for this. Mortality estimation and modelling methods for the eating disorders are described below.

E.1. Anorexia nervosa

The approach to cause of death estimation of anorexia nervosa are shown in the following flowchart:

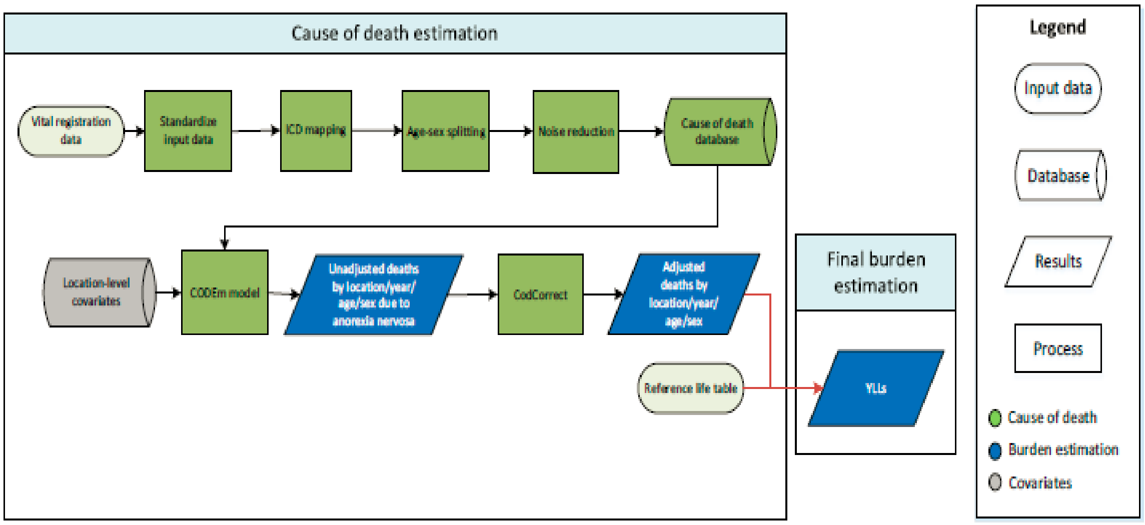

Modelling strategy

Anorexia nervosa was modelled using the standard CODEm approach and came under the eating disorders parent model. CODEm is the framework used to model most cause-specific death rates in the GBD. Further details of CODEm can be found in the appendix to the GBD 2017 cause of death capstone paper (Lancet 2018; 392: 1736–88).

Age was restricted to deaths occurring between 5 and 49 years based on expert advice and patterns of prevalence seen in the non-fatal model. Several covariates were applied to this model and are listed in the table below, along with the direction in which they were applied.

Level	Covariate	Direction
1	Education (years per capita)	+
	Log LDI (I$ per capita)	+
	Underweight (proportion <2SD weight for age, <5 years)	-
	Sanitation (proportion with access)	+
	Maternal education (years per capita)	+
2	Healthcare access and quality index	-
3	Socio-demographic Index	+

E.2. Bulimia nervosa

The approach to cause of death estimation of bulimia nervosa are shown in the following flowchart:

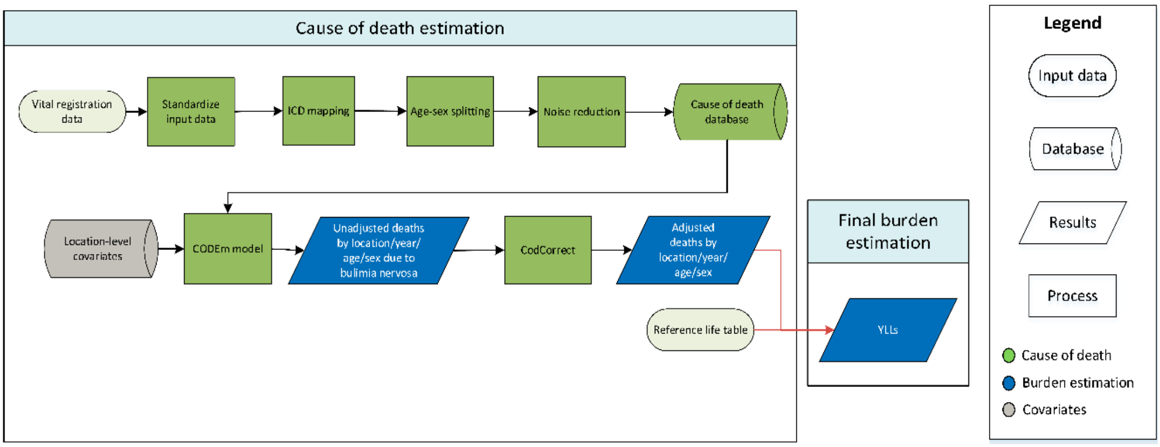

Modelling strategy

Bulimia nervosa was modelled using the standard CODEm approach and comes under the eating disorders parent model. Age was restricted to deaths occurring between 5 and 49 years based on expert advice and patterns of prevalence seen in the non-fatal model. Several covariates were applied to this model and are listed in the table below, along with the direction in which they were applied.

Level	Covariate	Direction
1	Education (years per capita)	+
	Log LDI (I$ per capita)	+
	Underweight (proportion <2SD weight for age, <5 years)	-
	Sanitation (proportion with access)	+
	Maternal education (years per capita)	+
2	Healthcare access and quality index	-
3	Socio-demographic Index	+

F. GBD estimation process of risk factors including mental disorders

The analytical approach used in GBD 2017 for comparative risk assessment to estimate population attributable fractions for risk factors is shown in the following flowchart:

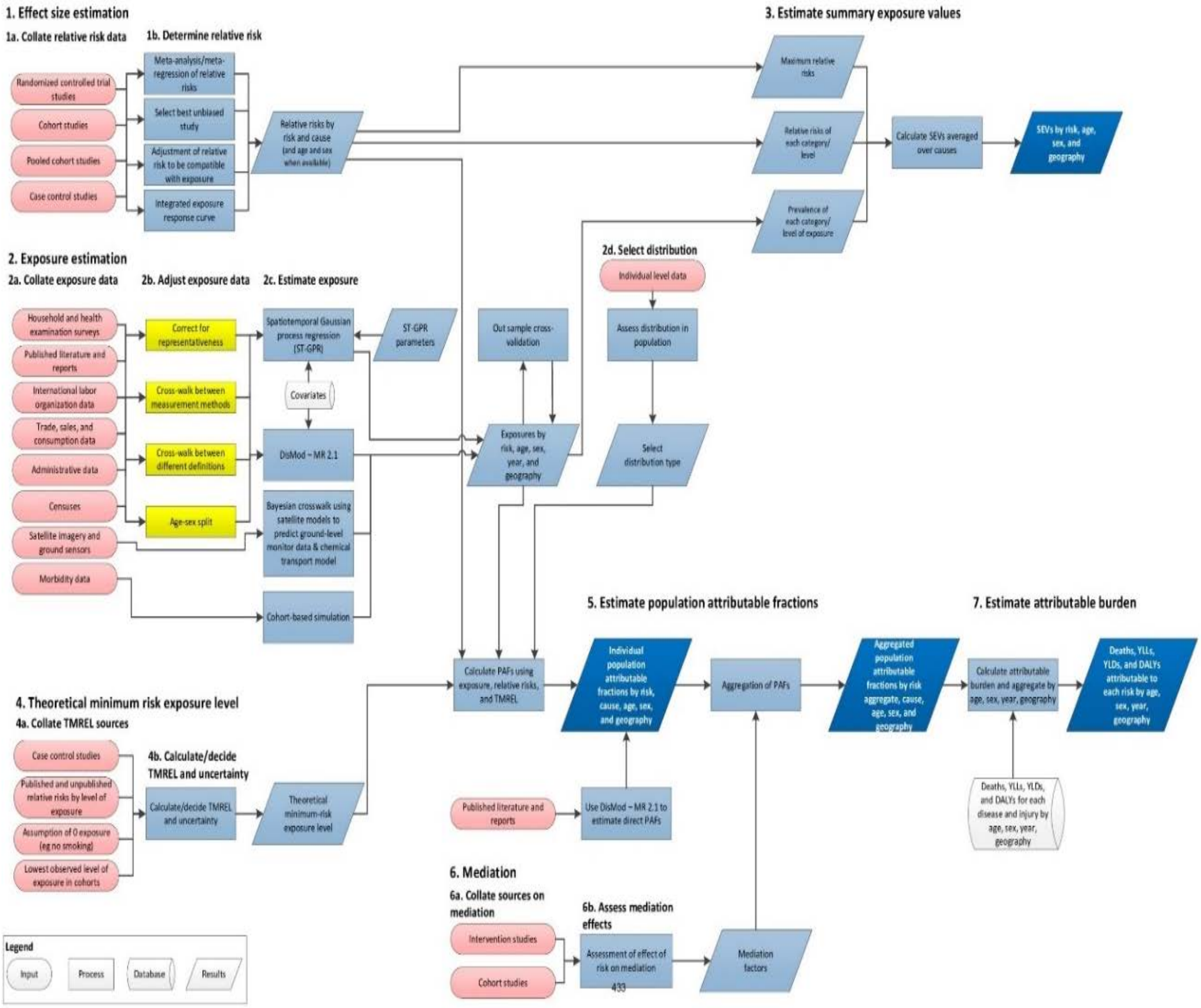

GBD is Global Burden of Disease. SEV is summary exposure value. TMREL is theoretical minimum-risk exposure level. PAF is population attributable fraction. YLL is years of life lost. YLD is years lived with disability. DALY is disability adjusted life-years. Ovals represent data inputs, rectangular boxes represent analytical steps, cylinders represent databases, and parallelograms represent intermediate and final results.

The details of the major risk factors related to mental disorders, i.e. bullying victimisation, childhood sexual abuse, intimate partner violence, and lead exposure are described here. Description of other risk factors can be found in the GBD 2017 risk factor paper (Lancet 2018; 392: 1923–45).

F.1. Bullying victimisation

The steps in the estimation of bullying victimisation are shown in the following flowchart:

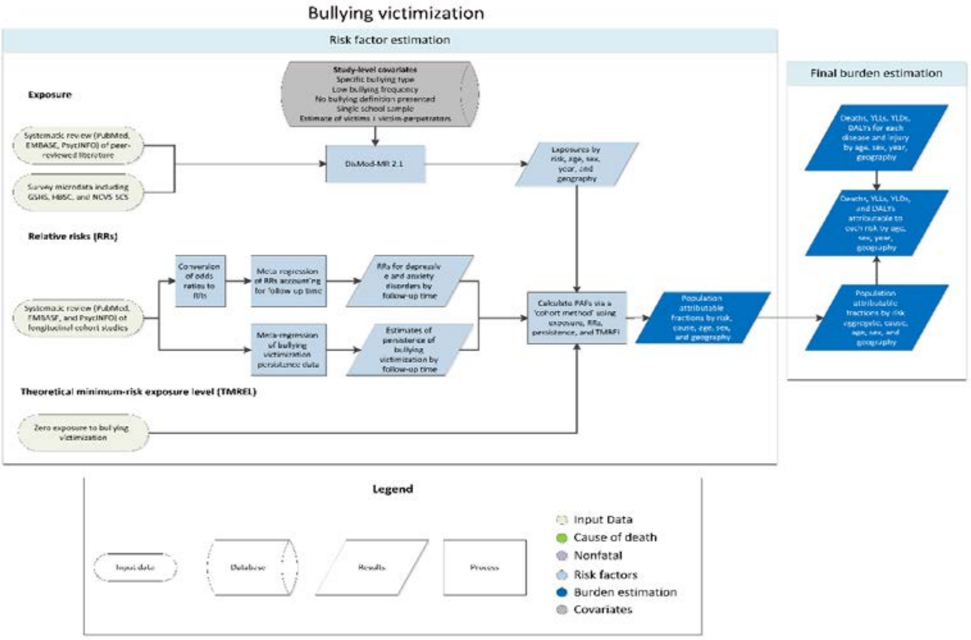

Bullying victimisation is commonly conceptualised as the intentional and repeated harm of a less powerful individual by peers. This differentiates bullying victimisation from disagreements, conflicts, or playful teasing. The case definition of bullying victimisation in the GBD context is 'bullying victimisation of children and adolescents attending school by peers. This definition includes the global concept of bullying victimisation which incorporates combined estimates of subtypes such as physical, verbal, relational, and cyberbullying victimisation. It excludes abuse/harassment by siblings, intimate partners, and adults (e.g. teachers). While bullying can be experienced as either a victim or perpetrator, perpetration (i.e. those who bully others) is not included in this definition although some victims will also be perpetrators.

Data

Population-representative studies globally, including published and unpublished studies, were used for the risk estimation of bullying victimisation.

Modelling strategy

Bullying victimisation prevalence was modelled as a single parameter prevalence model in DisMod-MR 2.1. Prior to 5 years or after 20 years of age no prevalence was assumed. Four study-level covariates were included in the modelling and are shown in the table below, along with their respective levels. Crosswalks for two of the covariates (low bullying frequency and no bullying definition presented) were calculated using the study pairs of reference and non-reference estimates (n = 9 pairs and n = 3 pairs, respectively).

Covariate name	Reference	Non-reference	Exponentiated beta
Low bullying frequency	Optimal frequency threshold used e.g. 'frequently'	Sub-optimal frequency threshold used e.g. 'sometimes + frequently'	3.35 (3.35–3.35) *(n = 9 pairs)*
No bullying definition presented	Definition of bullying victimisation presented to participants	No definition of bullying victimisation presented to participants or not specified	1.12 (1.12–1.12) *(n = 3 pairs)*
Recall 1 year	Asked about bullying victimisation more recently than in the past year	Asked about bullying victimisation in the past year	1.47 (1.30–1.68)
Single school sample	Sample was a household survey or multi-school survey	Sample was from a single school	1.21 (1.01–2.12)

Adjustment for years of schooling

In order to better represent the prevalence of bullying victimisation, prevalence estimates were adjusted for the proportion of children and adolescents attending school by ages 5 to 9, 10 to 14, and 15 to 19 years by sex, location, and year. Data on the proportion of children and adolescents attending school was sourced from the online database (http://data.uis.unesco.org/) published by the United Nations Educational, Scientific, and Culture Organisation (UNESCO). The data covered 18,441 country-years for age groups 6 to 11, 12 to 14, and 15 to 17 years by sex. This data was modelled in the spatio-temporal Gaussian process regression (ST-GPR), with average years of education as a country-level covariate, to predict the proportion of children and adolescents attending school by these age groups. This gave estimates of the proportion of children and adolescents attending school by age, sex, year, and location.

Theoretical minimum-risk exposure level

The theoretical minimum risk exposure level was assumed to be zero exposure to bullying victimisation.

Relative risks

Burden attributable to bullying victimisation for major depressive disorder and anxiety disorders were estimated. Data on the association between bullying victimisation and self-harm was also reviewed but not included due to variation in the definition of 'self-harm' and only one study was looking at suicide.

PAF calculations

For bullying victimisation, the PAF calculations could not be determined by current prevalence and a single value for relative risk (RR). This is due to the waning effect on outcomes over time and because prevalence estimates were from surveys of young people reporting current bullying victimisation rather than estimates of past exposure at the time the outcomes occur (i.e. retrospective estimates).

A cohort method was subsequently developed to address this issue. The following steps are conducted for each point of estimation (i.e. by age, sex, location, and year), hereafter referred to as a 'cohort':

- Pull current and past bullying victimisation prevalence for the cohort from the DisMod-MR 2.1 exposure model.
- Adjust each bullying victimisation prevalence estimate for the proportion of the cohort attending school in that period.
- Divide the cohort into proportions based on time since first exposed to bullying victimisation. This equates to the incidence of bullying victimisation and is estimated using the following formula:

$$I_k = P_k - \sum_{n=0}^{k-1} (I_n \times r_{k-n})$$

where I represent incidence, P represents prevalence, r represents the estimate of persistence, and k represents the time between the incidence estimate and the earliest possible time of exposure in the cohort. I k requires I 0 through to I k-1 to first be calculated and so this process was completed by first estimating I 0, then I 1, and so on until the estimated incidence for the latest possible year of exposure for this cohort were found. The persistence estimate is based on a separate meta-regression of seven studies.

- RRs were mapped to the proportions of the cohort based on the time between the point of estimation and when they were first exposed to bullying victimisation and estimated PAFs via the following formula:

$$PAF = \frac{\sum(p_t \times RR_t) + \sum p_{no\ exposure} - 1}{\sum(p_t \times RR_t) + \sum p_{no\ exposure}}$$

Where t is the time since first exposed to bullying victimisation, p is the proportion of the cohort

first exposed to bullying victimisation at time t or the proportion not exposed to bullying victimisation, and RR is the relative risk for depressive and anxiety disorders given t.

F.2. Childhood sexual abuse

The steps in the estimation of childhood sexual abuse (CSA) are shown in the following flowchart:

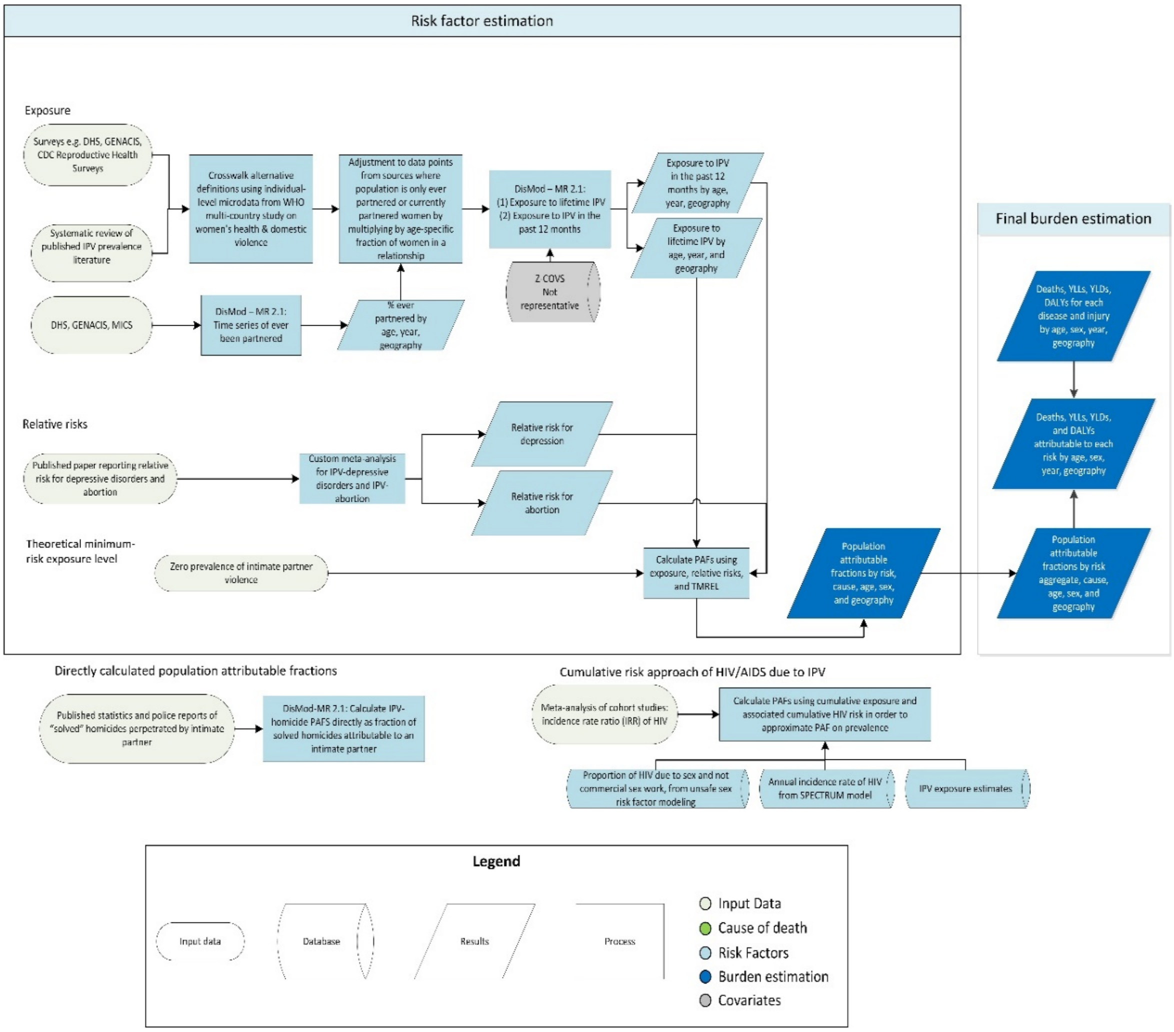

Data

Population-representative studies globally, including published and unpublished studies, were used for the risk estimation of childhood sexual abuse.

Modelling strategy

CSA prevalence was modelled as a single parameter prevalence modelled in DisMod-MR 2.1. CSA exposure is modelled separately for males and females because little correlation was observed between the prevalence of child abuse among females and males, and modelling both sexes together causes unreasonable estimates in countries where data was available for only one sex.

Three study-level covariates were used for alternate definitions of the violence.

- Study asked only about intercourse CSA
- Study asked about contact and non-contact CSA
- Study placed restrictions on the relationship between the perpetrator and the victim (e.g. only asked about CSA committed by a father)

Study-level fixed effects for varying age thresholds across studies were also included.

- Study asked about recall for events before ages above 15 years (versus reference age threshold of 15)
- Study asked about recall for events before ages less than 15 years (versus reference age threshold of 15)

Two study-level covariate fixed effects on variance (z-cov) were also included in both the male and female models, including an indicator that the survey was not nationally representative, as well as whether the survey was administered in schools. These study-level covariates were tested as x-covs first, but coefficients which would indicate systematic bias was not found. Any national-level covariates were not included due to lack of knowledge about a covariate (for which GBD have a time series for all GBD locations) that predicts CSA prevalence.

Theoretical minimum-risk exposure level

The theoretical minimum risk exposure level is zero exposure to contact CSA.

Relative risks

The burden attributable to CSA was estimated for unipolar depressive disorders (major depressive disorder and dysthymia).

F.3. Intimate partner violence

The steps in the estimation of intimate partner violence (IPV) are shown in the following flowchart:

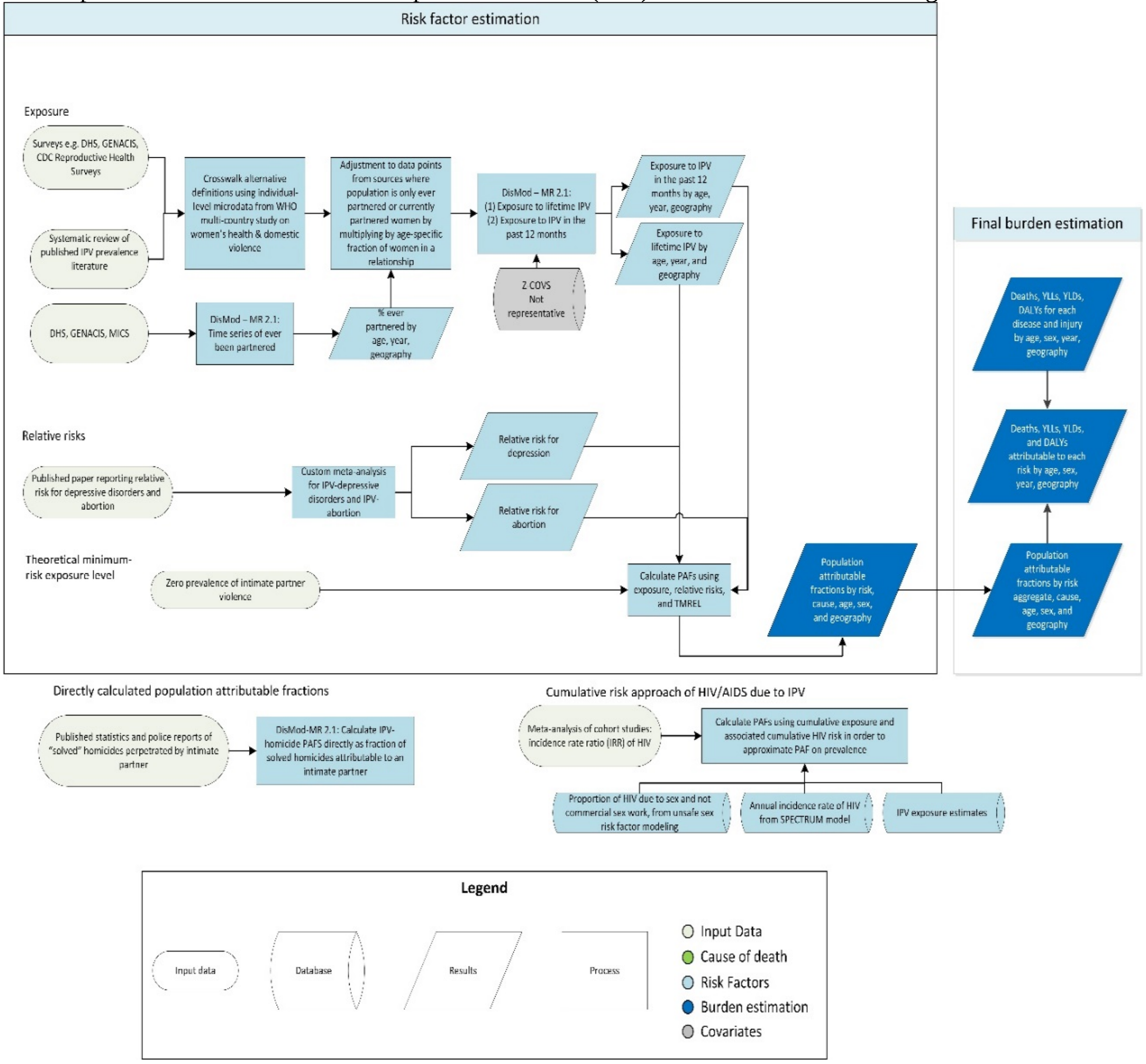

Data

Population-representative studies, including published and unpublished studies, were used for the risk estimation of IPV.

Modelling strategy

Three distinct approaches were used to estimate burden attributable to IPV, including

- the traditional exposure and relative risk (RR) to percent attributable fraction (PAF) method for depression and abortion;
- the direct PAF approach for estimating the proportion of homicides that are perpetrated by an intimate partner; and
- a cumulative risk approach for estimating the burden of HIV/AIDS attributable to IPV.

Estimating attributable burden to IPV for depression, suicide and abortion

Data with variable recall periods (previous 12 months versus lifetime), type of violence (sexual, physical, or both) and severity (severe only versus all levels) were first adjusted. To convert data to the reference definition of ever having experienced any physical or sexual IPV, the data from the WHO Multi-Country Study on Women's Health and Domestic Violence against Women were used to construct crosswalk regressions. The dependent variable in each of these regressions was ever any IPV, while the key independent variable was one of the 11 alternative metrics of IPV that were represented in our dataset:

1) Physical IPV in the past 12 months
2) Sexual IPV in the past 12 months
3) Severe IPV in the past 12 months
4) Severe physical IPV in the past 12 months
5) Severe sexual IPV in the past 12 months
6) Any IPV (physical and/or sexual) in the past 12 months
7) Ever any physical IPV
8) Ever any sexual IPV
9) Ever any severe IPV
10) Ever severe physical IPV
11) Ever severe sexual IPV

For alternate metrics 1-6 a series of age dummies were included:

$$logit(REF_{ait}) = \beta_0 + \beta_1 logit(ALT_{ait}) + \beta_2 I_a + \varepsilon$$

For alternate metrics 7-11, the following regression was run:

$$logit(REF_{it}) = \beta_0 + \beta_1 logit(ALT_{it}) + \varepsilon$$

Where REF is the reference metric of IPV prevalence, ALT is the alternate metric of IPV prevalence, I_a refers to the complete set of age-group indicators, a refers to an age-group, i refers to a country, and t refers to year. Age-group indicators were included in the first six regressions because the prevalence of recent IPV was expected to vary by age. Using the intercepts, coefficients, and variance-covariance matrix from each of these eleven regressions, all of the alternate metrics of IPV prevalence in the dataset were converted to estimates of "ever any IPV." Observations were eliminated based on alternate metrics of IPV which came from studies that also provided estimates of IPV based on the reference definition.

After applying crosswalks to the alternate metrics of IPV in the manner described above, an additional adjustment was made to the subset of data that was based on only ever-partnered, currently partnered women currently married women or ever married women. To adjust these values so that they reflect IPV prevalence in the entire female population, regardless of partnered status, estimates from these studies were multiplied by the age-specific fraction of women who had ever been partnered.

After these pre-DisMod crosswalks and adjustments, a single-parameter prevalence model was run in

DisMod-MR 2.1 with age mesh points at 0, 14, 15, 20, 30, 40, 50, 60, 80 and 100. A study-level fixed effect on integrand variance (z-cov) to indicate whether a study was nationally representative or not was used to account for the heterogeneity introduced by studies that are not generalizable to the entire population. This covariate was first tested as an x-cov and the coefficient indicated no systematic bias.

In addition to the lifetime exposure model, a 12-month exposure model was also run in DisMod-MR 2.1, with data collected and processed analogously. This 12-month exposure model was used for the IPV-abortion PAF calculation to match the exposure definition in the risk evidence.

Direct PAF for female homicides

The burden of homicides attributable to intimate partner violence was modelled as a direct PAF. Input data fed into a single-parameter proportion DisMod-MR 2.1 model, which had age mesh points at 0, 10, 20, 45, and 100. The model had a study-level covariate for sources just including police reported homicides. A study-level fixed effect on integrand variance (z-cov) was also included to indicate whether a study was nationally representative or not. This covariate was first tested as an x-cov and the coefficient indicated no systematic bias.

Cumulative risk approach for PAF of HIV/AIDS due to IPV

The third and final modelling approach that was used to assess burden attributable to intimate partner violence was a cumulative risk approach to measure the burden of HIV/AIDS attributable to IPV. From two cohort studies (Jewkes et al, Lancet 2010 & Kouyoumdjijan, et al AIDS 2013) incidence rate ratio (IRR) of HIV incidence was pooled with a random effects model. As the burden is measured based on deaths and prevalence, attributable fractions for prevalence and death were quantified rather than incidence. To get a PAF for prevalence, the history of exposure to IPV and the accumulated associated risk of incident HIV due to IPV was considered, relative to the overall risk of HIV at the population level. The ratio of cumulative IPV attributable HIV incidence to total HIV incidence was an approximation of the relevant PAF for HIV prevalence and GBD assumed this PAF can also be applied to mortality.

$$\frac{\textit{Cumulative HIV incidence due to IPV}}{\textit{Cumulative HIV incidence overall}} = \frac{1-\prod_{a=o}^{a=n}(1-PAF_{ay} * I_{ay})}{1-\prod_{a=o}^{a=n}(1-I_{ay})}$$

Where: I = annual incidence rate of HIV, a = age (15-95), y = year (1980-2017)

$$PAF_{\textit{HIV incidence}} = \frac{[\textit{Prevalence of IPV}]_{ay} * (IRR-1)}{[\textit{Prevalence of IPV}]_{ay} * (IRR-1)+1}$$

Theoretical minimum-risk exposure level

The theoretical minimum-risk exposure level is zero exposure to intimate partner violence, as defined above.

Relative risks

The burden attributable to IPV was estimated for abortion, depressive disorders, interpersonal violence (i.e. homicide) and HIV incidence.

For HIV, a pooled IRR of 1.59 (95% CI 1.3-1.94) from a random effects inverse variance weighted meta-analysis of the two available prospective studies as of date was used.

The relative risks for depressive disorders and suicide came from a systematic review of longitudinal studies assessing intimate partner violence and incident diagnosed major depression. A random effects inverse variance weighted meta-analysis produced a pooled relative risk and 95% confidence interval of 1.44 (1.09-1.92).

F.4. Lead exposure

The steps in the estimation of lead exposure are shown in the following flowchart:

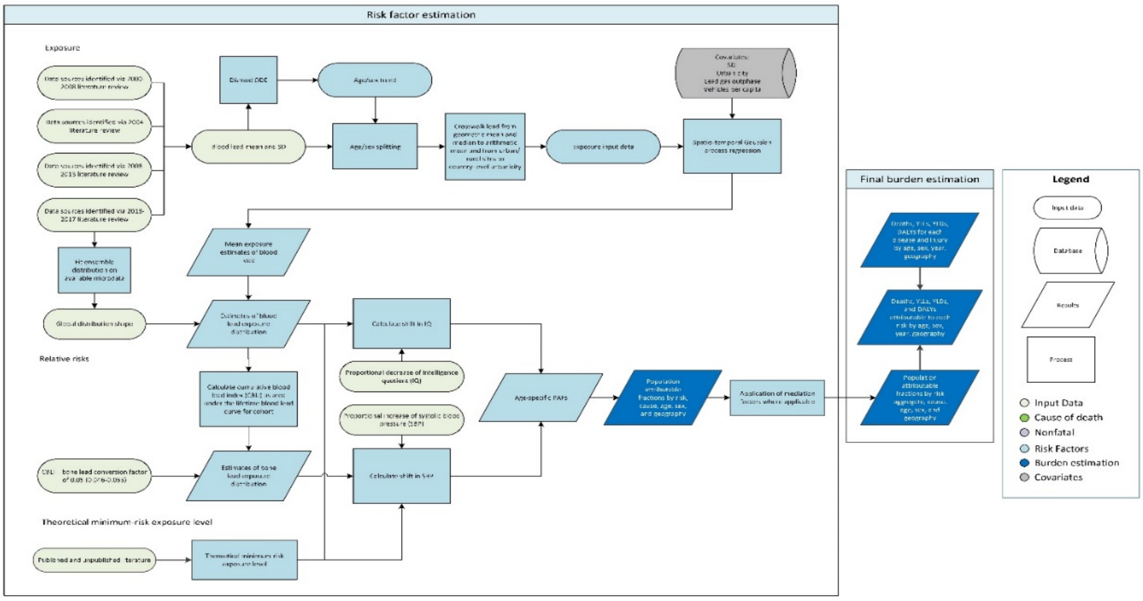

Data

The input data for lead exposure is primarily extracted from global literature on blood lead. In addition to that, few blood lead surveys were also used. Blood lead values are derived from studies that take blood samples and analysed them using various techniques to determine the level of lead present.

Modelling strategy

The spatio-temporal Gaussian process regression modelling methodology was used for the estimation of lead exposure. In order to predict blood lead in country-years with insufficient data, covariates that have been produced across time and space relevant to this analysis were used. For blood lead exposure, the covariates determined to have predictive ability were the Socio-demographic Index (SDI), the proportion of a location's population living in urban settings (logit transformed), the combined number of 2 and 4 wheel vehicles per capita, and a covariate indicating whether leaded gasoline had been phased out in a given country-year (smoothed over the first 5 years of phase-out to reflect its gradual implementation). ST-GPR was used to produce estimates of mean and standard deviation of blood lead for all age groups, for both sexes, and for all GBD locations from 1970 to 2017.

To calculate blood lead over the lifetime of a given cohort, blood lead was assumed to grow linearly from 2.0 ug/dL in 1920 (see Theoretical minimum-risk exposure level (TMREL)) to the value for that cohort in 1970. Using the exposure distributions of blood lead over time and space, cohorts were constructed such that lifetime blood lead could be expressed as a curve over each year of life. The area under this curve was the cumulative blood lead index, which could be used to estimate bone lead in a given year with the aforementioned scalar.

Outcomes were included based on the strength of available evidence supporting a causal relationship. Blood lead level (a measure of acute lead exposure) is paired with idiopathic developmental intellectual disability as modelled through the impact of blood lead levels on IQ in children. Bone lead level (a measure of cumulative lead exposure) is paired with systolic blood pressure as an outcome, and subsequently to all cardiovascular outcomes to which systolic blood pressure is paired, which includes rheumatic heart disease, ischemic heart disease, ischemic stroke, intracerebral haemorrhage, hypertensive heart disease, other cardiomyopathy, atrial fibrillation and flutter, aortic aneurysm, peripheral artery disease, endocarditis, other cardiovascular and circulatory diseases, chronic kidney disease due to hypertension, chronic kidney disease due to glomerulonephritis, and chronic kidney disease due to other and unspecified causes.

Theoretical minimum-risk exposure level

In previous iterations of GBD, the TMREL was taken from literature estimates of pre-industrial blood lead in humans. That value was estimated at 2.0 ug/dL. The decision was made that the TMREL of blood lead could not be 0 given the ambient sources of lead that would be impossible to eliminate. However, average blood lead exposures in a number of countries have fallen below 2.0 ug/dL in the past few years, suggesting that the TMREL ought to be lowered. Unfortunately, literature with statistically significant estimates for relative risk at such low levels of blood lead exposure could not be found. As a result, a TMREL of 2.0 ug/dL was used.

Relative Risks

Because the relative risk of IQ loss from lead exposure is specific to children, estimates of a cohort's lead exposure in early childhood (at 24 months of age) were used to determine past IQ loss, and thus calculate burden via the impact on concurrent IQ in the older population.

Population Attributable Fraction

The standard GBD population attributable fraction (PAF) equation were used to calculate PAFs for bone lead exposure and each of its paired outcomes using exposure estimates and relative risks. A similar approach for estimating PAFs was used for the burden of intellectual disability attributable to blood lead, which uses the estimated distribution of intellectual disability and the modelled shifts in IQ due to blood lead levels to determine the PAF.

G. Uncertainty intervals

Point estimates for each quantity of interest were derived from the mean of the draws, while 95% uncertainty intervals (UIs) were derived from the 2.5th and 97.5th percentiles of the 1,000 draw level values. Uncertainty in the estimation is attributable to sample size variability within data sources, different availability of data by age, sex, year, or location, and cause-specific model specifications. The UIs were determined for components of cause-specific estimation based on 1,000 draws from the posterior distribution of cause specific mortality by age, sex, and location for each year included in the GBD 2017 analysis. Similarly, for non-fatal estimates if there was a change in disease estimates between locations or over time that was in the same direction in more than 950 of the 1,000 samples we report it as significant. With this approach, uncertainty could be quantified and propagated into the final quantities of interest.

2. GBD 2017 India data inputs for mental disorders burden, risk factors and covariates

Abel R, Sampathkumar V. Tamil Nadu nutritional survey comparing children aged 0-3 years with the NCHS/CDC reference population. Indian J Pediatr. 1998; 65: 565-72.
Ahmad A, Khalique N, Khan Z, Amir A. Prevalence of psychosocial problems among school going male adolescents. Indian J Community Med. 2007; 32: 219-21.
Ajinkya S, Kaur D, Gursale A, Jadhav P. Prevalence of parent-rated attention deficit hyperactivity disorder and associated parent-related factors in primary school children of Navi Mumbai--a school based study. Indian J Pediatr. 2013; 80: 207–10.
Anita, Gaur DR, Vohra AK, Subhash S, Khurana H. Prevalence of psychiatric morbidity among 6 to 14 Years old children. Indian J Community Med. 2003; 28: 133-7.
Awasthi S, Das R, Verma T, Vir S. Anaemia and undernutrition among preschool children in Uttar Pradesh, India. Indian Pediatr. 2003; 40: 985-90.
Bansal PD, Barman R. Psychopathology of school going children in the age group of 10-15 years. Int J Appl Basic Med Res. 2011; 1: 43-7.
Behera P, Sharan P, Mishra AK, Nongkynrih B, Kant S, Gupta SK. Prevalence and determinants of depression among elderly persons in a rural community from northern India. Natl Med J India. 2016; 29: 129–35.
Bowes L, Joinson C, Wolke D, Lewis G. Peer victimisation during adolescence and its impact on depression in early adulthood: prospective cohort study in the United Kingdom. BMJ. 2015; 350: h2469.
Bromet E, Andrade LH, Hwang I, Sampson NA, Alonso J, de Girolamo G, et al. Cross-national epidemiology of DSM-IV major depressive episode. BMC Med. 2011; 9: 90.
Brown J, Cohen P, Johnson JG, Smailes EM. Childhood abuse and neglect: specificity of effects on adolescent and young adult depression and suicidality. J Am Acad Child Adolesc Psychiatry. 1999; 38: 1490–6.
Central Board of Secondary Education, Department of School Education and Literacy, Ministry of Human Resource Development, Government of India, Centres for Disease Control and Prevention, World Health Organisation (WHO). India Global School-Based Student Health Survey 2007. Geneva, Switzerland: WHO; 2007.
Centre for Vaccine Development, University of Maryland, Centres for Disease Control and Prevention, Department of Medical Microbiology and Immunology, Göteborg University, International Vaccine Institute, National Institute of Cholera and Enteric Diseases, Perry Point Cooperative Studies Program Coordinating Centre, U.S. Department of Veterans Affairs, School of Medicine, University of Virginia, University of Chile. India - Kolkata Global Enteric Multicentre Study 2011-2013. Baltimore, United States: Centre for Vaccine Development, University of Maryland.
Chapman DP, Whitfield CL, Felitti VJ, Dube SR, Edwards VJ, Anda RF. Adverse childhood experiences and the risk of depressive disorders in adulthood. Journal of affective disorders. 2004; 82: 217-225.
Cheasty M, Clare AW., Collins C. Relation between sexual abuse in childhood and adult depression: case-control study. BMJ. 1998; 316: 198-201.
Chowdhary N, Patel V. The effect of spousal violence on women's health: findings from the Stree Arogya Shodh in Goa, India. J Postgrad Med. 2008; 54: 306–12.
Christian Medical College - Vellore, MRC Epidemiology Resource Centre, University of Southampton. India - Vellore Birth Cohort Study 1998-2002. [Data shared for this analysis]
Dave U, Shetty N, Mehta L. A community genetics approach to population screening in India for mental retardation – a model for developing countries. Ann Hum Biol. 2005; 32: 195-203.
Deivasigamani TR. Psychiatric morbidity in primary school children – an epidemiological study. Indian J Psychiatry. 1990; 32: 235-40.
Department of Women and Child Development, Ministry of Human Resource Development, Government of India, United Nations Children's Fund (UNICEF) - India Country Office. India Summary Report on the Multiple Indicator Cluster Survey 2000. New Delhi, India: UNICEF India Country Office; 2000.
Department of Women and Child Development, Ministry of Human Resource Development, Government of India. India Nutrition Profile Survey 1995-1996. New Delhi, India: Ministry of Human Resource Development.
Dinwiddie S, Heath AC, Dunne MP, et al. Early sexual abuse and lifetime psychopathology: a co-twin-control study. Psychol Med. 2000; 30: 41–52.
Dube SR, Anda RF, Whitfield CL, Brown DW, Felitti VJ, Dong M, Giles WH. Longterm consequences of childhood sexual abuse by gender of victim. American journal of preventive medicine. 2005; 28: 430-438.
Ernst C, Angst J, Földényi M. The Zurich Study. XVII. Sexual abuse in childhood. Frequency and relevance for adult morbidity data of a longitudinal epidemiological study. Eur Arch Psychiatry Clin Neurosci. 1993; 242: 293–300.
Fahy AE, Stansfeld SA, Smuk M, Smith NR, Cummins S, Clark C. Longitudinal associations between cyberbullying involvement and adolescent mental health. Adolesc Health. 2016; 59: 502–9.
Farrington DP, Loeber R, Stallings R, Ttofi MM. Bullying perpetration and victimization as predictors of delinquency and depression in the Pittsburgh Youth Study. Journal of Aggression Conflict and Peace Research. 2011;3: 74–81.
Fekkes M, Pijpers FIM, Fredriks AM, Vogels T, Verloove-Vanhorick SP. Do bullied children get ill, or do ill children get bullied? A prospective cohort study on the relationship between bullying and health-related symptoms. Pediatrics. 2006; 117: 1568–74
Food and Agriculture Organisation of the United Nations (FAO). FAOSTAT Commodity Balances - Crops Primary Equivalent. Rome, Italy: FAO.
Food and Agriculture Organisation of the United Nations (FAO). FAOSTAT Food Balance Sheets, October 2014. Rome, Italy: FAO.
Gibb SJ, Horwood LJ, Fergusson DM. Bullying victimization/perpetration in childhood and later adjustment: findings from a 30 year longitudinal study. Jnl Aggress Conflict Peace Res. 2011; 3: 82–8.

Global Burden of Disease Health Financing Collaborator Network, Institute for Health Metrics and Evaluation (IHME). Global Development Assistance for Health, Government, Prepaid Private, and Out-of-Pocket Health Spending 1995-2014. Seattle, United States: IHME; 2017.

Guerra M, Prina AM, Ferri CP, Acosta D, Gallardo S, Huang Y, Jacob KS, Jimenez-Velazquez IZ, Llibre Rodriguez JJ, Liu Z, Salas A, Sosa AL, Williams JD, Uwakwe R, Prince M. A comparative cross-cultural study of the prevalence of late life depression in low and middle income countries. J Affect Disord. 2016; 190: 362–8.

Gururaj G, Varghese M, Benegal V, et al. National Mental Health Survey Report, 2015-16: prevalence, patterns and outcomes. Bengaluru: National Institute of Mental Health and Neuro Sciences, 2016.

Hackett R, Hackett, Latha, Bhakta, Preeta, Gowers, Simon. The Prevalence and Associations of Psychiatric Disorder in Children in Kerala, South India. J Child Psychol Psychiatry. 1999; 40: 801-7.

Harrison G, Hopper K, Craig T, Laska E, Siegel C, Wanderling J, Dube KC, Ganev K, Giel R, an der Heiden W, Holmberg SK, Janca A, Lee PW, León CA, Malhotra S, Marsella AJ, Nakane Y, Sartorius N, Shen Y, Skoda C, Thara R, Tsirkin SJ, Varma VK, Walsh D, Wiersma D. Recovery from psychotic illness: a 15- and 25-year international follow-up study. Br J Psychiatry. 2001; 178: 506-17.

Health Management Information System (HMIS), National Health Mission, Ministry of Health and Family Welfare, Government of India. India Performance Related to Immunisation 2007-2009. New Delhi, India: Ministry of Health and Family Welfare.

Health Management Information System (HMIS), National Health Mission, Ministry of Health and Family Welfare, Government of India. India Performance Related to Immunisation 2008-2010. New Delhi, India: Ministry of Health and Family Welfare.

Health Management Information System (HMIS), National Health Mission, Ministry of Health and Family Welfare, Government of India. India Performance Related to Immunisation 2009-2011. New Delhi, India: Ministry of Health and Family Welfare.

Health Management Information System (HMIS), National Health Mission, Ministry of Health and Family Welfare, Government of India. India Performance Related to Immunisation 2010-2012. New Delhi, India: Ministry of Health and Family Welfare.

Health Management Information System (HMIS), National Health Mission, Ministry of Health and Family Welfare, Government of India. India Performance Related to Immunisation 2011-2013. New Delhi, India: Ministry of Health and Family Welfare.

Health Management Information System (HMIS), National Health Mission, Ministry of Health and Family Welfare, Government of India. India Performance Related to Immunisation 2012-2014. New Delhi, India: Ministry of Health and Family Welfare.

Health Management Information System (HMIS), National Health Mission, Ministry of Health and Family Welfare, Government of India. India Performance Related to Immunisation 2013-2015. New Delhi, India: Ministry of Health and Family Welfare.

Health Management Information System (HMIS), National Health Mission, Ministry of Health and Family Welfare, Government of India. India Performance Related to Immunisation 2014-2016. New Delhi, India: Ministry of Health and Family Welfare.

Health Management Information System (HMIS), National Health Mission, Ministry of Health and Family Welfare, Government of India. India Performance Related to Immunisation 2015-2017. New Delhi, India: Ministry of Health and Family Welfare.

Hemphill SA, Kotevski A, Heerde JA. Longitudinal associations between cyber-bullying perpetration and victimization and problem behavior and mental health problems in young Australians. Int J Public Health. 2015; 60: 227–37.

Hemphill SA, Kotevski A, Herrenkohl TI, Bond L, Kim MJ, Toumbourou JW, Catalano RF. Longitudinal consequences of adolescent bullying perpetration and victimisation: A study of students in Victoria, Australia. Crim Behav Ment Health. 2011; 21: 107–16.

Institute of Health Systems, World Health Organisation (WHO). WHO Multi-country Survey Study Report on Health and Health System Responsiveness, Andhra Pradesh 2000-2001.

International Institute for Population Sciences (IIPS), Ministry of Health and Family Welfare, Government of India, Harvard T. H. Chan School of Public Health, RAND Corporation, Monash University, University of California Los Angeles, Indian Academy of Geriatrics, National AIDS Research Institute, Columbia University. Longitudinal Aging Study in India, Pilot Data 2010. Mumbai, India: IIPS.

International Institute for Population Sciences (IIPS), Ministry of Health and Family Welfare, Government of India, ICF. India National Family Health Survey (NFHS-4) 2015-2016: National and State-level Factsheets. Mumbai, India: IIPS.

International Institute for Population Sciences (IIPS), Ministry of Health and Family Welfare, Government of India, Macro International. India National Family Health Survey (NFHS-1) 1992-1993: National Report. Mumbai, India: IIPS.

International Institute for Population Sciences (IIPS), Ministry of Health and Family Welfare, Government of India, Macro International. India National Family Health Survey (NFHS-3) 2005-2006: National and State Reports. Mumbai, India: IIPS.

International Institute for Population Sciences (IIPS), Ministry of Health and Family Welfare, Government of India, Macro International. India National Family Health Survey Data (NFHS-1) 1992-1993. Mumbai, India: IIPS.

International Institute for Population Sciences (IIPS), Ministry of Health and Family Welfare, Government of India, Macro International. India National Family Health Survey Data (NFHS-3) 2005-2006. Mumbai, India: IIPS.

International Institute for Population Sciences (IIPS), Ministry of Health and Family Welfare, Government of India, ORC Macro. India National Family Health Survey (NFHS-2) 1998-1999: National and State Reports. Mumbai, India: IIPS.

International Institute for Population Sciences (IIPS), Ministry of Health and Family Welfare, Government of India, ORC Macro. India National Family Health Survey Data (NFHS-2) 1998-1999. Mumbai, India: IIPS.

International Institute for Population Sciences (IIPS), Ministry of Health and Family Welfare, Government of India. India District Level Household and Facility Survey (DLHS-3) 2007-2008: National and State Reports. Mumbai, India: IIPS.

International Institute for Population Sciences (IIPS), Ministry of Health and Family Welfare, Government of India. India District Level Household and Facility Survey Data (DLHS-3) 2007-2008. Mumbai, India: IIPS.

International Institute for Population Sciences (IIPS), Ministry of Health and Family Welfare, Government of India. India District Level Household and Facility Survey Data (DLHS-4) 2012-2013: State Reports. Mumbai, India: IIPS.

International Institute for Population Sciences (IIPS), Ministry of Health and Family Welfare, Government of India. India District Level Household Survey (DLHS-1) 1998-1999: National Report. Mumbai, India: IIPS.

International Institute for Population Sciences (IIPS), Ministry of Health and Family Welfare, Government of India. India District Level Household Survey Data (DLHS-1) 1998-1999. Mumbai, India: IIPS.

International Institute for Population Sciences, Ministry of Health and Family Welfare, Government of India. India District Level Household and Facility Survey Data (DLHS-4) 2012-2013. Mumbai, India: IIPS.

International Institute for Population Sciences, World Health Organisation (WHO). India WHO Study on Global Ageing and Adult Health 2007-2008.

International Institute for Population Sciences, World Health Organisation. India World Health Survey 2003.

Jaffee SR, Moffitt TE, Caspi A, Fombonne E, Poulton R, Martin J. Differences in early childhood risk factors for juvenile-onset and adult-onset depression. Arch Gen Psychiatry. 2002; 59: 215-22.

Joshi K, Kumar R, Avasthi A. Morbidity profile and its relationship with disability and psychological distress among elderly people in northern India. Int J Epidemiol. 2003; 32: 978-87.

Kaltiala-Heino R, Frojd S, Marttunen M. Involvement in bullying and depression in a 2-year follow-up in middle adolescence. Eur Child Adolesc Psychiatry. 2010; 19: 45–55.

Kendler KS, Bulik CM, Silberg J, Hettema JM, Myers J, Prescott CA. Childhood sexual abuse and adult psychiatric and substance use disorders in women: an epidemiological and cotwin control analysis. Arch Gen Psychiatry. 2000; 57: 953–9.

Kessler RC, Birnbaum HG, Shahly V, Bromet E, Hwang I, McLaughlin KA, Sampson N, Andrade LH, de Girolamo G, Demyttenaere K, Haro JM, Karam AN, Kostyuchenko S, Kovess V, Lara C, Levinson D, Matschinger H, Nakane Y, Browne MO, Ormel J, Posada-Villa J, Sagar R, Stein DJ. Age differences in the prevalence and co-morbidity of DSM-IV major depressive episodes: results from the WHO World Mental Health Survey Initiative. Depress Anxiety. 2010; 27: 351–64.

Kodkany BS, Derman RJ, Honnungar NV, Tyagi NK, Goudar SS, Mastiholi SC, Moore JL, McClure EM, Sloan N, Goldenberg RL. Establishment of a maternal newborn health registry in the Belgaum district of Karnataka, India. Reprod Health. 2015; 12 Suppl 2: S3.

Kumpulainen K, Rasanen E. Children involved in bullying at elementary school age: their psychiatric symptoms and deviance in adolescence: An epidemiological sample. Child Abuse Negl. 2000; 24: 1567–77.

Lanphear BP, Hornung R, Khoury J, et al. Low-level environmental lead exposure and children’s intellectual function: an international pooled analysis. Environ Health Perspect 2005; 113: 894–9.

Lereya ST, Copeland WE, Zammit S, Wolke D. Bully/victims: a longitudinal, population-based cohort study of their mental health. Eur Child Adolesc Psychiatry. 2015; 24: 1461–71.

Lipsky S, Caetano R, Roy-Byrne P. Racial and ethnic disparities in police-reported intimate partner violence and risk of hospitalization among women. Womens Health Issues. 2009; 19:109–118.

Liu J, Li L, Wang Y, Yan C, Liu X. Impact of low blood lead concentrations on IQ and school performance in Chinese children. PLoS One. 2013;8: e65230

Malhotra S, Kohli A, Arun P. Prevalence of psychiatric disorders in school children in Chandigarh, India. Indian J Med Res. 2002; 116: 21-8.

Mathias K, Goicolea I, Kermode M, Singh L, Shidhaye R, Sebastian MS. Cross-sectional study of depression and help-seeking in Uttarakhand, north India. BMJ Open. 2015; 5: e008992.

Mehta P, Joseph A, Verghese A. An epidemiologic study of psychiatric disorders in a rural area in Tamilnadu. Indian J Psychiatry. 1985; 27: 153-8.

Merikangas KR, Jin R, He J-P, Kessler RC, Lee S, Sampson NA, Viana MC, Andrade LH, Hu C, Karam EG, Ladea M, Medina-Mora ME, Ono Y, Posada-Villa J, Sagar R, Wells JE, Zarkov Z. Prevalence and correlates of bipolar spectrum disorder in the World Mental Health Survey Initiative. Arch Gen Psychiatry. 2011; 68: 241-51.

Ministry of Health and Family Welfare, Government of India, United Nations Children's Fund. India Coverage Evaluation Survey 2005.

Ministry of Health and Family Welfare, Government of India, United Nations Children's Fund. India Coverage Evaluation Survey 2007.

Ministry of Health and Family Welfare, Government of India, United Nations Children's Fund. India Coverage Evaluation Survey Data 2009-2010. [Data shared for this analysis]

Ministry of Statistics and Programme Implementation, Government of India. Common Property Resources, Sanitation & Hygiene Services, National Sample Survey Round 54, January - June 1998. New Delhi, India: Ministry of Statistics and Programme Implementation.

Ministry of Statistics and Programme Implementation, Government of India. Consumer Expenditure Survey, India National Sample Survey Round 55, July 1999-June 2000. New Delhi, India: Ministry of Statistics and Programme Implementation.

Ministry of Statistics and Programme Implementation, Government of India. Consumer Expenditure Survey, National Sample Survey Round 38, January-December 1983. New Delhi, India: Ministry of Statistics and Programme Implementation.

Ministry of Statistics and Programme Implementation, Government of India. Consumer Expenditure Survey, National Sample Survey Round 43, July 1987-June 1988. New Delhi, India: Ministry of Statistics and Programme Implementation.

Ministry of Statistics and Programme Implementation, Government of India. Consumer Expenditure Survey, National Sample Survey Round 47, July - December 1991. New Delhi, India: Ministry of Statistics and Programme Implementation.

Ministry of Statistics and Programme Implementation, Government of India. Consumer Expenditure Survey, National Sample Survey Round 49, January-June 1993. New Delhi, India: Ministry of Statistics and Programme Implementation.

Ministry of Statistics and Programme Implementation, Government of India. Consumer Expenditure Survey, National Sample Survey Round 50, July 1993-June 1994. New Delhi, India: Ministry of Statistics and Programme Implementation.

Ministry of Statistics and Programme Implementation, Government of India. Consumer Expenditure Survey, National Sample Survey Round 50, July 1994-June 1995. New Delhi, India: Ministry of Statistics and Programme Implementation.
Ministry of Statistics and Programme Implementation, Government of India. Consumer Expenditure Survey, National Sample Survey Round 52, July 1995-June 1996. New Delhi, India: Ministry of Statistics and Programme Implementation.
Ministry of Statistics and Programme Implementation, Government of India. Consumer Expenditure Survey, National Sample Survey Round 54, January - June 1998. New Delhi, India: Ministry of Statistics and Programme Implementation.
Ministry of Statistics and Programme Implementation, Government of India. Consumer Expenditure Survey, National Sample Survey Round 60, January-June 2004. New Delhi, India: Ministry of Statistics and Programme Implementation.
Ministry of Statistics and Programme Implementation, Government of India. Consumer Expenditure Survey, National Sample Survey Round 61, July 2004 - June 2005. New Delhi, India: Ministry of Statistics and Programme Implementation.
Ministry of Statistics and Programme Implementation, Government of India. Consumer Expenditure Survey, National Sample Survey Round 64, July 2007- June 2008. New Delhi, India: Ministry of Statistics and Programme Implementation.
Ministry of Statistics and Programme Implementation, Government of India. Employment and Unemployment Survey, India National Sample Survey Round 55, July 1999-June 2000. New Delhi, India: Ministry of Statistics and Programme Implementation.
Ministry of Statistics and Programme Implementation, Government of India. Employment and Unemployment Survey, National Sample Survey Round 38, January-December 1983. New Delhi, India: Ministry of Statistics and Programme Implementation.
Ministry of Statistics and Programme Implementation, Government of India. Employment and Unemployment Survey, National Sample Survey Round 43, July 1987- June 1988. New Delhi, India: Ministry of Statistics and Programme Implementation.
Ministry of Statistics and Programme Implementation, Government of India. Employment and Unemployment Survey, National Sample Survey Round 50, July 1993- June 1994. New Delhi, India: Ministry of Statistics and Programme Implementation.
Ministry of Statistics and Programme Implementation, Government of India. Employment and Unemployment Survey, National Sample Survey Round 50, July 1994-June 1995. New Delhi, India: Ministry of Statistics and Programme Implementation.
Ministry of Statistics and Programme Implementation, Government of India. Employment and Unemployment Survey, National Sample Survey Round 61, July 2004- June 2005. New Delhi, India: Ministry of Statistics and Programme Implementation.
Ministry of Statistics and Programme Implementation, Government of India. Employment and Unemployment Survey, National Sample Survey Round 64, July 2007- June 2008. New Delhi, India: Ministry of Statistics and Programme Implementation.
Ministry of Statistics and Programme Implementation, Government of India. Employment and Unemployment surveys, National Sample Survey Round 60, January-June 2004. New Delhi, India: Ministry of Statistics and Programme Implementation.
Ministry of Statistics and Programme Implementation, Government of India. Household expenditure on services and durable goods survey, National Sample Survey Round 72, July 2014 - June 2015. New Delhi, India: Ministry of Statistics and Programme Implementation.
Ministry of Statistics and Programme Implementation, Government of India. India State Series: Gross State Domestic Product at Factor Cost by Industry of Origin Tables 1980-81 to 1993-94. New Delhi, India: Ministry of Statistics and Programme Implementation.
Ministry of Statistics and Programme Implementation, Government of India. India State Series: Gross State Domestic Product at Factor Cost by Industry of Origin Tables 1993-94 to 2004-05. New Delhi, India: Ministry of Statistics and Programme Implementation.
Ministry of Statistics and Programme Implementation, Government of India. India State Series: Gross State Domestic Product at Factor Cost by Industry of Origin Tables 1999-2000 to 2009-10. New Delhi, India: Ministry of Statistics and Programme Implementation.
Ministry of Statistics and Programme Implementation, Government of India. India State Series: Gross State Domestic Product at Factor Cost by Industry of Origin Tables 2004-05 to 2013-14. New Delhi, India: Ministry of Statistics and Programme Implementation.
Ministry of Statistics and Programme Implementation, Government of India. Participation & Expenditure in Education Survey, National Sample Survey Round 64, July 2007- June 2008. New Delhi, India: Ministry of Statistics and Programme Implementation.
Ministry of Statistics and Programme Implementation, Government of India. Participation in Education Survey, National Sample Survey Round 52, July 1995-June 1996. New Delhi, India: Ministry of Statistics and Programme Implementation.
Ministry of Statistics and Programme Implementation, Government of India. Survey on Healthcare, National Sample Survey Round 52, July 1995-June 1996. New Delhi, India: Ministry of Statistics and Programme Implementation.
Ministry of Statistics and Programme Implementation, Government of India. Survey on Literacy and Culture, National Sample Survey Round 47, July - December 1991. New Delhi, India: Ministry of Statistics and Programme Implementation.
Ministry of Statistics and Programme Implementation, Government of India. Survey on Morbidity and Healthcare, National Sample Survey Round 60, January-June 2004. New Delhi, India: Ministry of Statistics and Programme Implementation.
Ministry of Statistics and Programme Implementation, Government of India. Survey on Participation in Education, National Sample Survey Round 42, July 1986- June 1987. New Delhi, India: Ministry of Statistics and Programme Implementation.
Ministry of Women and Child Development, Government of India, United Nations Children's Fund. India Rapid Survey on Children 2013-2014. New Delhi, India: Ministry of Women and Child Development.
Molnar BE, Buka SL, Kessler RC. Child sexual abuse and subsequent psychopathology: results from the National Comorbidity Survey. American journal of public health. 2001; 91: 753.
Moore SE, Norman RE, Sly PD, Whitehouse AJO, Zubrick SR, Scott J. Adolescent peer aggression and its association with mental health and substance use in an Australian cohort. J Adolesc. 2014; 37: 11–21
Morgan C, John S, Esan O, Hibben M, Patel V, Weiss HA, Murray R, Hutchinson G, Gureje O, Thara R, Cohen A. The incidence of psychoses in diverse settings, INTREPID (2): a feasibility study in India, Nigeria, and Trinidad. Psychol Med. 2016; 46: 1923-33.
Nair MKC, Russell PSS, Mammen P, Abhiram Chandran R, Krishnan R, Nazeema S, Chembagam N, Peter D. ADad 3: the epidemiology of anxiety disorders among adolescents in a rural community population in India. Indian J Pediatr. 2013; 80 Suppl 2: S144-148.

National Institute of Medical Statistics, Indian Council of Medical Research (ICMR), Integrated Disease Surveillance Programme. Non-Communicable Disease Risk Factors Survey Data 2007-2008. New Delhi, India: ICMR. [Data shared for this analysis]
National Institute of Medical Statistics, Indian Council of Medical Research (ICMR), Integrated Disease Surveillance Programme. Non-Communicable Disease Risk Factors Survey Report 2007-2008. New Delhi, India: ICMR.
National Institute of Mental Health and Neuro Sciences. National Mental Health Survey Data Tables, 2015-16. [Data shared for this analysis]
National Nutrition Monitoring Bureau, National Institute of Nutrition (NIN), Indian Council of Medical Research. India Rural First Repeat Survey of Diet and Nutritional Status Data 1988-1990. [Data shared for this analysis]
National Nutrition Monitoring Bureau, National Institute of Nutrition (NIN), Indian Council of Medical Research. India Rural First Repeat Survey of Diet and Nutritional Status Report 1988-1990. Hyderabad, India: NIN.
National Nutrition Monitoring Bureau, National Institute of Nutrition (NIN), Indian Council of Medical Research. India Rural Second Repeat Survey of Diet and Nutritional Status Data 1996-1997. [Data shared for this analysis]
National Nutrition Monitoring Bureau, National Institute of Nutrition (NIN), Indian Council of Medical Research. India Rural Second Repeat Survey of Diet and Nutritional Status Report 1996-1997. Hyderabad, India: NIN.
National Nutrition Monitoring Bureau, National Institute of Nutrition (NIN), Indian Council of Medical Research. India Rural Survey of Diet and Nutritional Status Data 1994-1995. [Data shared for this analysis]
National Nutrition Monitoring Bureau, National Institute of Nutrition (NIN), Indian Council of Medical Research. India Rural Survey of Diet and Nutritional Status Data 2000-2001. [Data shared for this analysis]
National Nutrition Monitoring Bureau, National Institute of Nutrition (NIN), Indian Council of Medical Research. India Rural Survey of Diet and Nutritional Status Data 2004-2006. [Data shared for this analysis]
National Nutrition Monitoring Bureau, National Institute of Nutrition (NIN), Indian Council of Medical Research. India Rural Survey of Diet and Nutritional Status Report 1994-1995. Hyderabad, India: NIN.
National Nutrition Monitoring Bureau, National Institute of Nutrition (NIN), Indian Council of Medical Research. India Rural Survey of Diet and Nutritional Status Report 2000-2001. Hyderabad, India: NIN.
National Nutrition Monitoring Bureau, National Institute of Nutrition (NIN), Indian Council of Medical Research. India Rural Survey of Diet and Nutritional Status Report 2004-2006. Hyderabad, India: NIN.
National Nutrition Monitoring Bureau, National Institute of Nutrition (NIN), Indian Council of Medical Research. India Rural Third Repeat Survey of Diet and Nutritional Status Data 2011-2012. [Data shared for this analysis]
National Nutrition Monitoring Bureau, National Institute of Nutrition (NIN), Indian Council of Medical Research. India Rural Third Repeat Survey of Diet and Nutritional Status Report 2011-2012. Hyderabad, India: NIN.
National Nutrition Monitoring Bureau, National Institute of Nutrition (NIN), Indian Council of Medical Research. India Survey of Diet and Nutritional Status Data 1990-1992. [Data shared for this analysis]
National Nutrition Monitoring Bureau, National Institute of Nutrition (NIN), Indian Council of Medical Research. India Survey of Diet and Nutritional Status Report 1990-1992. Hyderabad, India: NIN.
National Nutrition Monitoring Bureau, National Institute of Nutrition (NIN), Indian Council of Medical Research. India Tribal First Repeat Survey of Diet and Nutritional Status Data 1998-1999. [Data shared for this analysis]
National Nutrition Monitoring Bureau, National Institute of Nutrition (NIN), Indian Council of Medical Research. India Tribal First Repeat Survey of Diet and Nutritional Status Report 1998-1999. Hyderabad, India: NIN.
National Nutrition Monitoring Bureau, National Institute of Nutrition (NIN), Indian Council of Medical Research. India Tribal Second Repeat Survey of Diet and Nutritional Status Data 2007-2008. [Data shared for this analysis]
National Nutrition Monitoring Bureau, National Institute of Nutrition (NIN), Indian Council of Medical Research. India Tribal Second Repeat Survey of Diet and Nutritional Status Report 2007-2008. Hyderabad, India: NIN.
National Nutrition Monitoring Bureau, National Institute of Nutrition (NIN), Indian Council of Medical Research. India Urban Slums Survey of Diet and Nutritional Status Data 1993-1994. [Data shared for this analysis]
National Nutrition Monitoring Bureau, National Institute of Nutrition (NIN), Indian Council of Medical Research. India Urban Slums Survey of Diet and Nutritional Status Report 1993-1994. Hyderabad, India: NIN.
National Nutrition Monitoring Bureau, National Institute of Nutrition, Indian Council of Medical Research. India National Nutrition Monitoring Bureau Eight States Pooled Data 1991-1992. [Data shared for this analysis]
Nazareth Hospital. Inpatient and Discharge Data 2014. Shillong, Meghalaya: Nazareth Hospital. [Data shared for this analysis]
Needleman HL, Schell A, Bellinger D, Leviton A, Allred EN. The long-term effects of exposure to low doses of lead in childhood. New England Journal of Medicine. 1990;322: 83–8.
Nelson EC, Heath AC, Madden PA, Cooper ML, Dinwiddie SH, Bucholz KK, Glowinski A, McLaughlin T, Dunne MP, Statham DJ, Martin NG. Association between self-reported sexual abuse and adverse psychosocial outcomes: results from a twin study. Arch Gen Psychiatry. 2002; 59: 139-45.
Office of the Registrar General & Census Commissioner, Ministry of Home Affairs, Government of India. India Annual Health Survey Data 2010-2011. New Delhi, India: Office of the Registrar General & Census Commissioner.
Office of the Registrar General & Census Commissioner, Ministry of Home Affairs, Government of India. India Annual Health Survey Data 2010-2013. New Delhi, India: Office of the Registrar General & Census Commissioner.
Office of the Registrar General & Census Commissioner, Ministry of Home Affairs, Government of India. India Annual Health Survey Data 2011-2012. New Delhi, India: Office of the Registrar General & Census Commissioner.

Office of the Registrar General & Census Commissioner, Ministry of Home Affairs, Government of India. India Annual Health Survey Data 2012-2013. New Delhi, India: Office of the Registrar General & Census Commissioner.
Office of the Registrar General & Census Commissioner, Ministry of Home Affairs, Government of India. India Annual Health Survey Report 2010-2011. New Delhi, India: Office of the Registrar General & Census Commissioner.
Office of the Registrar General & Census Commissioner, Ministry of Home Affairs, Government of India. India Annual Health Survey Report 2010-2013. New Delhi, India: Office of the Registrar General & Census Commissioner.
Office of the Registrar General & Census Commissioner, Ministry of Home Affairs, Government of India. India Annual Health Survey Report 2011-2012. New Delhi, India: Office of the Registrar General & Census Commissioner.
Office of the Registrar General & Census Commissioner, Ministry of Home Affairs, Government of India. India Annual Health Survey Report 2012-2013. New Delhi, India: Office of the Registrar General & Census Commissioner.
Office of the Registrar General & Census Commissioner, Ministry of Home Affairs, Government of India. India Population and Housing Census 1981. New Delhi, India: Office of the Registrar General & Census Commissioner.
Office of the Registrar General & Census Commissioner, Ministry of Home Affairs, Government of India. India Population and Housing Census 2001. New Delhi, India: Office of the Registrar General & Census Commissioner.
Office of the Registrar General & Census Commissioner, Ministry of Home Affairs, Government of India. India Population and Housing Census 2011. New Delhi, India: Office of the Registrar General & Census Commissioner; 2012.
Office of the Registrar General and Census Commissioner, Ministry of Health and Family Welfare, Government of India, National Institute of Health and Family Welfare, Nutrition Foundation of India, National Institute of Nutrition, Indian Council of Medical Research. India Clinical, Anthropometric and Bio-chemical (CAB) Survey Data 2014 [Biomarker Component of Annual Health Survey]. New Delhi, India: Office of the Registrar General and Census Commissioner.
Office of the Registrar General and Census Commissioner, Ministry of Health and Family Welfare, Government of India, National Institute of Health and Family Welfare, Nutrition Foundation of India, National Institute of Nutrition, Indian Council of Medical Research. India Clinical, Anthropometric and Bio-chemical (CAB) Survey Report 2014 [Biomarker Component of Annual Health Survey]. New Delhi, India: Office of the Registrar General and Census Commissioner.
Ouellet-Morin I, Fisher HL, York-Smith M, Fincham-Campbell S, Moffitt TE, Arseneault L. Intimate partner violence and new-onset depression: a longitudinal study of women's childhood and adult histories of abuse. Depression and anxiety. 2015;32: 316-324.
Padmavathi R, Rajkumar S, Kumar N, Manoharan A, Kamath S. Prevalence of schizophrenia in an urban community in madras. Indian J Psychiatry. 1988; 30: 233-9.
Patil RN, Nagaonkar SN, Shah NB, Bhat TS. A Cross-sectional Study of Common Psychiatric Morbidity in Children Aged 5 to 14 Years in an Urban Slum. J Fam Med Prim Care. 2013; 2: 164-8.
Patton GC, Olsson C, Bond L, Toumbourou JW, Carlin JB, Hemphill SA, Catalano RF. Predicting female depression across puberty: a two-nation longitudinal study. J Am Acad Child Adolesc Psychiatry. 2008; 47: 1424–32.
Peleikis DE, Mykletun A, Dahl AA. The relative influence of childhood sexual abuse and other family background risk factors on adult adversities in female outpatients treated for anxiety disorders and depression. Child Abuse & Neglect. 2004;28: 61-76.
Pillai A, Patel V, Cardozo P, Goodman R, Weiss HA, Andrew G. Non-traditional lifestyles and prevalence of mental disorders in adolescents in Goa, India. Br J Psychiatry. 2008; 192: 45-51.
Poongothai S, Pradeepa R, Ganesan A, Mohan V. Prevalence of depression in a large urban South Indian population-the Chennai Urban Rural Epidemiology Study (CURES-70). PLoS One. 2009; 4: e7185.
Poovathinal SA, Anitha A, Thomas R, Kaniamattam M, Melempatt N, Anilkumar A, Meena M. Prevalence of autism spectrum disorders in a semiurban community in south India. Ann Epidemiol. 2016; 26: 663–665e8.
Premarajan KC, Danabalan M, Chandrasekar R, Srinivasa DK. Prevalence of psychiatry morbidity in an urban community of Pondicherry. Indian J Psychiatry. 1993; 35: 99-102.
Prina AM, Ferri CP, Guerra M, Brayne C, Prince M. Prevalence of anxiety and its correlates among older adults in Latin America, India and China: cross-cultural study. Br J Psychiatry J Ment Sci. 2011;199: 485–91.
Raina SK, Kashyap V, Bhardwaj AK, Kumar D, Chander V. Prevalence of autism spectrum disorders among children (1-10 years of age) - findings of a mid-term report from Northwest India. J Postgrad Med. 2015; 61: 243–6.
Rajkumar AP, Thangadurai P, Senthilkumar P, Gayathri K, Prince M, Jacob KS. Nature, prevalence and factors associated with depression among the elderly in a rural south Indian community. Int Psychogeriatr. 2009; 21: 372-8.
Rajkumar S, Padmavathi R, Thara R, Menon MS. Incidence of schizophrenia in an urban community in Madras. Indian J Psychiatry. 1993; 35: 18-21.
Rothon C, Head J, Klineberg E, Stansfeld S. Can social support protect bullied adolescents from adverse outcomes? A prospective study on the effects of bullying on the educational achievement and mental health of adolescents at secondary schools in East London. J Adolesc. 2011; 34: 579–88.
Roy A, Bellinger D, Hu H, Schwartz J, Ettinger AS, Wright RO, Bouchard M, Palaniappan K, Balakrishnan K. Lead exposure and behaviour among young children in Chennai, India. Environ Health Perspect. 2009; 117: 1607-11.
Rudra A, Belmonte MK, Soni PK, Banerjee S, Mukerji S, Chakrabarti B. Prevalence of autism spectrum disorder and autistic symptoms in a school-based cohort of children in Kolkata, India. Autism Res. 2017:1597–605.
Sachdeva JS, Singh S, Sidhu BS, Goyal RK, Singh J. An epidemiological study of psychiatric disorders in rural Faridkot (Punjab). Indian J Psychiatry. 1986; 28: 317-23.
Sarkar S, Sinha VK, Praharaj SK. Depressive disorders in school children of suburban India: an epidemiological study. Soc Psychiatry Psychiatr Epidemiol. 2012; 47: 783-8.
Sarkhel S, Sinha VK, Arora M, Desarkar P. Prevalence of conduct disorder in schoolchildren of Kanke. Indian J Psychiatry. 2006; 48: 159-64.

Sartorius N, Jablensky A, Korten A, Ernberg G, Anker M, Cooper JE, Day R. Early manifestations and first-contact incidence of schizophrenia in different cultures: a preliminary report on the initial evaluation phase of the WHO Collaborative Study on determinants of outcome of severe mental disorders. Psychol Med. 1986; 16: 909–28.
Sathyanarayana Rao TS, Darshan MS, Tandon A, Raman R, Karthik KN, Saraswathi N, Das K, Harsha GT, Krishna VST, Ashok NC. Suttur study: An epidemiological study of psychiatric disorders in south Indian rural population. Indian J Psychiatry. 2014; 56: 238–45.
Seby K, Chaudhury S, Chakraborty R. Prevalence of psychiatric and physical morbidity in an urban geriatric population. Indian J Psychiatry. 2011; 53: 121-7.
Sen B, Nandi DN, Mukherjee SP, Mishra DC, Banerjee G, Sarkar S. Psychiatric morbidity in an urban slum-dwelling community. Indian J Psychiatry. 1984; 26: 185–93.
Shaji S, Verghese A, Promodu K, George B, Shibu VP. Prevalence of priority psychiatric disorders in a rural area in Kerala. Indian J Psychiatry. 1995; 37: 91–6.
Sharma S, Singh MM. Prevalence of mental disorders: An epidemiological study in Goa. Indian J Psychiatry. 2001; 43: 118–26.
Shidhaye R, Gangale S, Patel V. Prevalence and treatment coverage for depression: a population-based survey in Vidarbha, India. Soc Psychiatry Psychiatr Epidemiol. 2016; 51: 993–1003.
Sigurdson JF, Undheim AM, Wallander JL, Lydersen S, Sund AM. The long-term effects of being bullied or a bully in adolescence on externalizing and internalizing mental health problems in adulthood. Child Adolesc Psychiatry Ment Health. 2015; 9: 1-13
Silberg JL, Copeland W, Linker J, Moore AA, Roberson-Nay R, York TP. Psychiatric outcomes of bullying victimization: a study of discordant monozygotic twins. Psychol Med. 2016; 46: 1875–83.
Silvanus V, Subramanian P. Epidemiological study of mental morbidity in an urban slum community in India for the development of a community mental health programme. Nepal Med Coll J. 2012; 14: 13-7.
Silverman AB, Reinherz H Z, Giaconia, RM. The long-term sequelae of child and adolescent abuse: a longitudinal community study. Child abuse & neglect.1996; 20: 709-723.
Sourander A, Jensen P, Ronning JA, et al. What is the early adulthood outcome of boys who bully or are bullied in childhood? The Finnish “From a Boy to a Man” study. Pediatrics. 2007; 120: 397–404.
Srinath S, Girimaji SC, Gururaj G, Seshadri S, Subbakrishna DK, Bhola P, Kumar N. Epidemiological study of child and adolescent psychiatric disorders in urban and rural areas of Bangalore, India. Indian J Med Res. 2005; 122: 67-79.
Stein Z, Belmont L, Durkin M. Mild mental retardation and severe mental retardation compared: experiences in eight less developed countries. Ups J Med Sci Suppl. 1987; 44: 89-96.
Suglia SF, Duarte CS, Sandel MT. Housing quality, housing instability, and maternal mental health. J Urban Health. 2011; 88: 1105–16
Suvarna BS, Kamath A. Prevalence of attention deficit disorder among preschool age children. Nepal Med Coll J. 2009; 11: 1-4.
Thara R. Twenty-year course of schizophrenia: The Madras Longitudinal Study. Can J Psychiatry. 2004; 49: 564-9.
Uppsala Conflict Data Program (UCDP), Department of Peace and Conflict Research, Uppsala University. UCDP Non-State Conflict Dataset, Version 2.5, 2016. Uppsala, Sweden: Uppsala University; 2013.
Uppsala Conflict Data Program (UCDP), Department of Peace and Conflict Research, Uppsala University. UCDP One-Sided Violence Dataset, Version 1.4, 2015. Uppsala, Sweden: Uppsala University; 2015.
Vas CJ, Pinto C, Panikker D, Noronha S, Deshpande N, Kulkarni L, Sachdeva S. Prevalence of dementia in an urban Indian Population. Int Psychogeriatr. 2001; 13: 439-50.
Vassallo S, Edwards B, Renda J, Olsson CA. Bullying in early adolescence and antisocial behavior and depression six years later: what are the protective factors? J Sch Violence. 2014; 13: 100–24.
Vijayaraghavan K, Rao DH. Diet & nutrition situation in rural India. Indian J Med Res. 1998; 108:243-53.
Wichstrøm L, Belsky J, Berg-Nielsen TS. Preschool predictors of childhood anxiety disorders: a prospective community study. Journal of Child Psychology and Psychiatry. 2013; 54: 1327–36.
Widom CS, DuMont K, Czaja SJ. A prospective investigation of major depressive disorder and comorbidity in abused and neglected children grown up. Archives of general psychiatry. 2007; 64: 49-56.
World Bank. Survey of living conditions 1997-1998, Uttar Pradesh and Bihar. Washington D.C., United States: World Bank.
World Health Organisation (WHO), United Nations Children's Fund (UNICEF). WHO and UNICEF reported administrative data for immunization coverage time series. Available from: http://www.who.int/immunization/monitoring_surveillance/data/en/
World Health Organisation (WHO). Global database on child growth and malnutrition - Historical. Geneva, Switzerland: WHO.
World Health Organisation (WHO). Mental illness in general health care: an international study. Geneva, Switzerland: WHO; 1995.
World Health Organisation (WHO). WHO World Mental Health Surveys: global perspectives on the epidemiology of mental disorders. Cambridge, United Kingdom: Cambridge University Press; 2008.
World Health Organisation. Study on Global Ageing and Adult Health (SAGE) Pilot Study 2005 Data from the Data Archive of Social Research on Aging. Los Altos, United States: Sociometrics Corporation. Available from: http://home.socio.com/age2728.php
Zwierzynska K, Wolke D, Lereya TS. Peer victimization in childhood and internalizing problems in adolescence: a prospective longitudinal study. J Abnorm Child Psychol. 2013; 41: 309–23.

3. Grouping of the states of India based on SDI, 2017

State group (population in 2017)	States of India	SDI in 2017
**Low SDI states (675 million)**	Bihar	0.43
	Madhya Pradesh	0.49
	Jharkhand	0.49
	Uttar Pradesh	0.49
	Rajasthan	0.49
	Chhattisgarh	0.51
	Odisha	0.52
	Assam	0.53
**Middle SDI states (387 million)**	Andhra Pradesh	0.54
	West Bengal	0.54
	Tripura	0.54
	Arunachal Pradesh	0.56
	Meghalaya	0.56
	Karnataka	0.57
	Telangana	0.58
	Gujarat	0.58
	Manipur	0.59
	Jammu and Kashmir*	0.59
	Haryana	0.60
**High SDI states (318 million)**	Uttarakhand	0.61
	Tamil Nadu	0.62
	Mizoram	0.62
	Maharashtra	0.62
	Punjab	0.62
	Sikkim	0.63
	Nagaland	0.63
	Himachal Pradesh	0.63
	Union territories other than Delhi	0.65
	Kerala	0.66
	Delhi	0.72
	Goa	0.74

*The state of Jammu and Kashmir was divided into two union territories in August 2019; as we are reporting findings up to 2017, we report findings for the state of Jammu and Kashmir.
SDI as computed by GBD in 2017 as described elsewhere (Lancet 2018; 392: 1995-2051).
SDI=Socio-demographic Index.

4. Prevalence of mental disorders in the states of India, 2017

States of India	Prevalence per 100,000 (95% uncertainty interval)									
	**Idiopathic developmental intellectual disability**	**Depressive disorders**	**Anxiety disorders**	**Conduct disorder**	**Bipolar disorder**	**Attention-deficit/hyperactivity disorder**	**Autism spectrum disorders**	**Schizophrenia**	**Eating disorders**	**Other mental disorders**
**India**	**4481 (2990 to 5987)**	**3310 (3070 to 3610)**	**3250 (2984 to 3542)**	**797 (625 to 997)**	**554 (478 to 652)**	**415 (341 to 498)**	**348 (311 to 386)**	**251 (219 to 286)**	**169 (132 to 210)**	**1766 (1506 to 2010)**
**Low SDI**	**5403 (3702 to 7137)**	**2860 (2645 to 3127)**	**3080 (2825 to 3363)**	**897 (703 to 1119)**	**518 (446 to 611)**	**422 (345 to 510)**	**353 (315 to 393)**	**224 (195 to 254)**	**145 (114 to 180)**	**1622 (1383 to 1848)**
Bihar	6339 (4388 to 8325)	2532 (2337 to 2773)	3052 (2796 to 3359)	974 (765 to 1217)	499 (431 to 584)	437 (357 to 529)	357 (318 to 396)	208 (181 to 239)	123 (96 to 155)	1534 (1308 to 1748)
Madhya Pradesh	5216 (3550 to 6912)	2886 (2654 to 3165)	2810 (2555 to 3094)	841 (660 to 1049)	521 (447 to 616)	413 (337 to 498)	351 (312 to 392)	231 (201 to 263)	151 (119 to 188)	1658 (1413 to 1888)
Jharkhand	4940 (3307 to 6592)	2907 (2677 to 3177)	3329 (3051 to 3646)	983 (774 to 1215)	522 (451 to 615)	421 (344 to 509)	351 (313 to 390)	229 (199 to 261)	153 (121 to 191)	1634 (1392 to 1862)
Uttar Pradesh	5503 (3777 to 7294)	2734 (2516 to 3007)	3039 (2778 to 3330)	927 (727 to 1158)	510 (438 to 604)	431 (352 to 521)	355 (318 to 397)	215 (187 to 245)	140 (109 to 178)	1586 (1353 to 1807)
Rajasthan	4898 (3318 to 6501)	2750 (2542 to 3012)	3265 (2987 to 3578)	870 (682 to 1087)	530 (453 to 624)	420 (343 to 508)	354 (317 to 398)	231 (201 to 264)	162 (126 to 203)	1633 (1393 to 1861)
Chhattisgarh	4738 (3233 to 6295)	2791 (2563 to 3063)	2892 (2635 to 3191)	801 (627 to 998)	541 (466 to 634)	401 (328 to 483)	345 (306 to 384)	242 (211 to 275)	165 (128 to 207)	1706 (1453 to 1942)
Odisha	4692 (3214 to 6205)	4159 (3841 to 4546)	3330 (3057 to 3641)	738 (578 to 920)	553 (474 to 645)	383 (312 to 460)	341 (302 to 379)	256 (223 to 291)	159 (125 to 201)	1822 (1556 to 2072)
Assam	5121 (3460 to 6767)	3271 (2988 to 3618)	3222 (2943 to 3540)	825 (647 to 1030)	528 (454 to 622)	410 (336 to 495)	347 (309 to 386)	237 (206 to 272)	154 (120 to 194)	1702 (1448 to 1940)
**Middle SDI**	**3952 (2594 to 5293)**	**3652 (3393 to 3980)**	**3376 (3102 to 3681)**	**709 (554 to 887)**	**585 (505 to 688)**	**392 (321 to 470)**	**346 (309 to 384)**	**272 (237 to 311)**	**185 (146 to 232)**	**1873 (1598 to 2131)**
Andhra Pradesh	3866 (2522 to 5170)	4563 (4235 to 4999)	3462 (3180 to 3783)	680 (534 to 847)	599 (519 to 703)	374 (306 to 450)	341 (304 to 380)	279 (244 to 318)	181 (140 to 230)	1936 (1653 to 2200)
West Bengal	4612 (3129 to 6134)	3291 (3042 to 3588)	3480 (3199 to 3787)	717 (563 to 894)	591 (509 to 692)	384 (314 to 463)	344 (306 to 383)	276 (239 to 317)	172 (134 to 219)	1904 (1624 to 2166)
Tripura	4560 (3082 to 6069)	3134 (2898 to 3434)	3391 (3105 to 3710)	734 (575 to 916)	589 (506 to 692)	391 (320 to 471)	345 (309 to 383)	270 (235 to 310)	172 (135 to 216)	1871 (1595 to 2130)
Arunachal Pradesh	4100 (2705 to 5525)	3421 (3148 to 3752)	3121 (2848 to 3443)	913 (715 to 1139)	532 (450 to 636)	435 (355 to 526)	356 (316 to 395)	230 (199 to 266)	186 (147 to 235)	1588 (1353 to 1813)
Meghalaya	4755 (3170 to 6331)	3340 (3089 to 3649)	3117 (2846 to 3439)	961 (754 to 1202)	527 (447 to 624)	441 (361 to 534)	354 (315 to 396)	220 (191 to 254)	171 (135 to 215)	1544 (1316 to 1760)
Karnataka	3736 (2448 to 4990)	3661 (3366 to 4051)	3408 (3124 to 3727)	585 (426 to 761)	589 (510 to 694)	401 (332 to 488)	344 (307 to 383)	275 (241 to 315)	188 (149 to 236)	1900 (1621 to 2162)
Telangana	3709 (2450 to 5028)	4356 (4045 to 4747)	3408 (3126 to 3729)	709 (557 to 884)	591 (509 to 698)	384 (314 to 463)	343 (308 to 383)	275 (239 to 314)	205 (160 to 258)	1888 (1611 to 2148)
Gujarat	3560 (2342 to 4830)	3233 (2977 to 3557)	3176 (2911 to 3458)	754 (591 to 942)	573 (494 to 673)	395 (322 to 475)	350 (312 to 388)	268 (234 to 307)	192 (150 to 241)	1833 (1564 to 2086)
Manipur	4676 (3114 to 6243)	3613 (3345 to 3929)	3760 (3440 to 4109)	793 (623 to 990)	577 (493 to 684)	402 (327 to 484)	347 (310 to 388)	252 (219 to 288)	154 (120 to 193)	1783 (1518 to 2031)
Jammu and Kashmir*	4190 (2783 to 5624)	2926 (2702 to 3198)	3262 (2986 to 3580)	874 (686 to 1092)	570 (488 to 671)	425 (347 to 512)	357 (316 to 400)	251 (218 to 288)	169 (132 to 211)	1746 (1489 to 1990)
Haryana	3190 (2050 to 4349)	3693 (3430 to 4022)	3255 (2982 to 3561)	788 (618 to 986)	562 (484 to 658)	408 (333 to 492)	355 (315 to 394)	261 (227 to 299)	206 (163 to 258)	1784 (1521 to 2031)
**High SDI**	**3168 (2034 to 4308)**	**3850 (3570 to 4189)**	**3460 (3177 to 3769)**	**694 (546 to 864)**	**592 (511 to 696)**	**427 (355 to 511)**	**339 (305 to 376)**	**285 (249 to 324)**	**199 (157 to 248)**	**1942 (1659 to 2207)**
Uttarakhand	3429 (2198 to 4658)	3032 (2811 to 3313)	3327 (3046 to 3643)	818 (643 to 1022)	565 (486 to 664)	406 (332 to 490)	347 (309 to 385)	256 (224 to 291)	197 (154 to 247)	1779 (1517 to 2025)
Tamil Nadu	3289 (2124 to 4463)	4796 (4446 to 5215)	3431 (3141 to 3768)	626 (490 to 779)	560 (477 to 675)	407 (337 to 486)	338 (303 to 376)	289 (253 to 331)	194 (151 to 242)	1993 (1705 to 2265)
Mizoram	4044 (2657 to 5423)	2852 (2642 to 3117)	3285 (3003 to 3606)	822 (644 to 1027)	570 (487 to 674)	410 (335 to 495)	349 (311 to 388)	253 (220 to 290)	180 (140 to 225)	1735 (1477 to 1976)
Maharashtra	3282 (2109 to 4479)	3673 (3401 to 4002)	3404 (3125 to 3718)	746 (582 to 927)	594 (514 to 699)	510 (425 to 610)	347 (310 to 386)	279 (244 to 319)	202 (159 to 252)	1914 (1634 to 2178)
Punjab	3243 (2071 to 4408)	3082 (2841 to 3365)	3234 (2959 to 3503)	702 (550 to 876)	594 (514 to 697)	383 (313 to 461)	349 (312 to 387)	281 (245 to 321)	191 (150 to 238)	1940 (1658 to 2206)
Sikkim	2989 (1883 to 4114)	3363 (3104 to 3681)	3391 (3100 to 3726)	744 (585 to 927)	605 (518 to 711)	404 (331 to 487)	353 (316 to 392)	287 (248 to 329)	245 (193 to 308)	1900 (1618 to 2166)
Nagaland	3832 (2527 to 5179)	3019 (2785 to 3293)	3214 (2939 to 3533)	924 (725 to 1154)	553 (474 to 653)	435 (355 to 525)	355 (318 to 396)	238 (207 to 274)	183 (144 to 228)	1654 (1409 to 1887)
Himachal Pradesh	3186 (2058 to 4362)	3580 (3314 to 3902)	3471 (3189 to 3809)	681 (535 to 850)	602 (523 to 703)	370 (303 to 446)	254 (215 to 294)	286 (248 to 329)	193 (151 to 245)	1953 (1669 to 2218)
UTs other than Delhi†	2777 (1734 to 3799)	3838 (3545 to 4210)	3472 (3173 to 3805)	682 (535 to 850)	637 (548 to 743)	352 (285 to 427)	354 (316 to 394)	307 (267 to 350)	229 (179 to 287)	1995 (1698 to 2273)
Kerala	2786 (1758 to 3821)	3897 (3602 to 4249)	4035 (3702 to 4393)	588 (453 to 741)	647 (564 to 753)	274 (221 to 337)	311 (274 to 347)	302 (265 to 342)	176 (139 to 222)	2014 (1734 to 2286)
Delhi	2391 (1456 to 3301)	2905 (2670 to 3192)	3374 (3091 to 3696)	734 (576 to 915)	600 (516 to 699)	399 (326 to 481)	354 (315 to 394)	289 (251 to 331)	247 (190 to 316)	1892 (1611 to 2155)
Goa	1953 (1133 to 2785)	3821 (3520 to 4161)	3307 (2982 to 3679)	589 (463 to 735)	657 (570 to 765)	184 (128 to 261)	341 (303 to 380)	328 (287 to 373)	255 (201 to 318)	2101 (1800 to 2384)

*The state of Jammu and Kashmir was divided into two union territories in August 2019; as we are reporting findings up to 2017, we report findings for the state of Jammu and Kashmir.

†Union territories

5. Relationship of the prevalence of depressive disorders, anxiety disorders, schizophrenia, and bipolar disorder with suicide death rate in states of India, 2017

Depressive disorders and suicide death rate

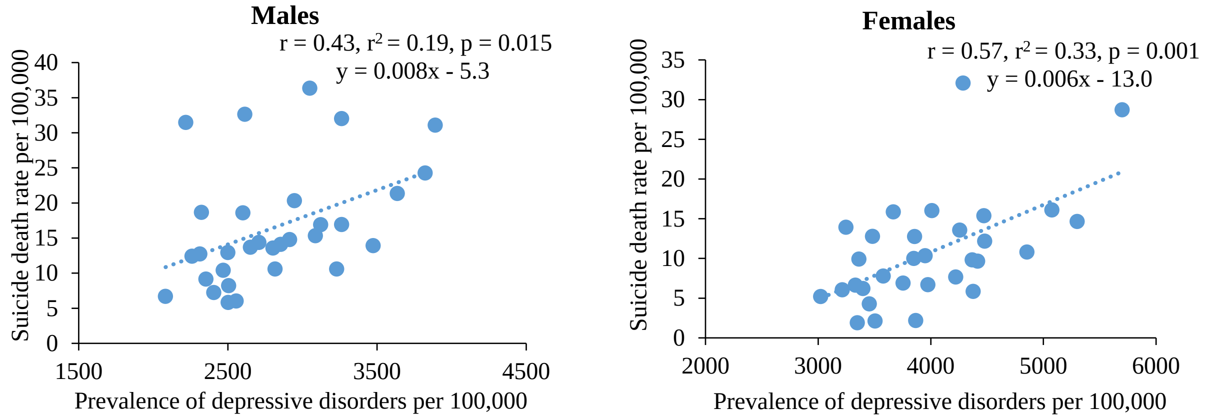

Anxiety disorders and suicide death rate

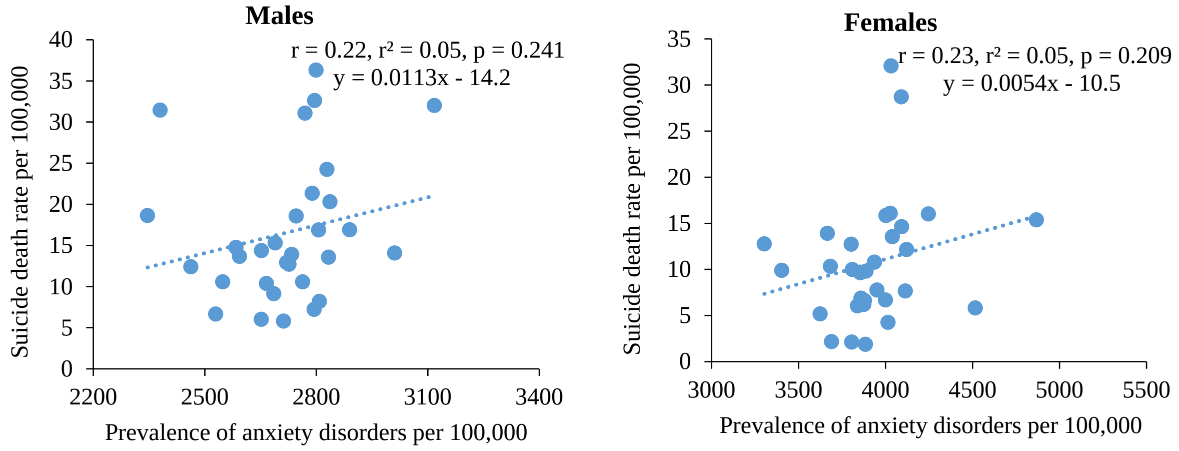

Schizophrenia and suicide death rate

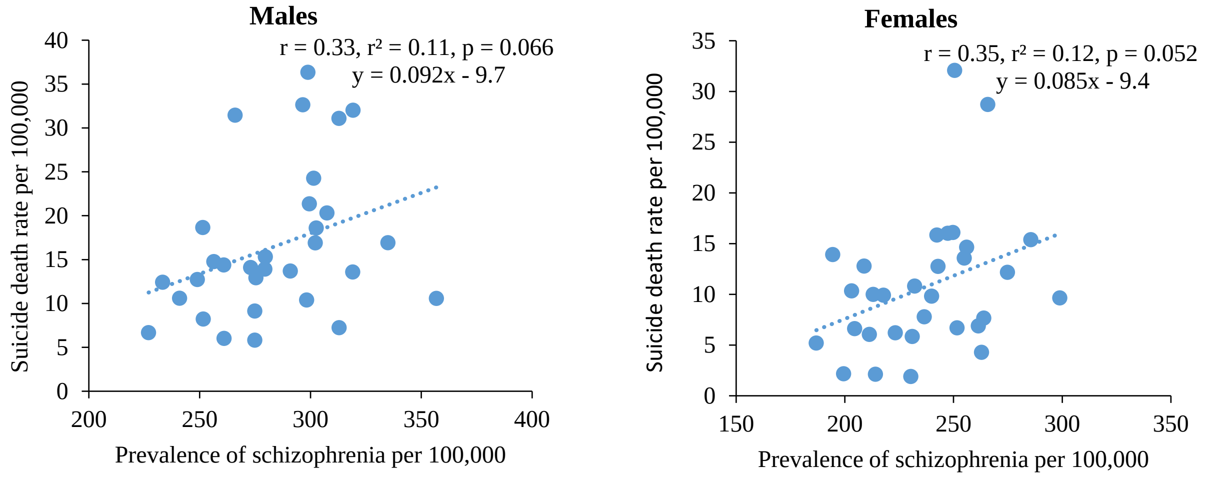

Bipolar disorder and suicide death rate

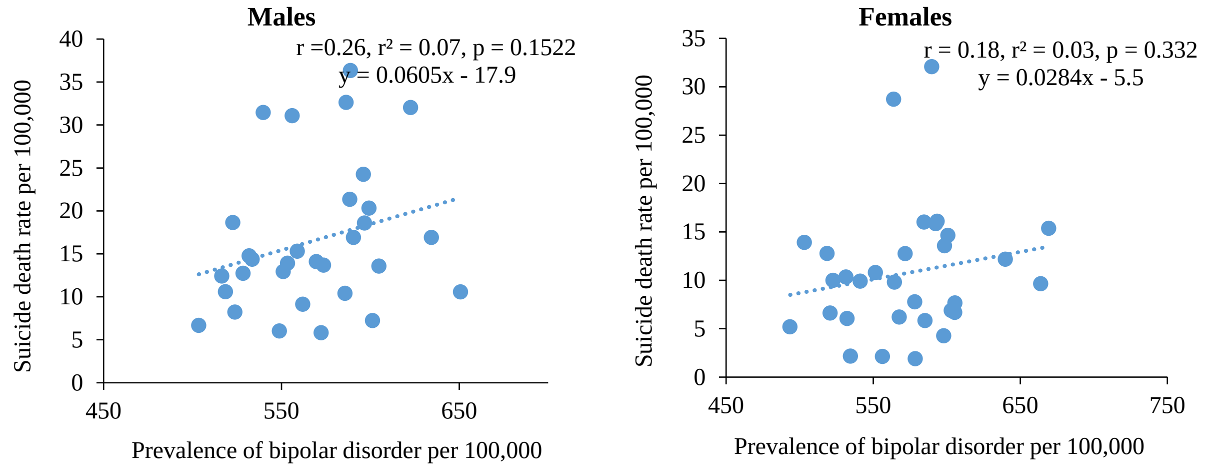

6. Age-specific prevalence of mental disorders in India, by sex, 2017

Age groups (years)	Prevalence per 100 (95% uncertainty interval)																	
	Idiopathic developmental intellectual disability		Depressive disorders		Anxiety disorders		Conduct disorder		Bipolar disorder		Attention-deficit/hyperactivity disorder		Autism spectrum disorders		Schizophrenia		Eating disorders	
	Males	Females	Males	Females	Males	Females	Males	Females	Males	Females	Males	Females	Males	Females	Males	Females	Males	Females
<5	5.78 (3.96 to 7.66)	5.90 (4.04 to 7.74)			0.07 (0.06 to 0.08)	0.10 (0.08 to 0.12)					0.09 (0.07 to 0.11)	0.04 (0.03 to 0.05)	0.58 (0.52 to 0.64)	0.22 (0.20 to 0.25)				
5 to 9	5.77 (3.97 to 7.63)	5.80 (4.00 to 7.58)	0.06 (0.04 to 0.09)	0.09 (0.06 to 0.13)	0.97 (0.80 to 1.14)	1.43 (1.20 to 1.70)	1.68 (1.12 to 2.42)	0.83 (0.54 to 1.18)			0.96 (0.74 to 1.19)	0.38 (0.29 to 0.49)	0.57 (0.51 to 0.63)	0.22 (0.19 to 0.24)				
10 to 14	5.55 (3.76 to 7.33)	5.47 (3.73 to 7.19)	0.71 (0.54 to 0.94)	0.98 (0.74 to 1.30)	2.34 (1.96 to 2.79)	3.49 (2.89 to 4.16)	4.57 (3.49 to 6.00)	2.87 (2.03 to 3.90)	0.22 (0.16 to 0.29)	0.22 (0.16 to 0.29)	1.30(1.00 to 1.62)	0.52 (0.39 to 0.66)	0.55 (0.49 to 0.61)	0.21 (0.18 to 0.23)	0.01 (0.00 to 0.01)	0.00 (0.00 to 0.01)	0.05 (0.03 to 0.07)	0.10 (0.07 to 0.14)
15 to 19	5.33 (3.59 to 7.06)	5.14 (3.48 to 6.80)	1.95 (1.58 to 2.41)	2.72 (2.19 to 3.36)	2.84 (2.44 to 3.29)	4.19 (3.55 to 4.93)	3.56 (2.53 to 4.65)	1.84 (1.27 to 2.62)	0.73 (0.55 to 0.95)	0.73 (0.54 to 0.95)	1.05 (0.83 to 1.31)	0.42 (0.33 to 0.53)	0.53 (0.47 to 0.59)	0.20 (0.18 to 0.22)	0.05 (0.04 to 0.07)	0.04 (0.03 to 0.06)	0.18 (0.11 to 0.27)	0.35 (0.24 to 0.50)
20 to 24	5.07 (3.42 to 6.74)	4.77 (3.21 to 6.30)	2.79 (2.31 to 3.40)	3.86 (3.17 to 4.67)	2.87 (2.37 to 3.34)	4.1 (3.38 to 4.91)	0.66 (0.42 to 0.94)	0.22 (0.12 to 0.35)	0.74 (0.56 to 0.95)	0.75 (0.57 to 0.96)	0.78 (0.62 to 0.98)	0.32 (0.25 to 0.40)	0.51 (0.46 to 0.57)	0.19 (0.17 to 0.22)	0.22 (0.16 to 0.29)	0.17 (0.12 to 0.23)	0.21 (0.13 to 0.34)	0.45 (0.30 to 0.66)
25 to 29	4.77 (3.20 to 6.36)	4.36 (2.93 to 5.77)	2.95 (2.48 to 3.47)	4.16 (3.45 to 4.92)	3.01 (2.55 to 3.48)	4.23 (3.51 to 5.04)			0.70 (0.55 to 0.87)	0.71 (0.56 to 0.90)	0.61 (0.49 to 0.75)	0.25 (0.20 to 0.32)	0.50 (0.45 to 0.55)	0.19 (0.16 to 0.21)	0.41 (0.32 to 0.52)	0.32 (0.25 to 0.41)	0.24 (0.17 to 0.33)	0.48 (0.35 to 0.66)
30 to 34	4.51 (3.01 to 6.03)	4.01 (2.68 to 5.34)	3.19 (2.63 to 3.76)	4.51 (3.70 to 5.36)	3.26 (2.86 to 3.68)	4.60 (3.99 to 5.31)			0.71 (0.56 to 0.86)	0.72 (0.57 to 0.88)	0.50 (0.40 to 0.61)	0.21 (0.17 to 0.26)	0.48 (0.43 to 0.54)	0.18 (0.16 to 0.20)	0.52 (0.43 to 0.64)	0.41 (0.34 to 0.51)	0.29 (0.19 to 0.42)	0.52 (0.38 to 0.69)
35 to 39	4.27 (2.85 to 5.75)	3.70 (2.47 to 4.96)	3.72 (3.23 to 4.35)	5.14 (4.42 to 6.05)	3.56 (3.15 to 4.00)	5.10 (4.45 to 5.81)			0.76 (0.62 to 0.93)	0.78 (0.64 to 0.95)	0.41 (0.33 to 0.51)	0.18 (0.14 to 0.22)	0.47 (0.42 to 0.53)	0.18 (0.16 to 0.20)	0.57 (0.48 to 0.69)	0.46 (0.39 to 0.56)	0.24 (0.16 to 0.34)	0.45 (0.32 to 0.60)
40 to 44	4.04 (2.69 to 5.45)	3.4 0(2.26 to 4.57)	4.15 (3.47 to 4.97)	5.69 (4.77 to 6.86)	3.81 (3.28 to 4.44)	5.59 (4.78 to 6.48)			0.84 (0.67 to 1.05)	0.85 (0.68 to 1.06)	0.34 (0.27 to 0.43)	0.15 (0.12 to 0.19)	0.46 (0.41 to 0.51)	0.17 (0.15 to 0.19)	0.57 (0.50 to 0.67)	0.48 (0.41 to 0.56)	0.12 (0.08 to 0.17)	0.31 (0.22 to 0.42)
45 to 49	3.8 (2.52 to 5.16)	3.11 (2.03 to 4.19)	4.50 (3.92 to 5.19)	6.21 (5.42 to 7.16)	3.85 (3.34 to 4.45)	5.72 (4.82 to 6.65)			0.86 (0.71 to 1.07)	0.87 (0.71 to 1.08)	0.28 (0.22 to 0.35)	0.13 (0.10 to 0.16)	0.45 (0.40 to 0.50)	0.17 (0.15 to 0.18)	0.55 (0.48 to 0.64)	0.47 (0.41 to 0.54)	0.06 (0.04 to 0.09)	0.20 (0.15 to 0.28)
50 to 54	3.56 (2.34 to 4.85)	2.81 (1.82 to 3.81)	4.93 (4.36 to 5.55)	6.84 (6.04 to 7.72)	3.77 (3.32 to 4.32)	5.56 (4.75 to 6.43)			0.85 (0.67 to 1.09)	0.85 (0.67 to 1.09)	0.22 (0.17 to 0.28)	0.10 (0.08 to 0.13)	0.44 (0.39 to 0.49)	0.16 (0.14 to 0.18)	0.51 (0.45 to 0.59)	0.44 (0.39 to 0.51)		
55 to 59	3.32 (2.17 to 4.53)	2.51 (1.59 to 3.46)	5.40 (4.68 to 6.14)	7.55 (6.46 to 8.67)	3.64 (3.18 to 4.17)	5.23 (4.53 to 5.98)			0.81 (0.64 to 1.05)	0.80 (0.63 to 1.04)	0.15 (0.11 to 0.20)	0.07 (0.05 to 0.10)	0.43 (0.38 to 0.47)	0.16 (0.14 to 0.17)	0.46 (0.40 to 0.52)	0.40 (0.35 to 0.46)		
60 to 64	3.09 (1.99 to 4.22)	2.21 (1.37 to 3.05)	5.90(5.05 to 6.76)	8.30 (7.07 to 9.52)	3.48 (2.93 to 4.09)	4.83 (4.10 to 5.57)			0.75 (0.63 to 0.91)	0.74 (0.61 to 0.90)	0.09 (0.06 to 0.12)	0.05 (0.03 to 0.07)	0.41 (0.37 to 0.46)	0.15 (0.13 to 0.17)	0.40 (0.35 to 0.45)	0.35 (0.31 to 0.40)		
65 to 69	2.84 (1.81 to 3.88)	1.91 (1.15 to 2.66)	6.25 (5.50 to 7.03)	8.84 (7.75 to 9.86)	3.30 (2.76 to 3.88)	4.54 (3.76 to 5.32)			0.68 (0.55 to 0.81)	0.66 (0.53 to 0.79)	0.05 (0.03 to 0.07)	0.03 (0.02 to 0.04)	0.40 (0.36 to 0.45)	0.15 (0.13 to 0.16)	0.33 (0.29 to 0.37)	0.30 (0.26 to 0.34)		
70 to 74	2.49 (1.56 to 3.44)	1.57 (0.91 to 2.23)	6.43 (5.68 to 7.25)	9.12 (8.07 to 10.23)	3.11 (2.67 to 3.59)	4.37 (3.67 to 5.09)			0.58 (0.47 to 0.70)	0.56 (0.46 to 0.68)	0.02 (0.01 to 0.04)	0.01 (0.01 to 0.02)	0.39 (0.35 to 0.44)	0.14 (0.13 to 0.16)	0.26 (0.23 to 0.30)	0.24 (0.21 to 0.27)		
75 to 79	2.08 (1.24 to 2.92)	1.22 (0.63 to 1.80)	6.53 (5.65 to 7.60)	9.26 (8.05 to 10.63)	2.90 (2.53 to 3.29)	4.25 (3.69 to 4.87)			0.49 (0.40 to 0.58)	0.47 (0.39 to 0.56)	0.01 (0.00 to 0.01)	0.01 (0.00 to 0.01)	0.38 (0.34 to 0.43)	0.14 (0.12 to 0.15)	0.20 (0.17 to 0.22)	0.18 (0.16 to 0.21)		
≥80	1.86 (1.18 to 2.56)	1.13 (0.64 to 1.61)	6.14 (5.29 to 7.23)	8.45 (7.27 to 9.83)	2.53 (2.17 to 2.93)	4.06 (3.55 to 4.61)			0.37 (0.30 to 0.45)	0.36 (0.30 to 0.44)			0.37 (0.33 to 0.42)	0.13 (0.12 to 0.15)	0.12 (0.10 to 0.14)	0.12 (0.10 to 0.14)		
